# Predicting future dynamics from short-term time series using an Anticipated Learning Machine

**DOI:** 10.1093/nsr/nwaa025

**Published:** 2020-02-19

**Authors:** Chuan Chen, Rui Li, Lin Shu, Zhiyu He, Jining Wang, Chengming Zhang, Huanfei Ma, Kazuyuki Aihara, Luonan Chen

**Affiliations:** School of Data and Computer Science, Sun Yat-sen University, Guangzhou 510275, China; School of Data and Computer Science, Sun Yat-sen University, Guangzhou 510275, China; School of Data and Computer Science, Sun Yat-sen University, Guangzhou 510275, China; School of Data and Computer Science, Sun Yat-sen University, Guangzhou 510275, China; School of Data and Computer Science, Sun Yat-sen University, Guangzhou 510275, China; Center for Excellence in Molecular Cell Science, Shanghai Institute of Biochemistry and Cell Biology, Chinese Academy of Sciences, Shanghai 200031, China; School of Mathematical Sciences, Soochow University, Suzhou 215006, China; Institute of Industrial Science, The University of Tokyo, Tokyo 153–8505, Japan; International Research Center for Neurointelligence, The University of Tokyo, Tokyo 113-0033, Japan; Center for Excellence in Molecular Cell Science, Shanghai Institute of Biochemistry and Cell Biology, Chinese Academy of Sciences, Shanghai 200031, China; Center for Excellence in Animal Evolution and Genetics, Chinese Academy of Sciences, Kunming 650223, China; Key Laboratory of Systems Biology, Hangzhou Institute for Advanced Study, University of Chinese Academy of Sciences, Hangzhou 310024, China; Shanghai Research Center for Brain Science and Brain-Inspired Intelligence, Shanghai 201210, China

**Keywords:** dynamics-based machine learning, delay embedding theory, short-term time series prediction, dynamics-based data science

## Abstract

Predicting time series has significant practical applications over different disciplines. Here, we propose an Anticipated Learning Machine (ALM) to achieve precise future-state predictions based on short-term but high-dimensional data. From non-linear dynamical systems theory, we show that ALM can transform recent correlation/spatial information of high-dimensional variables into future dynamical/temporal information of any target variable, thereby overcoming the small-sample problem and achieving multistep-ahead predictions. Since the training samples generated from high-dimensional data also include information of the unknown future values of the target variable, it is called anticipated learning. Extensive experiments on real-world data demonstrate significantly superior performances of ALM over all of the existing 12 methods. In contrast to traditional statistics-based machine learning, ALM is based on non-linear dynamics, thus opening a new way for dynamics-based machine learning.

## Teaser

We propose Anticipated Learning Machine, a dynamics-based data-driven method, to achieve future-state predictions based on short-term but high-dimensional data, which transforms recent spatial information of high-dimensional variables into future temporal information of any target variable.

## INTRODUCTION

Making an accurate prediction based on observed data, in particular from short-term time series, is of much concern in various disciplines, arising from molecular biology, neuroscience geoscience to atmospheric sciences [1–6] due to either data availability or time-variant non-stationarity. Based on the source of predictability, various methods have been proposed [7–11], such as statistical regression methods including ARIMA [12], robust regression [13] and exponential smoothing [14], and machine learning methods including the long short-term memory network [15] and reservoir computing [16–19]. For the statistical forecast for time series, there are also many theoretical works that focus on regret minimization [20]. However, most of such methods require sufficiently long measurements of time series and there is no effective method available for prediction with short-term time series because of a lack of information.

**Figure 1.**
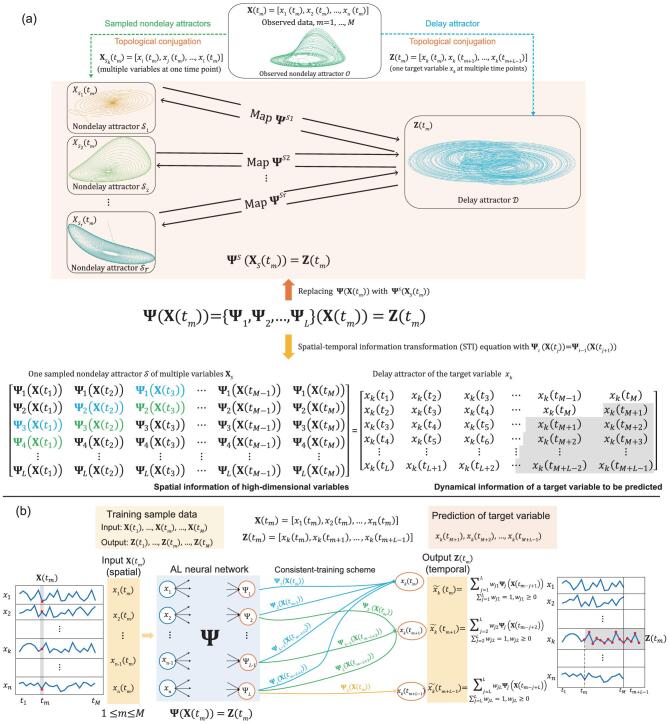
(a) The general principle of the Anticipated Learning Machine (ALM). The observed attractor, a delay attractor and sampled nondelay attractors are all topologically conjugated with each other. Each sampled nondelay attractor preserves the dynamical information of the system in different ways. By integrating the information contained in these sampled nondelay attractors, we could find an accurate one-to-one map even under noise deterioration. (b) Anticipated Learning Machine. For each future value, those maps are co-trained into a unified map ${{\boldsymbol \Psi }}$. When the maps are trained, the weighted sum is used as the prediction. The predicted value is then used as the label when training other maps to predict the next time point. Clearly, ALM ${{\boldsymbol \Psi }}$ transforms spatial input **X**(*t_m_*) to temporal output **Z**(*t_m_*) (also see Supplementary Fig. 1) at each point *t_m_*.

On the other hand, little attention has been paid to prediction from short-term but high-dimensional data, which have become increasingly and widely available in many fields. Such short-term but high-dimensional data have rich information content due to the measured high-dimension variables, which can be exploited for the prediction. Actually, to transform the information of high-dimension data into the future evolution of a target variable, randomly distributed embedding (RDE) [21] has been theoretically derived based on delay embedding theory and further numerically validated by one-step-ahead predictions of a number of short-term time series. The challenging problem for this framework is how to solve the unknown non-linear map between the sampled nondelay attractors of high-dimensional variables and the delay attractor of one target variable, where each attractor is numerically represented by a series of data points (Fig. 1a). In this work, by accurately learning the non-linear RDE map or spatial-temporal information-transformation (STI) equation so as to make precise predictions, we propose an anticipated-learning (AL) neural network, namely the Anticipated Learning Machine (ALM), based on the observed short-term high-dimensional data. From the generalized embedding theory [22–24], we show that ALM can transform recent correlation information of high-dimensional variables to future dynamical information of any target variable by the AL neural network, thereby overcoming the short-term data problem and also achieving multistep-ahead predictions.

Specifically, the AL neural network is a multi-layer neural network (Fig. 1b), where high-dimensional variables are taken as input neurons (multiple variables but at a single time point due to nondelay embedding) but a target variable is taken as output neurons (single variable but at multiple time points due to delay embedding). ALM first generates a large number of sampled nondelay attractors by randomly chosen variables from high-dimensional data and then each sampled nondelay attractor (input) paired with the delay attractor of the target variable (output) is taken as one training sample to train the neural network. In such a way, the AL neural network can be well trained to represent the RDE map by a large number of the generated training samples with the Dropout [25] scheme and our ‘consistent-training scheme’, thus predicting the target variable in an accurate and robust manner, even with short-term data. Actually, the human brain has such AL or predictive action ability even with a small number of samples, which is different from the current deep learning scheme that mainly depends on a large number of samples to experience all situations. Compared with the traditional neural networks that excavate the historical statistics of the original high-dimensional system and thus require a large number of samples, ALM efficiently and robustly reconstructs its dynamics even with a small number of samples by constraining to a low dimension space, which is actually an inherent property of such a dissipative system. Thus, precise prediction can be made even from short-term high-dimensional data due to such dynamical features. Extensive experiments on the short-term high-dimensional data from both synthetic and real-world systems have demonstrated significantly superior performances of ALM over existing methods, which indicates that ALM makes high-dimensional data a rich source of dynamical information to compensate for the observed short-term data. In contrast to traditional statistics-based machine learning, ALM is based on non-linear dynamics to transform the spatial information of the all measured high-dimensional variables into the temporal evolution of the target variable by learning the RDE map, thus opening a new way for dynamics-based machine learning or ‘intelligent’ AL.

Note that long-term data measured from many complex systems such as biological systems and financial systems can be also considered as short-term data, since generally those systems are highly time-varying also with many hidden variables, and thus the effective predictions of their future evolution depend mainly on the recent short-term data. Therefore, ALM provides a general framework to learn and predict complex systems by exploring the intertwined high-dimensional information of recent short-term data rather than the past long-term data, thus also alleviating the time-varying problem. In addition, current deep learning methods generally require a large amount of data to experience behaviors in almost all situations, but we show in this paper that ALM can make accurate predictions of such dynamical behaviors that even never appear in the observed data due to the STI transformation and RDE.

## RESULTS

### Indicators for evaluation

As shown in Supplementary Tables I–IX , we use five criteria/indicators to measure the effectiveness of ALM. The first four criteria are Mean Absolute Error (MAE), Root Mean Square Error (RMSE), Pearson and Spearman correlation coefficients. MAE and RMSE reflect the numerical errors between the true values and the predicted ones, and the other two correlation coefficients show the errors in the trends. Therefore, we construct the composite indicator over all the above criteria so as to achieve a comprehensive measurement of numerical values and trends. The higher the composite indicator is, the better the model performs.

### Synthetic data set

**Figure 2.**
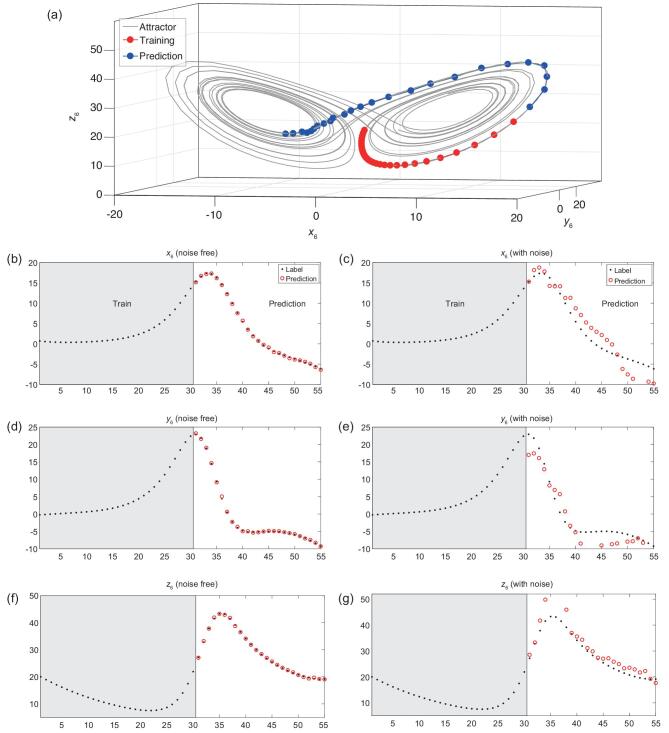
The dynamics and attractor of the 90D time-variant-coupled Lorentz system. (a) The predicted evolution in the attractor. (b, d, f) The predictions on the first Lorentz system (noise-free) for variables ${x_6}$, ${y_6}$ and ${z_6}$. (c, e, g) The predictions on the first Lorentz system (with noise) for variables ${x_6}$, ${y_6}$ and ${z_6}$. See Supplementary Figs 4–9 and Supplementary Tables I and II for the predictions by the other 12 methods.

To validate our model's ability to transform the high-dimensional information to the non-linear evolution of any target variable, we consider a 90D time-variant coupled Lorentz system (90 variables; see Section 2.1 in the Supplementary Materials). As shown in Fig. 2b, d and f, when there is no noise in the data, ALM predicts the non-periodic behavior (25 time points) of the Lorentz system accurately with only 30 training time points, which means that ALM can capture the system's dynamics with very short time series even for a time-variant system, and in particular can predict the un-experienced dynamics (as shown in Fig. 2a, ALM only learns the dynamics in one attractor, but correctly predicts the dynamics in another attractor), in contrast to traditional deep learning, which usually requires to learn almost all situations. To validate our model's robustness to noise, we add a Gaussian noise (mean = 0.0, standard deviation = 3.0) into the data and the result is shown in Fig. 2c, e and g. Although the performance deteriorates compared with the noise-free situation, ALM still captures the system's dynamics more efficiently than the other 12 methods (Supplementary Table II), which demonstrates ALM’s effectiveness to alleviate noise deterioration by using a large number of sampled nondelay attractors.

### Real-world data set

In the era of big data, high-dimensional data are ubiquitous in the real world. We apply ALM to various real-world data sets by comparing with all of the existing 12 methods (Figs 3–5, Supplementary Figs 4–30, Supplementary Tables I–IX and Sections 2–3).

**Figure 3.**
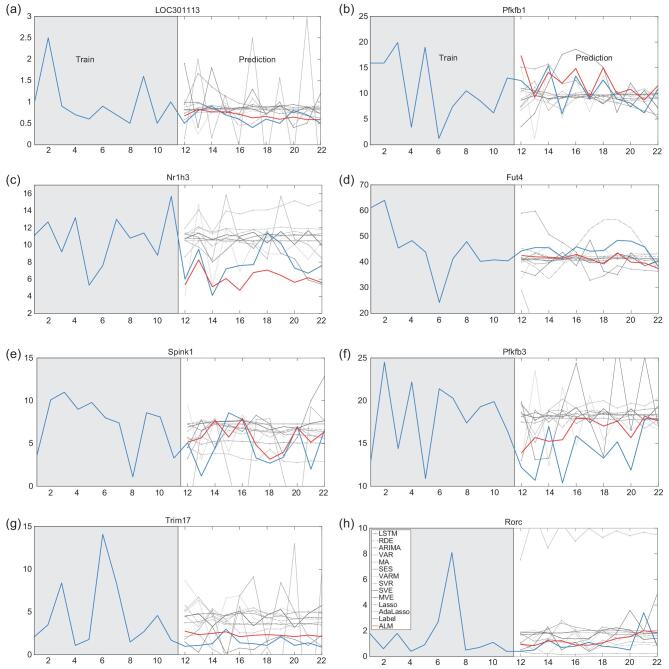
Predicting the dynamics of gene regulations. (a, c, e, g) Prediction on genes unrelated to circadian rhythm. (b, d, f, h) Prediction on genes related to circadian rhythm.

**Figure 4.**
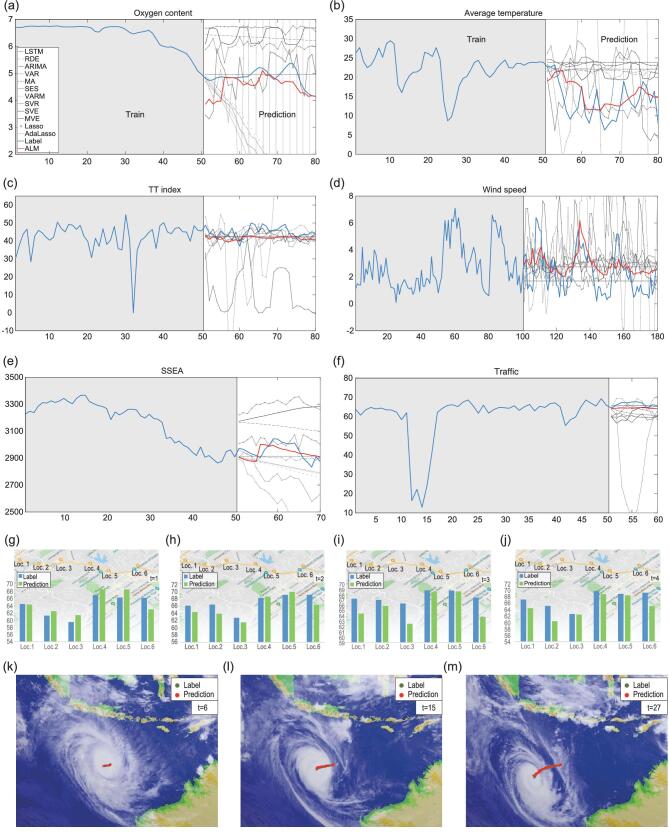
Predicting the dynamics of various real-world systems. (a) The oxygen content in a vessel. (b) The average temperature of the sea. (c) The TT index of the sea. (d) The wind speed at a wind station near Tokyo. (e) The stock index of SSEA. (f) The traffic flow on the highway of Los Angeles in Location 1. (g–j) The traffic flow in all locations across the first four time points. (k–m) The predicted route of the typhoon center.

**Figure 5.**
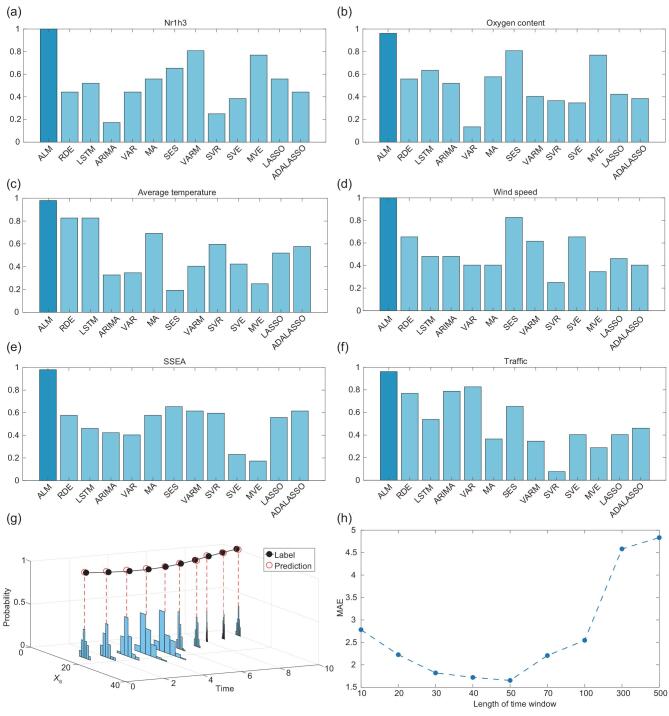
Analyses and comparisons with existing methods. The higher the composite indicator, the better the method performs. (a) Composite indicators of different methods on the gene data set. (b) Composite indicators of different methods on the plankton data set. (c) Composite indicators of different methods on the ground-ozone-level data set. (d) Composite indicators of different methods on the wind speed data set. (e) Composite indicators of different methods on the stock index data set. (f) Composite indicators of different methods on the traffic data set. (g) The distribution of predictions by our ALM. (h) Predictions under different lengths of the time window by ALM. Note that values of x-axis are unequal intervals.

#### Gene expression data

We first analyse a set of gene expression data for studying circadian rhythm, measured by Affymetrix microarray on the laboratory rat (*Rattus norvegicus*) cultured cells from suprachiasmatic nucleus (SCN), which consists of the expression of 31 099 genes with 22 time points [26] (31 099 dimensions/variables). As a consequence of a sophisticated gene regulation mechanism [27], all these genes form a high-dimensional dynamical system, which facilitates various biological functions. A biological system at a molecular level is a complex system that is generally characterized by many weak associations among variables like genes and proteins, and also by many hidden (unmeasured) variables; thus, it is difficult to be predicted or modeled. We apply ALM to predict gene dynamics based on the first 11 time points of the observed gene expression data. We select 8 genes to predict the remaining 11 time points, as shown in Fig. 3. Clearly, our ALM predicts the trends and values of gene expression more accurately than other methods (also see Supplementary Figs 10–17). Note that our model not only works well on genes relevant to circadian rhythm such as Fut4, but also on those irrelevant to circadian rhythm such as Spink1, which demonstrates ALM’s ability to transform the high-dimensional variables to the non-linear dynamics of any target variable. The Pearson coefficient on Nr1h3 is illustrated in Supplementary Fig. 33a and other scores are shown in Supplementary Table III and Supplementary Fig. 32a.

#### Plankton data set

The second data set is collected from an optical plankton counter and Conductivity-Temperature-Depth (CTD) mounted to a ScanFish platform that was towed and undulated behind the vessel [28]. This data set contains 58 attributes/variables and can be regarded as a small ecosystem. As shown in Fig. 4a, ALM predicts the trend of the oxygen content much better in contrast to the majority of other methods. The Pearson coefficient is illustrated in Supplementary Fig. 33b and other scores are shown in Supplementary Table IV and Supplementary Fig. 32b.

#### Climate data sets

The next two data sets of our analysis are climate data sets, which are known by their complex spatio-temporal characteristics.

The first data set is a 72-dimension ground-ozone-level data set collected from 1998 to 2004 at the Houston, Galveston and Brazoria areas [29]. We can predict the average temperature and TT Index well, as shown in Fig. 4b and c. The series of the average temperature and TT Index predicted by ALM get the highest scores on the composite indicators compared with other methods as shown in Fig. 5c, indicating that ALM can predict the future values efficiently. The Pearson coefficient on the average temperature is illustrated in Supplementary Fig. 33c and other scores are shown in Supplementary Table V and Supplementary Fig. 32c.

The second data set is the wind speed data set collected in Japan by the Japan Methodological Agency [30]. There are 155 wind stations in total and we select one station near Tokyo. As shown in Fig. 4d, ALM predicts the dynamics of the wind speed significantly more accurately than other methods. The Pearson coefficient is illustrated in Supplementary Fig. 33d and other scores are shown in Supplementary Table VI and Supplementary Fig. 32d.

#### Stock index data set

A 1130-dimension stock index data set is used to measure the effectiveness of our model on a highly unstable system. Due to the linkage effect in the stock market, different sectors of the stock market interact internally and form a complex system. We predict the Shanghai Stock Exchange A (SSEA) Share Index and illustrate the result in Fig. 4e. Clearly, ALM achieves higher accuracy than other methods, which means that ALM can capture the system's dynamics even if the system is highly unstable. The Pearson coefficient is illustrated in Supplementary Fig. 33e and other scores are shown in Supplementary Table VII and Supplementary Fig. 32e.

#### Traffic data set

This data set is the traffic speed collected from 207 loop detectors (variables) on the highway of Los Angeles County [31]. We train our ALM on 50 time points at 6 locations respectively and predict the next 10 time points. As shown in Fig. 4f–j (also see Supplementary Fig. 31), our ALM predicts the traffic flow accurately. The Pearson coefficient is illustrated in Supplementary Fig. 33f and other scores are shown in Supplementary Table VIII and Supplementary Fig. 32f.

#### Satellite cloud-image data set

This data set comes from the satellite cloud-image data recording the route of typhoon Marcus collected by the National Institute of Informatics [32]. The data set is composed of a series of 241 cloud images (2402 variables) starting from 15 March 2018 to 24 March 2018 with 1 image taken per hour. Based on 50 images, we make predictions of the tropical cyclone's central position for the next 27 time points. The predicted result is shown in Fig. 4k–m and a movie is given in the Supplementary Materials, which accurately predicts the real trajectory of the typhoon center.

All of the results above imply that ALM is able to make precise predictions by transforming the high-dimensional data into the future evolution of the target variable, opening a new way for dynamics-based machine learning in contrast to the traditional statistics-based machine learning. Theoretically, ALM can transform the high-dimensional (spatial) information into the dynamic (temporal) information as indicated in Equation (1) and Fig. 1, thus achieving accurate multistep-ahead prediction even with the small size of the measured samples (short-term time series data). Except RDE, which is for one-step-ahead prediction, all the other comparison methods are designed neither for short-term time series data nor for exploiting the dynamical information from high-dimensional data. Here, we should mention that ALM cannot have accurate predictions when encountering a sudden change in real-world data sets. The reason might lie in the fact that sudden changes in systems are usually caused by external factors, which are not included in the trained system.

### Analysis and comparison with existing methods

#### Distribution of prediction

Since each sampled nondelay attractor preserves the dynamical information of the entire system in a different way, when integrating the information sampled from different nondelay attractors, the resulting predictions could be different. To study the influence of randomness caused by the sampling mentioned above and the random initialization of the neural network, we plot the prediction distribution in Fig. 5g. For each prediction, 100 randomly initialized neural networks are trained on the Lorentz data set. Clearly, the shape of the distribution is similar to the Gaussian distribution and most predictions fall within a small range.

#### Time-invariant assumption for a short-term time series

Each ${{{\boldsymbol \Psi }}_i}$ is generally time-variant for many real-world systems but can be considered as time-invariant only when the time window or time series is short. To approximate the maximal length of the practical time window, we train ${{{\boldsymbol \Psi }}_i}$ on different sizes of the training time window and make a 10-steps-forward prediction for each time window by using the 155D wind speed data set. The result is illustrated in Fig. 5h. At the beginning, the time window is too short to train ${{{\boldsymbol \Psi }}_i}$, so the prediction is inaccurate. As the length increases, the performance first improves and then drops drastically. It means that, within a certain range, ${{{\boldsymbol \Psi }}_i}$ can be considered as time-invariant and trained by our method efficiently. When the time window becomes long, the system structure changes or ${{{\boldsymbol \Psi }}_i}$ changes. In other words, ${{{\boldsymbol \Psi }}_i}$ cannot be considered as time-invariant for such a long-term time series. This analysis indicates that long-term data may not be helpful for the prediction of a complex system (or time-variant system) and only recent data (short-term) are valuable for predicting the dynamics by exploiting high-dimensional information to compensate for the lack of data, which is actually a major advantage of ALM over existing methods. Certainly for a time-invariant system, ALM also works well for a long-term time series.

#### Comparison methods

We compare ALM with the following 12 existing methods with the detailed results listed in Section 3.1 in the Supplementary Materials: ARIMA [12]: a well-known autoregressive model for predicting future time series; VAR [33]: a vector autoregressive model, which captures the pairwise relationships among all variables; MA: moving average, which uses the unweighted mean of the previous data to make prediction; SES [14,34]: Holt-Winters exponential smoothing, which uses a weighted moving average with exponentially decreasing weights of the previous data to make predictions; VARM [35]: the basic process of the VARMAX (p, q, r) model, which includes the autoregressive process, the moving-average process and the independent exogenous terms (other unmodeled inputs); SVR [36]: Support Vector Regression, which uses support vector machine (SVM) to fit curves and perform regression analysis; SVE [37]: the classic single-variable embedding; MVE [38]: the recently proposed multi-view embedding; RDE [39]: the recently proposed method for short-term high-dimensional time series predictions; LSTM [15]: a famous neural network that is widely used in the field of time series analysis; Lasso [40]: the Lasso procedure is used to estimate the parameters of AR(p) and make predictions; AdaLasso [40]: the AdaLasso procedure is used to estimate the parameters of AR(p) and make predictions.

For each comparison method, its prediction is plotted in Supplementary Figs 4–30 and Supplementary Tables I–IX in the Supplementary Materials with Movie-Traffic & Movie-Satellite Images attached to the following link: https://github.com/AnticipatedLearningMachine/Anticipated-Learning-Machine.

## METHODS

### Map from each sampled nondelay attractor to delay attractor

There are rich information contents in high-dimensional data due to the intertwined interactions among a large number of variables, which can be explored to characterize the dynamics of the system as a source of predictability. Actually, to transform the correlation information of high-dimensional data into the future evolution of a target variable, RDE [21,39] has been theoretically derived based on the generalized embedding theory [22–24], which is briefly shown in Fig. 1a. Denote ${t_i} = \ t + {i}\,\,{\tau}$, with$\ \tau $ being the positive time delay, and a natural number $i\ > \ 0$, the observed *M* points on the system's observed attractor $\boldsymbol{\mathcal{O}}$ are denoted by ${{\bf X}} ( {{t_m}} ) \ = \ [ {{x_1}( {{t_m}} ),\ {x_2}( {{t_m}} ),\ \ldots ,\ {x_n}({{t_m}} )} ]\ $for $m\ = \ 1,2, \ldots ,\ M$, where ${x_1},\ {x_2},\ \ldots ,\ {x_n}\ $are *n* variables of the observed system. Given this observed nondelay attractor $\boldsymbol{\mathcal{O}}$, the delay attractor $\mathcal{D}$ is reconstructed by the delay coordinate map ${{\bf Z}} ( {{t_m}} ) \ = \ [ {{x_k}( {{t_m}} ),\ {x_k}( {{t_{m + 1}}} ),\ \ldots ,\ {x_k}( {{t_{m + L - 1}}} )} ]$, where ${x_k}\ $is a target variable to be predicted. The delay embedding theory [23,41] and the generalized embedding theory [22–24] reveal that the above reconstructed two attractors with an appropriate $L > 2d\ $(where $d$ is the box-counting dimension of the attractor) are topologically conjugated with each other (see Section 1.1–1.2 of the Supplementary Materials). Thus, there exists a one-to-one map between the observed nondelay attractor $\boldsymbol{\mathcal{O}}$ and the delay attractor ${\mathcal{D}}$ [21], i.e.

$$\begin{equation*}
{\boldsymbol {\Psi }}:{{\bf X}}( {{t_m}}) \to {{\bf Z}}( {{t_m}})\,{\rm{for}}\,m = 1,2, \ldots ,M,
\end{equation*}$$

where the domain is the observed nondelay attractor of the high-dimensional variables ${{\bf X}}( {{t_m}} )$ that are available or observed values from $m = 1$ to $m = M$, but the range is the delay attractor of the target variable ${x_k}$, which includes the unknown future values, i.e. ${x_k}( {{t_{M + 1}}} ),\ {x_k}( {{t_{M + 2}}} ),\ \ldots ,\ {x_k}( {{t_{M + L - 1}}} )\ $of the target variable. Thus, the map ${{\boldsymbol \Psi }}$ is to transform the information of a large number of variables to the future evolution of one target variable. The above transformation can be also expressed as a matrix form with ${{{\boldsymbol \Psi }}_i} ( {{{\bf X}}( {{t_j}} )} ) = {{{\boldsymbol \Psi }}_{i - 1}} ( {{{\bf X}}( {{t_{j + 1}}} )} )$ and ${{{\boldsymbol \Psi }}_i} ( {{{\bf X}}( {{t_j}} )} ) = {x_k} ( {{t_{i + j - 1}}} )$ (see Section 1.2 in the Supplementary Materials):

(1) \begin{eqnarray*}{\left[ {\begin{array}{@{}*{4}{c}@{}} {{{{\boldsymbol \Psi }}_1}( {{{\bf X}}( {{t_1}} )} )}&\,\,{{{{\boldsymbol \Psi }}_1}( {{{\bf X}}( {{t_2}})} )}&\,\, \cdots &\,\,{{{{\boldsymbol \Psi }}_1}( {{{\bf X}}( {{t_M}} )} )}\\ {{{{\boldsymbol \Psi }}_2}( {{{\bf X}}( {{t_1}} )} )}&\,\,{{{{\boldsymbol \Psi }}_2}( {{{\bf X}}( {{t_2}} )} )}&\,\, \cdots &\,\,{{{{\boldsymbol \Psi }}_2}( {{{\bf X}}( {{t_M}} )} )}\\ \vdots &\,\, \vdots &\,\, \vdots &\,\, \vdots \\ {{{{\boldsymbol \Psi }}_L}( {{{\bf X}}( {{t_1}} )} )}&\,\,{{{{\boldsymbol \Psi }}_L}( {{{\bf X}}( {{t_2}} )} )}&\,\, \cdots &\,\,{{{{\boldsymbol \Psi }}_L}( {{{\bf X}}( {{t_M}} )} )} \end{array}} \right]}\nonumber\\ =\left[ {\begin{array}{@{}*{4}{c}@{}} {{x_k}( {{t_1}} )}&\,\,{{x_k}( {{t_2}} )}&\,\, \cdots &\,\,{{x_k}( {{t_M}} )}\\ {{x_k}( {{t_2}} )}&\,\,{{x_k}( {{t_3}} )}&\,\, \cdots \,\,{{x_k}( {{t_{M + 1}}} )}\\ \vdots &\,\, \vdots &\,\, \vdots\,\,\,\, \vdots \\ {{x_k}( {{t_L}} )}&\,\,{{x_k}( {{t_{1 + L}}} )}\,\, \cdots &\,\,{{x_k}( {{t_{M + L - 1}}} )} \end{array}} \right].\nonumber\\ \end{eqnarray*}

Clearly, if we can construct the map ${{\boldsymbol \Psi }}$ or solve Equation (1) based on the measured *M* time points, the future values of the target variable can be obtained. The right-hand side of Equation (1) is the spatial information over multivariables X, whereas the left-hand side is the temporal information of a single target variable ${x_k}$. Hence, Equation (1) can be viewed as the transformation from spatial to temporal information, i.e. the STI equation.

Moreover, to make full use of the information of high-dimensional variables and also reduce the influence of noise in data, we randomly choose ***S*** variables among all *n* variables to solve Equation (1). Specifically, instead of ${{\bf X}}( {{t_m}} ),\ $ we first construct a nondelay attractor $\boldsymbol{\mathcal{S}}$ as ${{\boldsymbol{X}}_{\!\boldsymbol{\mathcal{S}}}}( {{t_m}} ) = [ {{x_i}( {{t_m}} ),\ {x_j}( {{t_m}} ), \ldots ,{x_s}( {{t_m}} )} ]$ for $m = 1,2, \ldots ,\ M$ by randomly choosing ***S*** variables (Fig. 1a) and then solve Equation (1) by replacing ${{{\bf X}}_\mathcal{S}}( {{t_m}} )$ to ${{\bf X}}( {{t_m}} )\ $in the left-hand side of Equation (1). Clearly, there are in total $_{s}^{n}$ = $\frac{{n!}}{{S!( {n - S})!}}$ such sampled attractors, which is a huge number for a high-dimensional system $(n > S > 1)$ and can be used for learning the map ${{\boldsymbol \Psi }}$ even from a short-term series. Based on the generalized embedding theory [22–24], each of these sampled nondelay attractors is topologically conjugated with the observed attractor if *S* > $2{d_O}$, where ${d_O}$ is the box-counting dimension of the original attractor, and therefore topologically conjugated with the delay attractor as well (see Section 1.1–1.2 in the Supplementary Materials). Specifically, as shown in Fig. 1a for each index tuple ${S_l} = \ ( {i,j, \ldots ,s} )$, a submap of ${{\boldsymbol \Psi }}$, denoted by ${{{\boldsymbol \Psi }}^{{S_l}}}$, between a sampled nondelay attractor and the delay attractor of the target variable ${x_k}$ is in the form of ${{{\boldsymbol \Psi }}^{{S_l}}}:{\boldsymbol{\ }}{{{\bf X}}_S}( {{t_m}} ) \to {\boldsymbol{Z}}( {{t_m}} )$ for $m\ = \ 1,2, \ldots ,\ M.$ Thus, by repeatedly solving Equation (1) between the delay attractor and each of those sampled nondelay attractors, we can have a large number of the predicted values of the target variable at each time point, which actually forms a distribution of each predicted value [39], thus not only fully exploiting the information of high-dimensional data, but also suppressing the data-noise effect (see Section 1 of the Supplementary Materials). Next, we design a neural network to learn this map ${{\boldsymbol \Psi }}\ $by considering all of those attractors as the training samples.

### AL neural network for constructing an attractor map

To construct the map ${{\boldsymbol \Psi }}$, we exploit the information contained in each ${{{\boldsymbol \Psi }}^{{S_l}}}$ as follows (Fig. 1a):

$$\begin{eqnarray*}{{\boldsymbol \Psi }} ( {{{\bf X}}( {{t_m}} )} ) &=& {\rm{\ }}{{\bf f}} ( {{{\boldsymbol \Psi }}^{{S_1}}}( {{{{\bf X}}_{{S_1}}}( {{t_m}} )} ),\nonumber\\
&&{{{\boldsymbol \Psi }}^{{S_2}}}({{{{\bf X}}_{{S_2}}}( {{t_m}} )} ), \ldots,\nonumber\\
&&{{{\boldsymbol \Psi }}^{{S_r}}}({{{{\bf X}}_{{S_r}}}( {{t_m}} )} ) ) = {\rm{\ }}{{\bf Z}}( {{t_m}} ),
\end{eqnarray*}$$

where **f** is a smooth function combining all sub-maps ${{{\boldsymbol \Psi }}^{{S_l}}}.$

${{\boldsymbol \Psi }}$ can be also decomposed into a set of injective functions, i.e. ${{\boldsymbol \Psi }}\ = \ \{ {{{\boldsymbol \Psi }}_1},{\boldsymbol{\ }} \ldots ,{\boldsymbol{\ }}{{{\boldsymbol \Psi }}_L}\} $ in which ${{{\boldsymbol \Psi }}_i}( {{{\bf X}}( {{t_m}} )} ) = {x_k} ( {{t_{m + i - 1}}} )$ stands for different time-delay prediction except ${{{\boldsymbol \Psi }}_1}$, which maps ${{\bf X}}( {{t_m}} )\ $to ${x_k}( {{t_m}} )$ (see Equation (1)). Thus, the learning of ${{\boldsymbol \Psi }}$ can be accomplished by simultaneously solving all ${{{\boldsymbol \Psi }}_i}$ in Equation (1) (see Section 1.4 in the Supplementary Materials). The whole procedure is illustrated at the bottom of Fig. 1a or Equation (1) by replacing ${{\bf X}}( {{t_m}} )$ with ${{{\bf X}}_S}( {{t_m}} )$ for each sampled nondelay attractor (see Section 1.4 in the Supplementary Materials). The left-hand side of the equation refers to the spatial information of the high-dimensional variables and the right-hand side of Equation (1) (or Fig. 1a) refers to the temporal information of ${x_k}$, where the shaded ones denote the unknown future to be predicted. From the viewpoint of time series analysis, the attractors map is to transform the spatial information among the observed high-dimensional variables into the temporal information of the target variable (Supplementary Fig. 1).

Provided with the observed high-dimensional data, plenty of possible sampled attractors can be reconstructed with different index tuple ${S_l} = \ ( {i,\ j,\ \ldots ,\ l} )$, the number of which grows combinatorically as the dimension of the system increases, actually $\frac{{n!}}{{S!( {n - S} )!}}$ in terms of the number of the sampled nondelay attractors and $\frac{{Mn!}}{{S!( {n - S} )!}}$ in terms of the number of the training samples. For each sampled attractor, one can obtain the corresponding predictor ${{{\boldsymbol \Psi }}^{{S_l}}}$ of the target variable ${x_k}$, which makes predictions in a different way. Therefore, by integrating the information contained in different sampled nondelay attractors, we can find ${{\boldsymbol \Psi }}$ in an accurate and comprehensive manner, which, in particular, is robust to noise in the data.

Here, we design a neural network to represent map ${{\boldsymbol \Psi }}$, called the AL neural network, in which the map ${{\boldsymbol \Psi }}$ can be naturally learned by using the Dropout [25] scheme as well as our consistent-training scheme (see Section 1.4.2 in the Supplementary Materials). Specifically, as shown in Fig. 1b, the AL neural network is a multi-layer neural network, where high-dimensional variables are taken as input neurons ${{\bf X}}( {{t_m}} )\ $(a nondelay attractor) but a target variable is taken as output neurons ${{\bf Z}}( {{t_m}} )$ (a delay attractor). Thus, the neural network represents the ${{\boldsymbol \Psi }}$ of Equation (1) that maps the nondelay attractors to the delay attractor. In particular, we adopt the Dropout [25] scheme to train the neural network, where we could drop each input unit/variable with a probability of $p$ (a pre-set number) in each epoch. As a result, different subsets of input (each subset represents an index tuple ${S_l}$) are fed to the neural network in different epochs. The total number of randomly sampled processes is equal to the number of epochs and the whole training process can be considered as averaging the information contained in different sampled nondelay attractors. From the fact that each sampled nondelay attractor preserves the dynamical information of the entire system in a different way, by integrating the information contained in these sampled nondelay attractors ${S_l}$, even under noise deterioration, we could learn a unified map ${{\boldsymbol \Psi }}\ = \ {{\bf f}}( {{{{\boldsymbol \Psi }}^{{S_1}}},\ {{{\boldsymbol \Psi }}^{{S_2}}},\ \ldots ,\ {{{\boldsymbol \Psi }}^{{S_r}}}} )$ (see Fig. 1b) rather than individual ${{{\boldsymbol \Psi }}^{{S_i}}}$ for $i = 1,2,\ldots, r$ (Fig. 1a), thus making accurate predictions of the future evolution of the system.

### ALM and multistep-ahead prediction by consistent-training scheme

Since ${{\boldsymbol \Psi }}$ is composed of the set of injective functions $\{ {{{{\boldsymbol \Psi }}_1},{\boldsymbol{\ }} \ldots ,{\boldsymbol{\ }}{{{\boldsymbol \Psi }}_L}} \}$, it simultaneously satisfies Equation (1). The previous RDE framework [21,39] trains different ${{{\boldsymbol \Psi }}_i}$ separately for one-step-ahead prediction and thus it may only capture a specific part of the system's dynamics, ignoring the global property. Therefore, as shown in Fig. 1b, we train ${{{\boldsymbol \Psi }}_1},\ {{{\boldsymbol \Psi }}_2},\ \ldots ,\ {{{\boldsymbol \Psi }}_L}$ simultaneously by considering the constraints ${{{\boldsymbol \Psi }}_i} ( {{{\bf X}}( {{t_j}} )} ) = {{{\boldsymbol \Psi }}_{i - 1}} ( {{{\bf X}}( {{t_{j + 1}}} )} )\ $of Equation (1). Therefore, we call such a learning procedure the consistent-training scheme, which is a cross-sample-training process. Specifically, when predicting the value of ${x_k}$ at ${t_m}$, ${{{\boldsymbol \Psi }}_1} ( {{{\bf X}}( {{t_m}} )} ) = {{{\boldsymbol \Psi }}_2} ( {{{\bf X}}( {{t_{m - 1}}} )} ) = \ldots = {{{\boldsymbol \Psi }}_L} ( {{{\bf X}}( {{t_{m - L + 1}}} )} )$ should be satisfied. Thus, during such a training process, the parameters of ${{{\boldsymbol \Psi }}_1},\ {{{\boldsymbol \Psi }}_2},\ \ldots ,\ {{{\boldsymbol \Psi }}_L}$ are updated simultaneously, as shown in Fig. 1b, thereby achieving multistep-ahead predictions. By doing so, each ${{{\boldsymbol \Psi }}_i}$ is forced to not only focus on a specific part of the system's dynamics, but also consider the global property (see Section 1.4.2 in the Supplementary Materials). Thus, a unified or consistent map ${{\boldsymbol \Psi }}$ can be obtained. Actually, to enhance the computational efficiency and robustness, we adopt a two-phase training process to learn the unified ${{\boldsymbol \Psi }}$, i.e. the pairwise-training scheme and consistent-training scheme, which are given in detail in Section 1.4.2 in the Supplementary Materials.

### Conditions for prediction from short-term data or a small number of samples

ALM can theoretically and numerically transform high-dimensional (spatial) information into the dynamical (temporal) information of one target variable, thus accurately predicting future values of the target variable even with short-term data. But, based on the theoretical derivation of ALM, the following conditions for a generic system are naturally required:

- the measured time series of the dynamical system are constrained in a low-dimensional attractor;
- all variables used for prediction are from the same dynamical system;
- stochasticity or noise in the measured data is small;
- high-dimensional variables are measured;
- the dynamical system is time-invariant during a short-term period.

For (i), even if a system is of high dimension, its attractor is of low dimension, which is generally satisfied in a real-world (dissipative) system. Actually, it is found that ALM also works well for the predictions of transient states. For (iii), a highly random system is unpredictable; in other words, there should be certain deterministic rules in the predicable systems. The conditions of (ii) and (iv) are obvious. For (v), a complex system is usually time-varying and thus difficult to be predicted even with long-term data, but it can be considered time-invariant only in the short term, i.e. the map ${{\boldsymbol \Psi }}$ of Equation (1) is time-invariant only during a short term, thus the prediction can be achieved by ALM but based on high-dimensional information. Clearly, all of those conditions are approximately satisfied for various real systems. It is noteworthy that the hyper-parameters setting remains sensitive in some of the experiments under the current framework. This is mainly due to the strong nonlinearity or/and stochasticity of the dynamical systems also with the observed noisy data, and thus how to make more in-depth theoretical analysis and further develop an appropriate framework taking these issues into consideration is an open and interesting problem for the future.

## Supplementary Material

nwaa025_Supplemental_File
