# Supplementary material for: Predicting future dynamics from short-term time series using an Anticipated Learning Machine
Source: Natl Sci Rev. 2020 Feb 19;7(6):1079–91. doi: 10.1093/nsr/nwaa025 (PMC8288952; doi:10.1093/nsr/nwaa025)
Supplement: nwaa025_Supplemental_File [file nwaa025_supplemental_file.docx]

Supplementary Materials for

Predicting Future Evolution from Short-term Time Series by Anticipated Learning Machine

Chuan Chen^1^, Rui Li^1^, Lin Shu^1^, Zhiyu He^1^, Jining Wang^1^, Chengming Zhang^2^, Huanfei Ma^3^, Kazuyuki Aihara^4,5^, Luonan Chen^2,6,7,8^*

Correspondence to: [lnchen@sibs.ac.cn](mailto:lnchen@sibs.ac.cn)

**This PDF file includes:**

Materials and Methods

Supplementary Text

Figs. S1 to S31

Tables S1 to S9

Content

[1. Anticipated Learning Machine Framework 3](#_Toc28090666)

[1.1 Discrete-time Dynamical Systems and Delay Embedding Theorem 3](#_Toc28090667)

[1.2 One-to-one Map between Delay Attractor and Observed Nondelay Attractor 4](#_Toc28090668)

[1.3 One-step-ahead Prediction 6](#_Toc28090669)

[1.4 Anticipated Neural Network to Represent Map between Delay Attractor and Nondelay Attractor 8](#_Toc28090670)

[1.4.1 Anticipated Neural Network Designing 8](#_Toc28090671)

[1.4.2 Anticipated Neural Network with Consistent-Training Scheme 9](#_Toc28090672)

[1.5 Anticipated Learning Machine with Dropout Scheme 12](#_Toc28090673)

[1.5.1 Randomly Sampling by Replacing $\boldsymbol{\Psi(X(}\boldsymbol{t}_{\boldsymbol{m}}))$ with $\boldsymbol{\Psi}^{\boldsymbol{S}}\mathbf{(X(}\boldsymbol{t}_{\boldsymbol{m}}\boldsymbol{))}$ 13](#_Toc28090674)

[2. Datasets 15](#_Toc28090675)

[2.1 Coupled Time-Variant Lorentz System 15](#_Toc28090676)

[2.2 Gene Dataset of Rats 16](#_Toc28090677)

[2.3 Plankton Dataset 16](#_Toc28090678)

[2.4 Ground Ozone Level Dataset 17](#_Toc28090679)

[2.5 Wind Dataset 17](#_Toc28090680)

[2.6 Stock Index Dataset 17](#_Toc28090681)

[2.7 Traffic Dataset 18](#_Toc28090682)

[2.8 Satellite cloud image Dataset 18](#_Toc28090683)

[3. Experiment Results 18](#_Toc28090684)

[3.1 Comparison Methods 18](#_Toc28090685)

[3.2 Results on Each Datasets 21](#_Toc28090686)

[**3.2.1** **Noise Free Coupled Lorentz System** 21](#_Toc28090687)

[**3.2.2** **Coupled Time-variant Lorentz System with Noise** 25](#_Toc28090688)

[**3.2.3** **Gene Dataset of Rats** 28](#_Toc28090689)

[**3.2.4** **Plankton Dataset** 36](#_Toc28090690)

[**3.2.5** **Ground Ozone Level Dataset** 37](#_Toc28090691)

[**3.2.6** **Wind Speed Dataset** 39](#_Toc28090692)

[**3.2.7** **Stock Index Dataset** 39](#_Toc28090693)

[**3.2.8** **Traffic Dataset** 40](#_Toc28090694)

[**3.2.9** **Satellite cloud image Dataset** 48](#_Toc28090695)

**Materials and Methods:**

1. Anticipated Learning Machine Framework

1.1 Discrete-time Dynamical Systems and Delay Embedding Theorem

For a general discrete-time (dissipative) system, the dynamics can be defined as

$\mathbf{X}\boldsymbol{(}\boldsymbol{t}_{\boldsymbol{m+1}}\boldsymbol{)= \phi(}\mathbf{X}\left( \boldsymbol{t}_{\boldsymbol{m}} \right)\boldsymbol{)}$,

where $\boldsymbol{\phi:}\mathbb{R}^{\boldsymbol{n}}\boldsymbol{\to}\mathbb{R}^{\boldsymbol{n}}$ is a nonlinear map, and its variables are defined in the *n*-dimensional state space$\mathbf{X}\left( \boldsymbol{t}_{\boldsymbol{m}} \right)\boldsymbol{=}\mathbf{[}\boldsymbol{x}_{\boldsymbol{1}}\left( \boldsymbol{t}_{\boldsymbol{m}} \right)\boldsymbol{,}\boldsymbol{x}_{\boldsymbol{2}}\left( \boldsymbol{t}_{\boldsymbol{m}} \right)\boldsymbol{,\ldots,}\boldsymbol{x}_{\boldsymbol{n}}\boldsymbol{(}\boldsymbol{t}_{\boldsymbol{m}}\boldsymbol{)}\mathbf{]∊}\mathbb{R}^{\boldsymbol{n}}$ at the *m-th* time point $\boldsymbol{t}_{\boldsymbol{m}}$, i.e. $\boldsymbol{t}_{\boldsymbol{m}}\boldsymbol{=t+m\tau}$ with a positive interval $\boldsymbol{\tau}$. After a sufficient time, all of states are converged into a *d*-dimensional compact manifold $\mathcal{V}$. Denoting the attractor contained in manifold $\mathcal{V}$ as $\mathcal{A}$ with the box-counting dimension $\boldsymbol{d}_{\boldsymbol{A}}$, the delay embedding theorem indicates that only using observed long-term data of a single variable can topologically reconstruct the attractor $\mathcal{A}$ of the original high-dimensional system when certain conditions are satisfied. We introduce the Takens’ embedding theorem ^1^ first.

***Theorem 1.*** Let $\mathcal{V}$ be a compact manifold of dimension $d$*.* For pairs $\left( \phi,h \right), \phi:\mathcal{V}\to\mathcal{V}$ a smooth (at least $\mathbb{C}^{2}$) diffeomorphism and $h:\mathcal{V}\mathbb{\to R}$ a smooth (at least $\mathbb{C}^{2}$) function, it is a generic property that the map $\psi_{\phi,h}:\mathcal{V}\to\mathbb{R}^{L},$ defined by

$$\psi_{\phi,h}\left( \mathbf{X} \right)=\left[ h\left( \mathbf{X} \right), h\left( \phi\left( \mathbf{X} \right) \right),\ldots,h\left( \phi^{L-1}\left( \mathbf{X} \right) \right) \right]$$

is an embedding when the integer $L>2d$.

The embedding theorem could also be stated in other forms, such as generalized embedding theorem ^2^ .

***Theorem 2***. Consider a compact, $d$-dimensional manifold $\mathcal{V}$ and a set of $L$ observation functions $\{h_{1},\ldots,h_{L}\}$ where $h_{i}: \mathcal{V}\mathbb{\to R}$ smoothly (at least $\mathbb{C}^{2}$). It is a generic property of all possible $\left\{ h_{i} \right\}$ that the map $\boldsymbol{\Psi}_{\{h_{i}\}}:\mathcal{V}\to\mathbb{R}^{L}$, i.e.

$$\boldsymbol{\Psi}_{\{h_{i}\}}=\left[ h_{1}\left( \mathbf{X} \right), h_{2}\left( \mathbf{X} \right),\ldots,h_{L}\left( \mathbf{X} \right) \right]$$

is an embedding when the integer $L>2d$.

It is clear that manifold $\mathcal{V}$’s dimension is usually much larger than attractor $\mathcal{A}$’s essential dimension, which limits the application scope of the embedding theorem. Thus, Sauer *et al.* generalized the embedding theorem for fractal dimension^3^.

***Remark***. Let $d_{A}$ denote the box-counting dimension of attractor $\mathcal{A}$, and $d$ denote the dimension of compact manifold $\mathcal{V}$. Since $d_{A}$ is usually smaller than $d$, for a set of smooth observation functions $\{h_{1},h_{2},\ldots,h_{L}\}$, the maps defined in Theorem 1 or Theorem 2 are still one-to-one on $\mathcal{A}$ and immersions on each compact subset of a smooth manifold contained in $\mathcal{A}$ as long as $L>2d_{A}$.

Note that provided that $L >2d_{A}$, we can topologically reconstruct the dynamics or attractor of the original high-dimensional system by using the observed data of one single variable. In addition, the conditions of the two theorems are sufficient conditions. For instance, even if $L <2d_{A}$ or the system is not in an attractor (i.e. not in a steady state but in a transient state), the dynamics may be reconstructed. Actually, it has been shown^24^ that *L* may be as low as $L\geq d_{A}+1$ Moreover, Robinson^4^ extends the embedding theorem to infinite-dimensional systems that have finite-dimensional attractors (i.e. $d_{A}$ is a finite number).

1.2 One-to-one Map between Delay Attractor and Observed Nondelay Attractor

The original delay embedding theorem addressed the question “Can we topologically reconstruct the dynamics of the original system by only using the long-term data of a single observed variable?” In this work, we will address another question “Can we predict future evolution of a single target variable by using the observed high-dimensional but short-term data?”

We now first define the first map related to such a time series prediction task.

***Definition 1.*** Let $\mathcal{O}$ denote the observed attractor of the system, and$d_{O}$ denotes the box-counting dimension of attractor $\mathcal{O}.$ $\mathbf{X}\left( t_{m} \right)=[x_{1}\left( t_{m} \right), x_{2}\left( t_{m} \right),\ldots, x_{n}(t_{m})]$ $∊\mathbb{R}^{n}$denotes the state at point $t_{m}$ on $\mathcal{O}$ where $x_{i}\left( t_{m} \right)$is the *i-th* component at the *m-th* time point. Then the delay coordinate map $\boldsymbol{\Psi} :\mathbb{R}^{n}\to\mathbb{R}^{L}$ is defined as

$$\boldsymbol{\Psi}\left( \mathbf{X}\left( t_{m} \right) \right)=\left[ x_{k}\left( t_{m} \right), x_{k}\left( t_{m+1} \right),\ldots,x_{k}\left( t_{m+L-1} \right) \right]=\mathbf{Z}\left( t_{m} \right),$$

where $x_{k}$ is a target variable of the system, the positive number $L>2d_{O},$ and $\mathbf{Z}\left( t_{m} \right)=\left[ x_{k}\left( t_{m} \right), x_{k}\left( t_{m+1} \right),\ldots,x_{k}\left( t_{m+L-1} \right) \right]∊\mathbb{R}^{L}$, for $m=1,2,\ldots$*.*

Thus, given multidimensional observed time-series, we have the corresponding observed attractor $\mathcal{O}$ with the state $\mathbf{X}\left( t_{m} \right)=[x_{1}\left( t_{m} \right), x_{2}\left( t_{m} \right),\ldots, x_{n}(t_{m})]$ for $m=1,2,\ldots$*,* which is a **nondelay attractor**. Here, each attractor is numerically represented by a series of data points. On the other hand, denote the attractor obtained from the delay coordinate map $\Phi_{D}$ as **delay attractor** $\mathcal{D}$ of a target variable $x_{k}$ with the state $\mathbf{Z}\left( t_{m} \right)=\left[ x_{k}\left( t_{m} \right), x_{k}\left( t_{m+1} \right),\ldots,x_{k}\left( t_{m+L-1} \right) \right]$ for $m=1,2,\ldots$. According to the generalized embedding theory, the observed attractor $\mathcal{O}$ is topological conjugate with the delay attractor $\mathcal{D}.$ Therefore, there exists a one-to-one map $\boldsymbol{\Psi}: \mathbb{R}^{n}\to\mathbb{R}^{L}.$ Note that $\boldsymbol{\Psi}$ is usually a function of time (in particular, for a complex system), but in the short-time time series, $\boldsymbol{\Psi}$ is considered as time-invariant. Next, we will show that once we find the map $\boldsymbol{\Psi}$, we can predict the future values of the target variable $x_{k}.$

Specifically, given an *n*-dimensional time series $\mathbf{X}\left( t_{m} \right)=[x_{1}\left( t_{m} \right), x_{2}\left( t_{m} \right),\ldots,x_{n}(t_{m})]$ with $M$ measured time points, $\boldsymbol{\Psi}$ maps each state of the observed attractor (nondelay attractor) into the state of the delay attractor of a target variable correspondingly (*5*), i.e. $\boldsymbol{\Psi}: \mathbf{X}\left( t_{m} \right)\to\mathbf{Z}\left( t_{m} \right)$ or

$\boldsymbol{\Psi}\left( \mathbf{X}\left( t_{m} \right) \right)=\mathbf{Z}\left( t_{m} \right)$ (S1)

for $m=1,2,\ldots,M$. Since $\mathbf{Z}\left( t_{m} \right)$ consists of $L$ entities which represent the value of $x_{k}$ at $L$ different time points, $\boldsymbol{\Psi}$ is composed of a set of injective functions $\{\boldsymbol{\Psi}_{1},\boldsymbol{\Psi}_{2},\ldots,\boldsymbol{\Psi}_{L}\}$ where

$$\boldsymbol{\Psi}_{i}\left( \mathbf{X}\left( t_{m} \right) \right)=x_{k}\left( t_{m+i-1} \right).$$

We can represent Eqn. (S1) as the following matrix form:

$$\left[ \begin{matrix} \boldsymbol{\Psi}_{1}\left( \mathbf{X}\left( t_{1} \right) \right) & \boldsymbol{\Psi}_{1}\left( \mathbf{X}\left( t_{2} \right) \right) & \boldsymbol{\Psi}_{1}\left( \mathbf{X}\left( t_{3} \right) \right) & \cdots& \boldsymbol{\Psi}_{1}\left( \mathbf{X}\left( t_{M-1} \right) \right) & \boldsymbol{\Psi}_{1}\left( \mathbf{X}\left( t_{M} \right) \right) \\ \boldsymbol{\Psi}_{2}\left( \mathbf{X}\left( t_{1} \right) \right) & \boldsymbol{\Psi}_{2}\left( \mathbf{X}\left( t_{2} \right) \right) & \boldsymbol{\Psi}_{2}\left( \mathbf{X}\left( t_{3} \right) \right) & \cdots& \boldsymbol{\Psi}_{2}\left( \mathbf{X}\left( t_{M-1} \right) \right) & \boldsymbol{\Psi}_{2}\left( \mathbf{X}\left( t_{M} \right) \right) \\ \boldsymbol{\Psi}_{3}\left( \mathbf{X}\left( t_{1} \right) \right) & \boldsymbol{\Psi}_{3}\left( \mathbf{X}\left( t_{2} \right) \right) & \boldsymbol{\Psi}_{3}\left( \mathbf{X}\left( t_{3} \right) \right) & \cdots& \boldsymbol{\Psi}_{3}\left( \mathbf{X}\left( t_{M-1} \right) \right) & \boldsymbol{\Psi}_{3}\left( \mathbf{X}\left( t_{M} \right) \right) \\ \boldsymbol{\Psi}_{4}\left( \mathbf{X}\left( t_{1} \right) \right) & \boldsymbol{\Psi}_{4}\left( \mathbf{X}\left( t_{2} \right) \right) & \boldsymbol{\Psi}_{4}\left( \mathbf{X}\left( t_{3} \right) \right) & \cdots& \boldsymbol{\Psi}_{4}\left( \mathbf{X}\left( t_{M-1} \right) \right) & \boldsymbol{\Psi}_{4}\left( \mathbf{X}\left( t_{M} \right) \right) \\ \vdots& \vdots& \vdots& \vdots& \vdots& \vdots\\ \boldsymbol{\Psi}_{L}\left( \mathbf{X}\left( t_{1} \right) \right) & \boldsymbol{\Psi}_{L}\left( \mathbf{X}\left( t_{2} \right) \right) & \boldsymbol{\Psi}_{L}\left( \mathbf{X}\left( t_{3} \right) \right) & \cdots& \boldsymbol{\Psi}_{L}\left( \mathbf{X}\left( t_{M-1} \right) \right) & \boldsymbol{\Psi}_{L}\left( \mathbf{X}\left( t_{M} \right) \right) \end{matrix} \right]$$

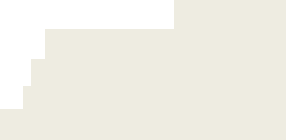
 =$\left[ \begin{matrix} x_{k}\left( t_{1} \right) & x_{k}\left( t_{2} \right) & x_{k}\left( t_{3} \right) & \cdots& x_{k}\left( t_{M-1} \right) & x_{k}\left( t_{M} \right) \\ x_{k}\left( t_{2} \right) & x_{k}\left( t_{3} \right) & x_{k}\left( t_{4} \right) & \cdots& x_{k}\left( t_{M} \right) & x_{k}\left( t_{M+1} \right) \\ x_{k}\left( t_{3} \right) & x_{k}\left( t_{4} \right) & x_{k}\left( t_{5} \right) & \cdots& x_{k}\left( t_{M+1} \right) & x_{k}\left( t_{M+2} \right) \\ x_{k}\left( t_{4} \right) & x_{k}\left( t_{5} \right) & x_{k}\left( t_{6} \right) & \cdots& x_{k}\left( t_{M+2} \right) & x_{k}\left( t_{M+3} \right) \\ \vdots& \vdots& \vdots& \vdots& \vdots& \vdots\\ x_{k}\left( t_{L} \right) & x_{k}\left( t_{1+L} \right) & x_{k}\left( t_{2+L} \right) & \cdots& x_{k}\left( t_{M+L-2} \right) & x_{k}\left( t_{M+L-1} \right) \end{matrix} \right]$. (S2)

where $\boldsymbol{\Psi}_{i}\left( \mathbf{X}\left( t_{j} \right) \right)=\boldsymbol{\Psi}_{i-1}\left( \mathbf{X}\left( t_{j+1} \right) \right)$. Clearly, in the right-hand-side of Eqn. (S2), $\left( x_{k}\left( t_{M+1} \right), x_{k}\left( t_{M+2} \right),\ldots,x_{k}\left( t_{M+L-1} \right) \right)$ are the unknown future values of the target variable $x_{k}$ beyond the time point *M*. Hence, if we can simultaneously solve Eqn. (S2) (all *M∙L* equations) based on the observed time-series, we can obtain the multi-step-ahead prediction of the target variable. In other words, we can transform the high-dimensional information of $\mathbf{X}$ to the future evolution of one target variable$x_{k}$. Note that the target variable can be any variable chosen from the high-dimensional system. Actually, it is not necessary to be a single target variable, and our theoretical method also holds for multiple target variables. The right-hand-side of Eqn.(S2) is the spatial information over multi-variables, whereas the left-hand-side is the temporal information of a single target variable. Thus, Eqn. (S2) can be viewed as the transformation from spatial to temporal information, i.e. the STI equation (Spatial-Temporal Information transformation equation).

On the other hand, from the embedding theorem, it is clear that we can also have the following map

$\boldsymbol{\Theta}: \mathbf{Z}\left( t_{m} \right)\to\mathbf{X}\left( t_{m} \right)$ for $m=1,2,\ldots,M,$

where $\boldsymbol{\Theta}: \mathbb{R}^{L}\to\mathbb{R}^{n}\mathbf{,}$and $\boldsymbol{\Theta=}\boldsymbol{\Psi}^{\mathbf{-}1}$.

Note that to have the one-to-one map, the following conditions are naturally required based on the theorems even if the system is high-dimensional and nonlinear.

1. The dynamics of the system is constrained to a low-dimension attractor.
2. All variables used in predictions are from the same system.
3. Stochasticity or noise is sufficient small.
4. The high-dimensional variables are measured.
5. The system is time-invariant or stationary during a short-term period.

Actually, all of the above conditions are approximately satisfied generally for a real system.

## 1.3 One-step-ahead Prediction

We first present a simple way to predict the target variable based on time-series data from Eqn. (S1), i.e. *one-step-ahead prediction*. Note that $x_{k}\left( t_{j} \right) \mathrm{for} j>M$ is unknown, and it is hard to find $\boldsymbol{\Psi}$ directly. Nevertheless, based on Eqn. (S1), $\boldsymbol{\Psi}$ is composed of a set of injective functions $\{\boldsymbol{\Psi}_{1},\boldsymbol{\Psi}_{2},\ldots,\boldsymbol{\Psi}_{L}\}$:

$$\boldsymbol{\Psi}_{i}\left( \mathbf{X}\left( t_{m} \right) \right)=x_{k}\left( t_{m+i-1} \right).$$

Then we find these injective functions one by one ($\boldsymbol{\Psi}_{1}$ is an known identical map, i.e. $\boldsymbol{\Psi}_{1}\left( \mathbf{X}\left( t_{m} \right) \right)=x_{k}\left( t_{m} \right)$ for $m=1,2,\ldots,M$). Specifically, given an $n$-dimensional time series $\mathbf{X}\left( t_{m} \right)=[x_{1}\left( t_{m} \right), x_{2}\left( t_{m} \right),\ldots,x_{n}(t_{m})]$ with $M$ measured time points, $\boldsymbol{\Psi}_{2}$ can be found by the following equations ($\boldsymbol{\Psi}_{2}$ maps second row of the left matrix of Eqn. (S2) into the corresponding row of the right matrix):

$$\left[ \begin{matrix} \boldsymbol{\Psi}_{2}\mathbf{(X}\left( t_{1} \right)) \\ \boldsymbol{\Psi}_{2}\mathbf{(X}\left( t_{2} \right)) \\ \vdots\\ \boldsymbol{\Psi}_{2}\mathbf{(X}\left( t_{M-2} \right)) \\ \boldsymbol{\Psi}_{2}\mathbf{(X}\left( t_{M-1} \right)) \end{matrix} \right]=\left[ \begin{matrix} x_{k}\left( t_{2} \right) \\ x_{k}\left( t_{3} \right) \\ \vdots\\ x_{k}(t_{M-1}) \\ x_{k}\left( t_{M} \right) \end{matrix} \right].$$

$\boldsymbol{\Psi}_{2}$ can be found by using basis function approximation, such as linear functions, polynomial functions or radial basis functions, or by using neural network training^5,19^. When $\boldsymbol{\Psi}_{2}$ is found, the prediction of $x_{k}(t_{M+1})$ can be made by $\tilde{x_{k}}\left( t_{M+1} \right)=\boldsymbol{\Psi}_{2}\left( \mathbf{X}\left( t_{M} \right) \right)\boldsymbol{,}$ which is one-step-ahead prediction of the target variable $x_{k}$. Then $\boldsymbol{\Psi}_{3}$ can be found by solving the following equations with $\tilde{x_{k}}\left( t_{M+1} \right)$ from $\boldsymbol{\Psi}_{2}$ ($\boldsymbol{\Psi}_{3}$ maps the third row of the left matrix of Eqn. (S2) into the corresponding row of the right matrix):

$$\left[ \begin{matrix} \boldsymbol{\Psi}_{3}\mathbf{(X}\left( t_{1}) \right) \\ \boldsymbol{\Psi}_{3}\mathbf{(X}\left( t_{2} \right)) \\ \vdots\\ \boldsymbol{\Psi}_{3}\mathbf{(X}\left( t_{M-2} \right)) \\ \boldsymbol{\Psi}_{3}\mathbf{(X}\left( t_{M-1}) \right) \end{matrix} \right]=\left[ \begin{matrix} x_{k}\left( t_{3} \right) \\ x_{k}\left( t_{4} \right) \\ \vdots\\ x_{k}(t_{M}) \\ \tilde{x_{k}}\left( t_{M+1} \right) \end{matrix} \right].$$

When $\boldsymbol{\Psi}_{3}$ is found, the prediction of $x_{k}(t_{M+2})$ can be made by $\tilde{x_{k}}\left( t_{M+2} \right)=\boldsymbol{\Psi}_{3}\left( \mathbf{X}\left( t_{M} \right) \right)$. Note that $\tilde{x_{k}}\left( t_{M+1} \right)$ is used as available data or label when training $\boldsymbol{\Psi}_{3}$. The rest $\boldsymbol{\Psi}_{i}$ can be trained in the similar way. Thus, we can separately solve each row of Eqn. (S2) or each component $\boldsymbol{\Psi}_{i}$ **of** $\boldsymbol{\Psi}$and have the prediction of the target variable $x_{k}$, but clearly the error is rapidly accumulated with the prediction of each step, in particular for the noisy data. Next, we design a neural network to solve Eqn. (S1) or Eqn. (S2) simultaneously by fully exploring observed high-dimensional data, rather than separately solving each component of the map.

## 1.4 Anticipated Neural Network to Represent Map between Delay Attractor and Nondelay Attractor

We can approximate $\boldsymbol{\Psi}$ of Eqn. (S1) or Eqn. (S2) separately by linear functions, polynomial functions or radial basis functions^5,19^, but it mainly works for the one-step-ahead prediction and there is a poor accuracy problem, in particular for the noisy data. Generally, $\boldsymbol{\Psi}$ is a nonlinear function, whose components are all linked together. Thus, if we solve Eqn. (S1) or Eqn. (S2) simultaneously for all components of the map by fully exploiting observed high-dimensional data as a system, we can significantly improve the robustness or the accuracy, meanwhile further predict the multi-step-ahead values of the target variable.

### 1.4.1 Anticipated Neural Network Designing

Here, we design a neural network to represent such a nonlinear map $\boldsymbol{\Psi}$, called the anticipated learning (AL) neural network, in which the map $\boldsymbol{\Psi}$ can be naturally learned by using the Dropout^6^ scheme as well as our consistent-training scheme. Specifically, as shown in Fig. 1(b), the AL neural network is a multi-layered neural network, where high-dimensional variables are taken as input neurons $\mathbf{X}(t_{m})$ (a nondelay attractor) but a target variable is taken as output neurons $\mathbf{Z}(t_{m})$ (a delay attractor). Thus, the neural network represents the $\boldsymbol{\Psi}$ of Eqn. (1) or Eqn. (S1) that maps the nondelay attractor to the delay attractor. Note that $\mathbf{X}\left( t_{m} \right)$ is a vector with multiple-variables at time$t_{m}$, while $\mathbf{Z}\left( t_{m} \right)$ is a vector with a single variable at multiple time points. Thus, as illustrated in Fig. S1, $\boldsymbol{\Psi}$ is to transform high-dimensional (spatial) information $\mathbf{X}\left( t_{m} \right)$to dynamical (temporal) information $\mathbf{Z}\left( t_{m} \right)$of a single variable.


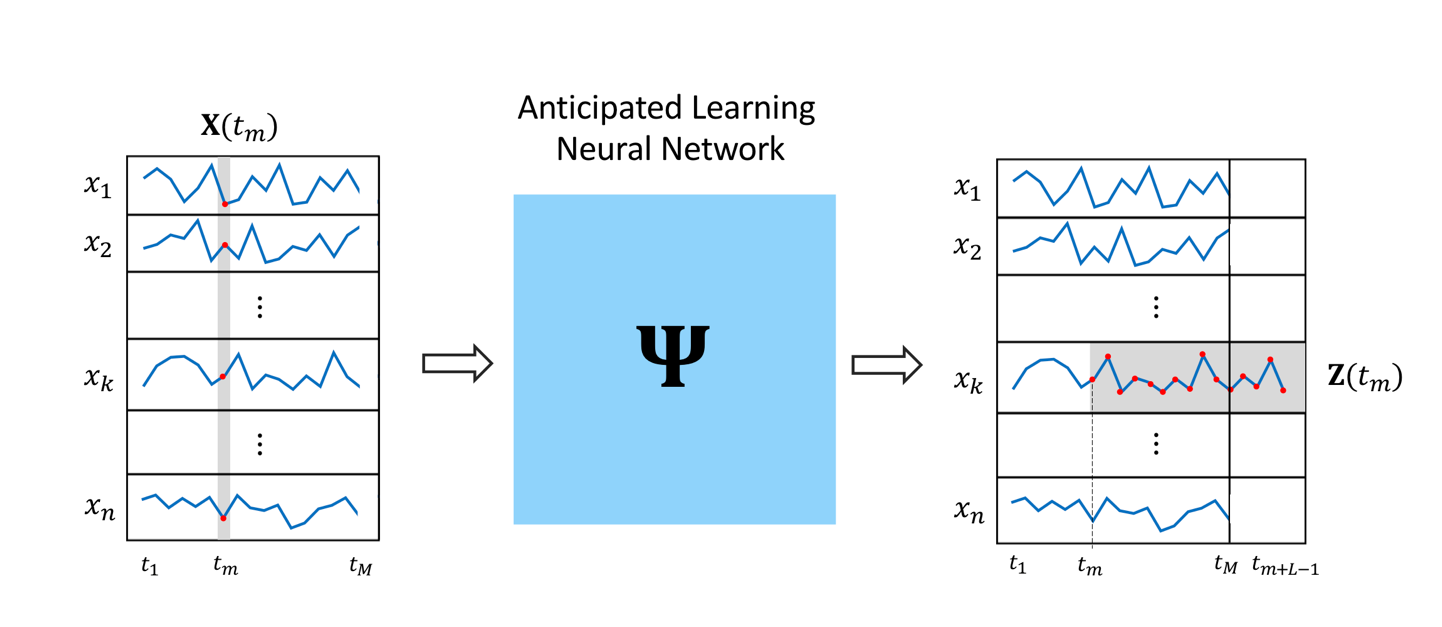


**Output**

**(temporal data)**

**Input**

**(spatial data)**

$$\boldsymbol{Z}\left( t_{m} \right)=\left[ x_{k}\left( t_{m} \right), x_{k}\left( t_{m+1} \right),\ldots,x_{k}\left( t_{m+L-1} \right) \right]$$

$$\boldsymbol{\Psi}\left( \boldsymbol{X}\left( t_{m} \right) \right)\mathbf{=}\boldsymbol{Z}\left( t_{m} \right)$$

$$\boldsymbol{X}\left( t_{m} \right)=[x_{1}\left( t_{m} \right), x_{2}\left( t_{m} \right),\ldots, x_{n}(t_{m})]$$

Fig. S1 Transformation from spatial information $\mathbf{X}\left( t_{m} \right)$to dynamical information $\mathbf{Z}\left( t_{m} \right)$ made by Anticipated Learning neural network $\boldsymbol{\Psi}$ at each point $t_{m}$ (see the shaded parts).

As shown in the left side (the shaded part) of Fig. S1, the input of the neural network is $\mathbf{X}\left( t_{m} \right)=[x_{1}\left( t_{m} \right), x_{2}\left( t_{m} \right),\ldots, x_{n}(t_{m})]$, while in the right side (the shaded part) of Fig. S1, the output is $\mathbf{Z}\left( t_{m} \right)=\left[ x_{k}\left( t_{m} \right), x_{k}\left( t_{m+1} \right),\ldots,x_{k}\left( t_{m+L-1} \right) \right]$, for $m=1,2,\ldots,M$.

Next section, we show how to efficiently train the AL neural network by adopting Dropout ^6^ scheme. Briefly speaking, we adopt the Dropout^6^ scheme to train the AL neural network, where we could drop each input unit/variable with a probability of $p$ (a pre-set number) in each epoch. As a result, different subsets of input (each subset represents an index tuple $S_{l}$ for a sampled nondelay attractor) are fed to the neural network in different epochs. The total number of randomly sampling processes is equal to the number of epochs, and the whole training process can be considered as averaging the information contained in different sampled nondelay attractors. From the fact that each sampled nondelay attractor preserves the dynamical information of the entire system in a different way, by integrating the information contained in these sampled nondelay attractors $S_{l}$, even under noise deterioration we could learn the consistent map $\boldsymbol{\Psi=f}\left( \boldsymbol{\Psi}^{S_{1}}\mathbf{,}\boldsymbol{\Psi}^{S_{2}}\boldsymbol{,\ldots,}\boldsymbol{\Psi}^{S_{r}} \right)$ efficiently, thus making accurate prediction of the future evolution of the system where $\mathbf{f}$ represents the information integrating function or a unified map over all individual $\boldsymbol{\Psi}^{S_{i}}$for ***i=1,2,..,r***.

### 1.4.2 Anticipated Neural Network with Consistent-Training Scheme

Since different $\boldsymbol{\Psi}_{i}$ makes different steps forward predictions, they capture the system’s dynamics at different perspectives. If we train different $\boldsymbol{\Psi}_{i}$ separately, it is possible that each $\boldsymbol{\Psi}_{i}$ will only focus on a specific part of the system’s dynamics and ignore the global property. Therefore, we can train $\boldsymbol{\Psi}_{1}, \boldsymbol{\Psi}_{2}, \ldots, \boldsymbol{\Psi}_{L}$ simultaneously to link all *L* components, thus making a unified or consistent $\boldsymbol{\Psi}$. Specifically, note that when predicting $x_{k}$’s value at $t_{j}$, the predictions made by different $\boldsymbol{\Psi}_{\boldsymbol{i}}$ should be the same, i.e., $\boldsymbol{\Psi}_{1}\left( \mathbf{X}\left( t_{j-1} \right) \right)=\boldsymbol{\Psi}_{2}\left( \mathbf{X}\left( t_{j-2} \right) \right)=\ldots=\boldsymbol{\Psi}_{j-1}\left( \mathbf{X}\left( t_{1} \right) \right)$. During training process, such consistent constraints are further added to update the parameters of $\boldsymbol{\Psi}_{1}, \boldsymbol{\Psi}_{2}, \ldots, \boldsymbol{\Psi}_{L}$ simultaneously, and thus all of the outputs are updated based on the following consistent training scheme

$\tilde{x_{k}}\left( t_{m} \right)=\sum_{j=1}^{L} w_{j1}\boldsymbol{\Psi}_{j}\left( \mathbf{X}\left( t_{m-j+1} \right) \right)$, $\sum_{j=1}^{L} w_{j1}$=1, $w_{j1}\geq0$,

$\tilde{x_{k}}\left( t_{m+1} \right)=\sum_{j=2}^{L} w_{j2}\boldsymbol{\Psi}_{j}\left( \mathbf{X}\left( t_{m-j+2} \right) \right)$, $\sum_{j=2}^{L} w_{j2}$=1, $w_{j2}\geq0$,

…

$\tilde{x_{k}}\left( t_{m+L-1} \right)=\sum_{j=L}^{L} w_{jL}\boldsymbol{\Psi}_{j}\left( \mathbf{X}\left( t_{m-j+L} \right) \right)$, $\sum_{j=L}^{L} w_{jL}$=1, $w_{jL}\geq0,$

which can be viewed as cross-sample training for the neural network due to the involvement of multiple samples. Here, the weight parameters $w_{ji}$can be chosen by various schemes, e.g.

1. Each weight $w_{ji} \mathrm{is}$ equal, i.e. $w_{ji}=\frac{1}{L-i+1}$ for $i=2, 3,\ldots,L; j=2,\ldots,L.$
2. Each weight$w_{ji}$is set based on the number of its input data, i.e. $w_{ji}=\frac{2M+2-2j}{\left( 2M+2-i-L \right)\left( L-i+1 \right)}$ for $i=2,3,\ldots,L; j=2,\ldots,L.$

After the training, we use the weighted sum of the predictions (multi-step-ahead predictions) made by those $\boldsymbol{\Psi}_{i}$ as a result, i.e.,

$\tilde{x_{k}}\left( t_{M+1} \right)=\sum_{j=2}^{L} w_{j2}\boldsymbol{\Psi}_{j}\left( \mathbf{X}\left( t_{M-j+2} \right) \right)$,

$\tilde{x_{k}}\left( t_{M+2} \right)=\sum_{j=3}^{L} w_{j3}\boldsymbol{\Psi}_{j}\left( \mathbf{X}\left( t_{M-j+3} \right) \right)$,

...

$\tilde{x_{k}}\left( t_{M+L-1} \right)=\sum_{j=L}^{L} w_{jL}\boldsymbol{\Psi}_{j}\left( \mathbf{X}\left( t_{M-j+L} \right) \right).$

In practice, the consistent-training scheme can be applied to connect an arbitrary number of adjacent ones instead of all *L* components together to initialize each $\boldsymbol{\Psi}_{j}$. However, to enhance the computational robustness and efficiency of the consistent-training, we adopt a two-phase training process to learn the unified $\boldsymbol{\Psi}$**,** i.e. (1) the pairwise-training scheme and (2) the consistent-training scheme.

**(1) Pairwise-training scheme.** As shown in Fig. S2, for example, we train two predictors $\boldsymbol{\Psi}_{j}$ and $\boldsymbol{\Psi}_{j+1}$ together (i.e. only link two components of the map) and use the mean (i.e. the equal weighting scheme) of different predictors as the final prediction. Specifically, when predicting $\tilde{x_{k}}\left( t_{m+1} \right)$, $\boldsymbol{\Psi}_{2}$ can be found by training

$$\left[ \begin{matrix} \boldsymbol{\Psi}_{2}\mathbf{(X}\left( t_{2}) \right) \\ \boldsymbol{\Psi}_{2(}\mathbf{X}\left( t_{3}) \right) \\ \vdots\\ \boldsymbol{\Psi}_{2}\mathbf{(X}(t_{M-2})) \\ \boldsymbol{\Psi}_{2}\mathbf{(X}\left( t_{M-1}) \right) \end{matrix} \right]=\left[ \begin{matrix} x_{k}\left( t_{3} \right) \\ x_{k}\left( t_{4} \right) \\ \vdots\\ x_{k}(t_{M-1}) \\ x_{k}\left( t_{M} \right) \end{matrix} \right],$$

and $\boldsymbol{\Psi}_{3}$ can be found by fitting

$$\left[ \begin{matrix} \boldsymbol{\Psi}_{3}\mathbf{(X}\left( t_{1} \right)) \\ \boldsymbol{\Psi}_{3}\mathbf{(X}\left( t_{2}) \right) \\ \vdots\\ \boldsymbol{\Psi}_{3}\mathbf{(X}(t_{M-1})) \\ \boldsymbol{\Psi}_{3}\mathbf{(X}\left( t_{M-2}) \right) \end{matrix} \right]=\left[ \begin{matrix} x_{k}\left( t_{3} \right) \\ x_{k}\left( t_{4} \right) \\ \vdots\\ x_{k}(t_{M-1}) \\ x_{k}\left( t_{M} \right) \end{matrix} \right].$$

$\boldsymbol{\Psi}_{2}$ and $\boldsymbol{\Psi}_{3}$ are trained at the same time and their predictions are constrained to be the same during training, i.e., $\boldsymbol{\Psi}_{2}\left( \mathbf{X}\left( t_{j-1} \right) \right)=\boldsymbol{\Psi}_{3}\left( \mathbf{X}\left( t_{j-2} \right) \right).$ When $\boldsymbol{\Psi}_{2}$ and $\boldsymbol{\Psi}_{3}$ are found, the prediction of $x_{k}(t_{m+1})$ can be made by $\tilde{x_{k}}\left( t_{m+1} \right)=\frac{\boldsymbol{\Psi}_{2}\left( \mathbf{X}\left( t_{m} \right) \right)\boldsymbol{+}\boldsymbol{\Psi}_{3}\left( \mathbf{X}\left( t_{m-1} \right) \right)}{2}$**,** based on equal weight parameters $w_{ji}=\frac{1}{2}$. This is used, in turn, to train the rest of predictors. Such a training scheme is called the pairwise-training scheme in this work.

**(2) Consistent-training scheme.** After learning $\boldsymbol{\Psi}_{1}, \boldsymbol{\Psi}_{2}, \ldots, \boldsymbol{\Psi}_{L}$ by the above pairwise- training scheme, we further adopt the following consistent-training scheme to consistently train those predictors, and the final results can be estimated by multi-step prediction with all of the learnt $\boldsymbol{\Psi}_{l}$.

During the consistent-training process, we set different inputs for different predictors respectively to ensure that they can generate the same output, and the training constraints are in the form of loops. For $\boldsymbol{\Psi}_{1}, \boldsymbol{\Psi}_{2}, \ldots, \boldsymbol{\Psi}_{L}$, we let

$\boldsymbol{\Psi}_{2}\left( \mathbf{X}\left( t_{j-2} \right) \right) = \boldsymbol{\Psi}_{1}\left( \mathbf{X}\left( t_{j-1} \right) \right)$,

$\boldsymbol{\Psi}_{3}\left( \mathbf{X}\left( t_{j-3} \right) \right) = \boldsymbol{(\Psi}_{1}\left( \mathbf{X}\left( t_{j-1} \right) \right)+\boldsymbol{\Psi}_{2}\left( \mathbf{X}\left( t_{j-2} \right) \right))/2$,

$$\ldots\ldots$$

$\boldsymbol{\Psi}_{L}\left( \mathbf{X}\left( t_{j-L} \right) \right) = \boldsymbol{(\Psi}_{1}\left( \mathbf{X}\left( t_{j-1} \right) \right)+\boldsymbol{\Psi}_{2}\left( \mathbf{X}\left( t_{j-2} \right) \right) +\ldots+\boldsymbol{\Psi}_{L-1}\left( \mathbf{X}\left( t_{j-L+1} \right) \right))/(L-1)$,

$$\boldsymbol{\Psi}_{1}\left( \mathbf{X}\left( t_{j-1} \right) \right) = \boldsymbol{(\Psi}_{2}\left( \mathbf{X}\left( t_{j-2} \right) \right)+\boldsymbol{\Psi}_{3}\left( \mathbf{X}\left( t_{j-3} \right) \right) +\ldots+ \boldsymbol{\Psi}_{L}\left( \mathbf{X}\left( t_{j-L} \right) \right))/(L-1)$$

in order for each loop, where $j=3,\ldots,M+1$. These training constraints are taken as part of the loss function, enforcing the left-hand-sides of the equations closed to the right-hand-sides. Such a scheme is the consistent-training scheme.

To make the multi-step predictions, all predictors will be used with the same inputs. For example, once $\tilde{x_{k}}\left( t_{M+1} \right)\ldots\tilde{x_{k}}\left( t_{M+L} \right)$ are needed, we use $\boldsymbol{\Psi}_{1}\left( \mathbf{X}\left( t_{M} \right) \right)\ldots\boldsymbol{\Psi}_{L}\left( \mathbf{X}\left( t_{M} \right) \right)$ to predict them. The above procedure is the two-phase training process to learn the unified $\Psi$.

In our experiment, we utilize the two-phase training process (i.e. first pairwise-training and then consistent-training) to learn the map/model. The prediction result shows that our design performs better than other architectures. Specifically, the ALM model can learn both local and global information on dynamics, which explains the superiority of ALM. Here, we should remark that the relation among $\boldsymbol{\Psi}_{l}$s is complex, for which the aforementioned algorithm or scheme is only one of the possible approaches for training the unified $\boldsymbol{\Psi}$ consistently with all $\boldsymbol{\Psi}_{l}$s for proper approximation. Theoretically, we can directly use the consistent-training scheme (without the pairwise-training scheme) to learn the unified map, but we computationally found that such results were not so robust, which implies that it is important to have appropriate initial values of the neural network (which are generated by the pairwise-training scheme in this work). To make the prediction robust, other more appropriate schemes taking the inherent complexity into consideration remain to be an open problem.

**
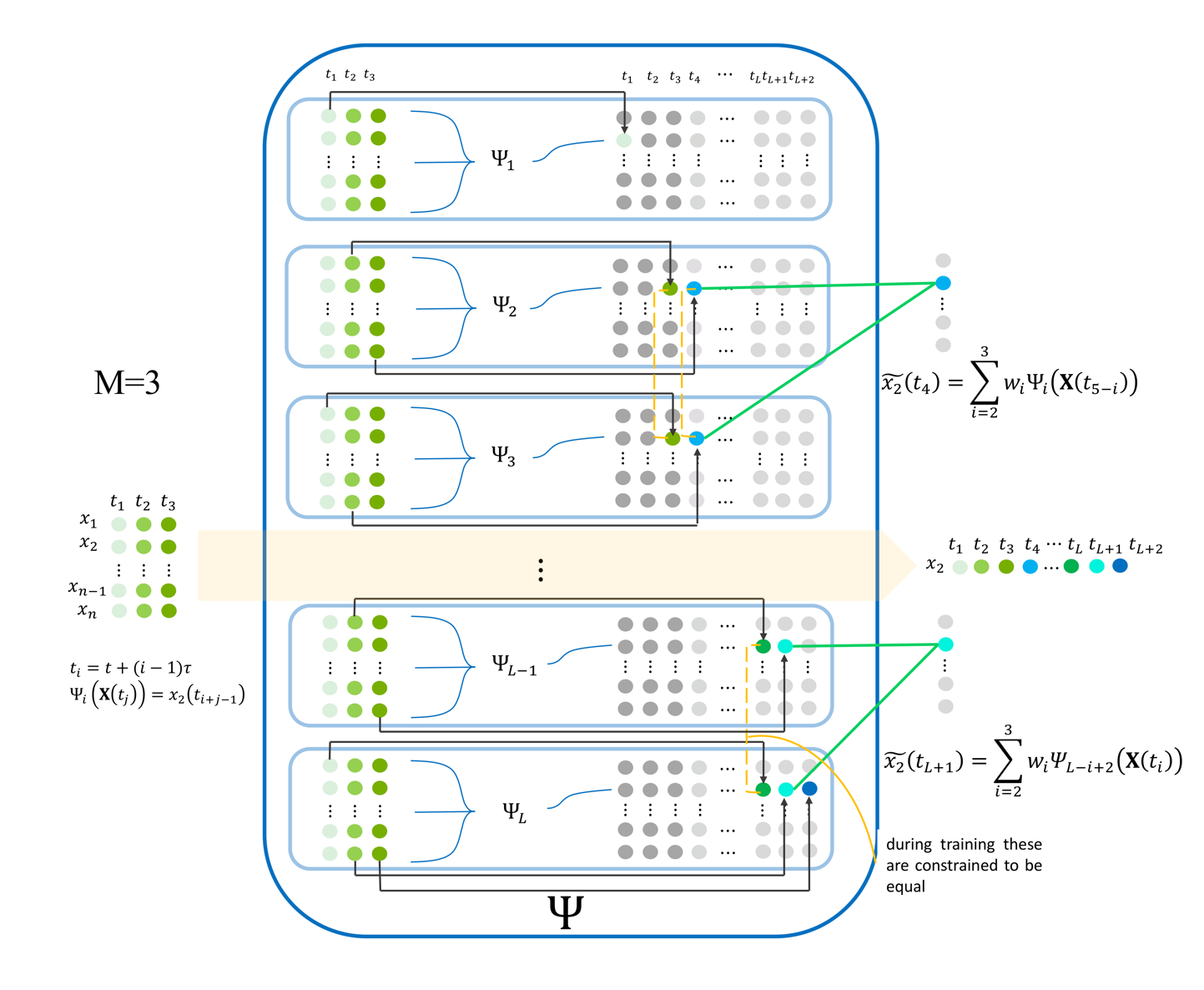
**

Fig. S2: Training process of ALM when M=3.

## 1.5 Anticipated Learning Machine with Dropout Scheme

To reduce the influence of noise in data and make full use of the intertwined interactions among variables, we find $\boldsymbol{\Psi}_{i}$ by randomly sampling or choosing partial variables among all *n* variables. We define the sample coordinate map first.

***Definition 2.*** The sample coordinate map $\Phi_{S}:\mathbb{R}^{n}\to\mathbb{R}^{S}$ is defined as

$$\Phi_{S}\left( \mathbf{X}\left( t_{m} \right) \right)=\boldsymbol{X}_{S}\left( t_{m} \right)=\left[ x_{i}\left( t_{m} \right), x_{j}\left( t_{m} \right),\ldots,x_{s}\left( t_{m} \right) \right],$$

where $x_{i}, x_{j},\ldots,x_{s}$ are *S* variables of the system with $S> 2d_{O}$ namely $\dim\left( \boldsymbol{X}_{S}\left( t_{m} \right) \right)>2d_{O}$.

### 1.5.1 Randomly Sampling by Replacing $\boldsymbol{\Psi}\left( \mathbf{X}\left( \boldsymbol{t}_{\boldsymbol{m}} \right) \right)$ with $\boldsymbol{\Psi}^{\boldsymbol{S}}\left( \mathbf{X}\left( \boldsymbol{t}_{\boldsymbol{m}} \right) \right)$

Given the observed attractor $\mathcal{O}$, denote $\dim\left( \boldsymbol{X}_{S}\left( t_{m} \right) \right)$ as $S$. That is, we randomly choose *S* variables from all the *n* variables $(n\geq S)$, and then use the data of only these *S* variables for the training, instead of all variables. Clearly, in such a way, we can construct $\left( \begin{matrix} n \\ S \end{matrix} \right)=\frac{n!}{S!\left( n-S \right)!}$ sampled attractors $\mathcal{S}$ by the sample coordinate map. These sampled attractors are all topologically conjugate with the observed attractor when $S> 2d_{O}$, and therefore topologically conjugate with the delay attractor as well. Thus, for each index tuple $S_{l}=(i,j,\ldots,s)$*,* a submap of $\boldsymbol{\Psi}$, denoted by $\boldsymbol{\Psi}^{S_{l}}$, can be obtained as a predictor for the target variable $x_{k}$ in the form of

$$\boldsymbol{\Psi}^{S_{l}}\left( \left[ x_{i}\left( t_{m} \right),x_{j}\left( t_{m} \right),\ldots x_{s}\left( t_{m} \right) \right] \right)={\boldsymbol{\Psi}^{S_{l}}\boldsymbol{(X}}_{S}\left( t_{m} \right))=\left[ x_{k}\left( t_{m} \right),x_{k}\left( t_{m+1} \right),\ldots x_{k}\left( t_{m+L-1} \right) \right]=\mathbf{Z}\left( t_{m} \right).$$

$\boldsymbol{\Psi}^{S_{l}}$ is composed of a set of injective functions $\{\boldsymbol{\Psi}_{1}^{S_{l}},\boldsymbol{\Psi}_{2}^{S_{l}},\ldots,\boldsymbol{\Psi}_{L}^{S_{l}}\}$ where $\boldsymbol{\Psi}_{j}^{S_{l}}\boldsymbol{:}\boldsymbol{X}_{S}\left( t_{i} \right)\to x_{k}(t_{i+j-1})$. Then using the information contained in $\boldsymbol{\Psi}_{j}^{S_{l}}$, we could find $\boldsymbol{\Psi}_{j}$. Specifically, instead of $\mathbf{X}\left( t_{m} \right),$we first construct a nondelay attractor $\mathcal{S}$ as $\boldsymbol{X}_{S}\left( t_{m} \right)=\left[ x_{i}\left( t_{m} \right), x_{j}\left( t_{m} \right),\ldots,x_{s}\left( t_{m} \right) \right]$ for $m=1,2,\ldots,M$ by randomly choosing *S* variables (Fig. 1(a)), and then solve Eqn. (S1) or (S2) by replacing $\mathbf{X}\left( t_{m} \right)$with $\mathbf{X}_{S}\left( t_{m} \right)$in the left-hand-side of Eqn. (S1) or (S2), which is the spatial-temporal information transformation (STI) equation^19^. Clearly, there are totally $\left( \begin{matrix} n \\ S \end{matrix} \right)=\frac{n!}{S!\left( n-S \right)!}$ such sampled attractors (total $M\left( \frac{n!}{S!\left( n-S \right)!} \right)$ samples), which is a huge number for a high-dimensional system $(n>S>1)$ and can be used for training the map $\boldsymbol{\Psi}$ even with a short-term series. For instance, for only the observed M=20 samples (small sample size) with n=10000 (number of the high-dimension variables) and S=100 (number of the randomly chosen variables), we can actually generate non-redundant 20$\left( \frac{10000!}{100!\left( 10000-100 \right)!} \right)$ >> 10^200^ training samples to train the map. Thus, by repeatedly solving Eqn. (S1) with the delay attractor and each of those sampled nondelay attractors, we can learn the map $\boldsymbol{\Psi}$ and meanwhile have the multi-step-ahead predictions, which not only fully exploits the information of high-dimensional data but also suppresses the data noise effect. Thus, $\boldsymbol{\Psi}$ can be found by using information contained in each $\boldsymbol{\Psi}^{S_{l}}$.

Specifically, we adopt the Dropout^6^ scheme to implement this process. Applying Dropout to the input layer is equal to sample a “thinned” neural network (the map from the sampled nondelay attractor to the delay attractor) from the original neural network (the map from the observe nondelay attractor to the delay attractor). In each epoch, a new “thinned” neural network is sampled and trained, and the whole training process can be considered as training a collection of thinned networks with extensive weight sharing. As shown in Fig. S3, the input $x_{4}$ is dropped in this epoch.


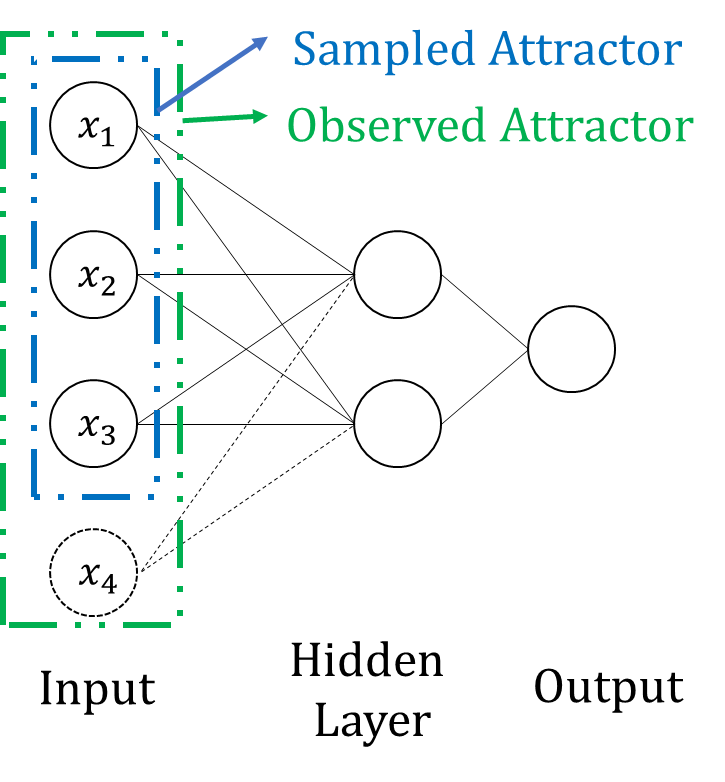


Fig. S3: Dropout as randomly sampling in the AL neural network. The green dash line represents the observed nondelay attractor and the blue dash line represents the sampled nondelay attractor.

In summary, the goal of ALM is to predict the unknown shaded values by solving Eqn. (S2). The algorithm of ALM is illustrated in Algorithm 1.


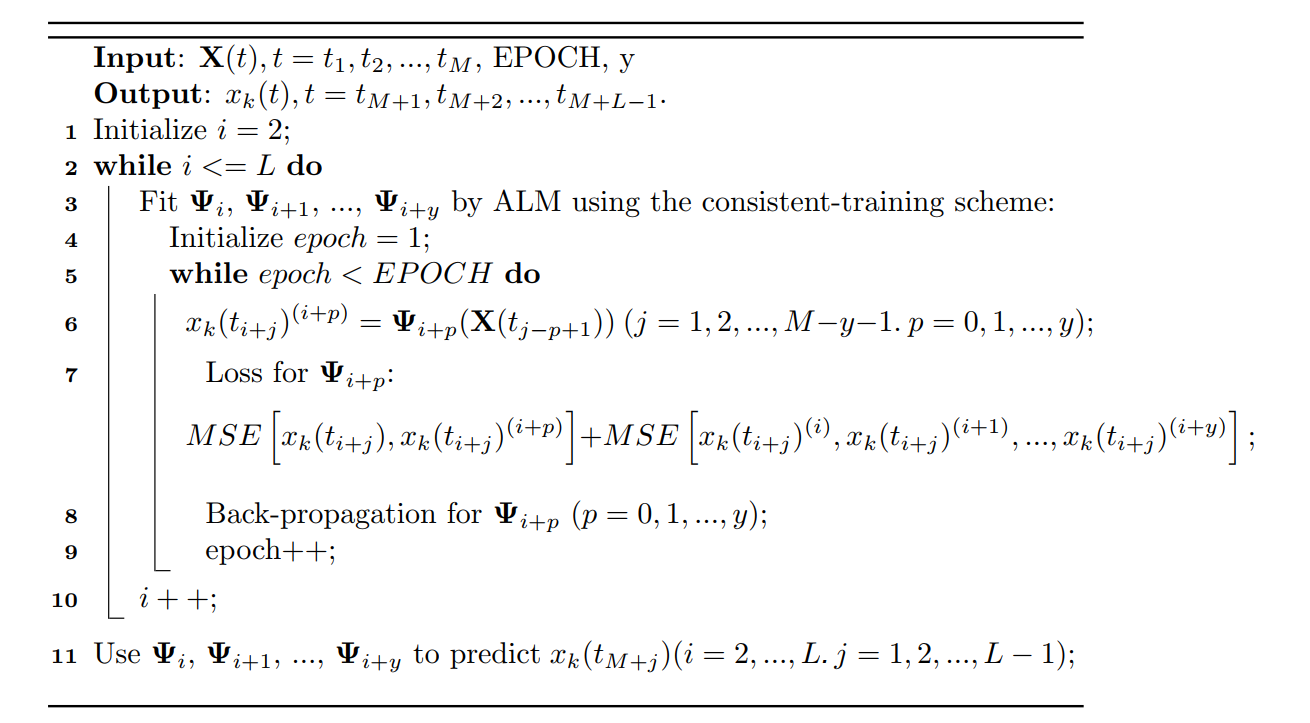


Algorithm 1: Algorithm of ALM, where MSE represents the mean squared error.

The main difference between ALM and the traditional neural network is that ALM seeks to find the dynamics of the system from high-dimensional information, while the traditional neural network seeks to find the historical statistics of the system from a large number of samples (or a long-term time series). By capturing the extrinsic dynamics of the system, ALM can not only make accurate predictions of time series, but also help users better understand the system’s property. In addition, with the use of the consistent-training scheme, ALM could learn the dynamics in a cross-sample manner, which improves ALM’s ability of capturing the global property of the system. In other words, for a high-dimensional system or complex system, by constraining the dynamics to a low-dimension space which is actually an inherent property of such a system, ALM can efficiently and robustly learn its dynamics even with a small number of samples, in contrast to the statistics-based machine learning which excavates the historical statistics of the system at a high-dimensional space and thus requires a large number of samples. Since the training-samples also include the unknown future values of the target variable because of delay embedding, it is semi-supervised or anticipated learning, which may be one key intelligence of the brain.

Here we should also mention that, the proposed ALM method is different from the traditional embedding based methods for time-series prediction which mainly project the data into a lower-dimensional space^19,20^, ALM can be regarded as difference equations or discretization of differential equations, whose states/variables are naturally constrained in a lower-dimensional dynamical trajectory.

1. **Datasets**

## 2.1 Coupled Time-Variant Lorentz System

To validate our model’s ability of capturing high-dimensional nonlinear system’s dynamics, we consider a 90D time-variant coupled Lorentz system. The *i-*th (*i*=1,2,…,30) coupled subsystem is given by

$$\dot{x_{i}}=\sigma(t)\left( y_{i}-x_{i} \right)+Cx_{i-1},$$

$$\dot{y_{i}}=\rho x_{i}-y_{i}-x_{i}z_{i},$$

$$\dot{z_{i}}=-\beta z_{i}+x_{i}y_{i}.$$

The coupling term $Cx_{i-1}$ means the *i-*th subsystem is coupled with the $\left( i-1 \right)$th subsystem via $x$ component. When $i=1$, we set $i-1$ as $30$ so that the system could be closed. We set $\rho, \beta$ and $C$ to be typical values, i.e., $\rho=28, \beta=\frac{8}{3}, C=0.1.$ $\sigma\left( t \right)=10+0.01(t |10)$ is a time-variant parameter function with its value being initially set to be 10 and increased by 0.01 after each ten time intervals, as a result, the system is time-switching.

When generating the dataset, we set the initial values to be 0.0001 and the time interval $\Delta t$ to be $0.02$. After transient dynamics we select 30 points as training data and then make the predictions. As shown in Fig. 2, ALM reconstructs the attractor accurately.

- 1. **Gene Dataset of Rats**

The construction of this dataset is based on gene expression profiles with Affymetrix microarray measured on the laboratory rat (Rattus norvegicus) cultured cells from SCN, which consists of the expression of 31099 genes of 23 time points from 0h to 90h^7^. At the time of 18h, the phase reset stimulus by drug forskolin was applied. We randomly select four genes related to circadian rhythm, which are Pfkfb1, Fut4, Pfkfb3 and Rorc, and four genes unrelated to circadian rhythm, which are LOC301113, Nr1h3, Spink1 and Trim17 to predict. We use the first 11 time points as the training data and make predictions on the next 11 time points.

Since the total 31099 genes are not necessarily sharing the same attractor, i.e., they are not necessarily intertwined. To make sure the one-to-one map exists, for each gene we use mutual information to select 4999 most related genes and form a new 5000-dimensional system for it. As shown in Fig. 3 and Figs. S10 –S17, compared with other methods, although ALM is slightly worse than a few methods on the prediction of some genes in numerical or trend errors, the composite indicators of ALM are always the highest. This indicates that ALM not only works well on genes relevant to circadian rhythm such as Fut4, but also on those irrelevant to circadian rhythm such as Spink1, which demonstrates ALM’s ability to transform information of the high-dimensional variables to the nonlinear evolution of any target variable.

- 1. **Plankton Dataset**

This dataset^8^ is collected from an optical plankton counter (OPC) and CTD mounted to a ScanFish platform that were towed and undulated behind the R/V Pelican during cruises PE03-NGOMEX, PE04-NGOMEX, PE06-NGOMEX, PE07-NGOMEX, PE09-05, and PE11-06 in the Northern Gulf of Mexico between 2003 and 2010. CTD and MIDAS data were synchronized and merged with simultaneously collected OPC data and aggregated into 1 second time bins. The bottom depth was obtained from the NOAA NCEI coastal relief model. This dataset contains 58 variables and we select the dissolved oxygen concentration to predict. We use 50 time points as training data and make predictions on the next 30 time points. As shown in Fig. 4(a) and Fig. S18, ALM predicts the trend of the oxygen content much better in contrast to the majority of other methods.

- 1. **Ground Ozone Level Dataset**

The eight hour peak set ground ozone level dataset is collected from 1998 to 2004 at the Houston, Galveston and Brazoria area^9^. The dataset contains 72 variables and there are missing values in it. We use the mean of neighbor values to fill these missing values, and select the average temperature and TT index to predict. We use 50 time points as training data and make predictions on the next 30 time points. As shown in Fig. 4(b), Fig. 4(c), Fig. S19 and Fig. S20, ALM predicts the trends more accurately than other methods.

- 1. **Wind Dataset**

The wind speed dataset contains the wind speed (m/s) time series sampled every $\Delta t=10$ minutes between 2010 and 2012 from 155 wind stations (variables) in Japan^10^. We resample the dataset with time interval $\Delta t=1$ hour and select one wind station near Tokyo to predict. We use 100 time points as the training data and make predictions on the next 80 time points. As shown in Fig. 4(d), and Fig. S21, ALM’s performance is better than the majority of other methods.

- 1. **Stock Index Dataset**

This dataset is collected different 1130 stock indexes (variables) with an interval of 1 day except Saturday and Sunday from 2018-05-01 to 2018-11-22. Each stock index is a relative number of stock price statistics that measure and reflect the overall price level of the stock market and its changing trend. We select a representative stock index：the Shanghai Stock Exchange A Share Index (SSEA) to predict. As shown in Fig. 4(e) and Fig. S22, ALM predicts the trends more accurately in contrast to the majority of other methods.

- 1. **Traffic Dataset**

This dataset contains the traffic speed collected from 207 loop detectors (variables) in the highway of the Los Angeles County^11^. We train our model on 50 time points at 6 locations respectively and make the predictions for the next 10 time points. As shown in the movie listed in Section 3.2.8, Fig. S31 and Table VIII, compared with other methods our ALM predicts the traffic flow accurately. It should be noted that some methods, such as Lasso, Adalasso and Varm, have higher Pearson correlation coefficients on some locations, but their prediction curves always appear as a straight line as shown in Fig. S23-S28, which demonstrates that they did not predict any trends. And our ALM always gets higher scores on the composite indicators than other methods. Movie-Traffic Image is attached to the following link: <https://github.com/AnticipatedLearningMachine/Anticipated-Learning-Machine> .

- 1. **Satellite cloud image Dataset**

This dataset contains satellite cloud images collected by National Institute of Informatics and we select the typhoon Marcus to predict^12^. The dataset is composed of a series of 241 cloud images (2402 variables) from 2018.3.15 to 2018.3.24 with one image taken per hour. The model is trained on 50 time points and predictions for the next 27 time points are made. As shown in the movie listed in Section 3.2.9, Figs. S29-S30 and Table IX, ALM predicts the route of typhoon eye more accurately than other methods. Movie-Satellite Image is attached to the following link: <https://github.com/AnticipatedLearningMachine/Anticipated-Learning-Machine> .

1. **Experiment Results**
   1. **Comparison Methods**

We compare ALM with the following 12 methods including the neural network method.

ARIMA^13^: a well-known single-variable auto-regressive model for predicting future time series. The ARMA(p,q) process after being differenced D times is denoted by ARIMA(p,D,q) and its form is

$$\Delta D y_{t}=c+\phi_{1}\Delta D y_{t-1}+\ldots+\phi_{p}\Delta D y_{t-p}+\varepsilon_{t}+\theta_{1}\varepsilon_{t-1}+\theta_{q}\varepsilon_{t-q},$$

where $\Delta D y_{t}$ denotes a $D$th differenced time series, $\varepsilon_{t}$ represents an uncorrelated innovation process with mean zero.

VAR^14^: a vector multi-variable auto-regressive model which captures the pairwise relationships among all variables. A VAR(p) model in the difference-equation notation and in the reduced form is

$$y_{t}=c+\beta x_{t}+\sum_{i=1}^{p} \phi_{i}y_{t-i}+\varepsilon_{t},$$

where $c$ is a constant vector, $\phi_{j}$ is an autoregressive coefficients matrix, $x_{t}$ is exogenous predictor variables, $\beta$ is a regression coefficient matrix, and $\varepsilon_{t}$ is random Gaussian innovation.

MA: The moving average uses the unweighted mean of the previous data to make predictions, and it is a single-variable method. The form of MA(q) is

$$y_{t}=\frac{y_{t-1}+y_{t-2}+\ldots+y_{t-q}}{q}.$$

SES^15,16^: The Holt-Winters exponential smoothing uses a weighted moving average with exponentially decreasing weights of the previous data to make predictions, and it is a single-variable method. The simplest form of the exponential smoothing is given by:

$$s_{0}=x_{0},$$

$$s_{t}=\alpha x_{t}+\left( 1-\alpha\right)s_{t-1}, t>0,$$

where $\{x_{t}\}$ is the original time series, $\{s_{t}\}$ is the output of the exponential smoothing algorithm, and $\alpha$ is the smoothing factor.

VARM^17^: The basic process of VARMAX includes the autoregressive process, the moving average process, and the independent exogenous terms (other unmodeled inputs). VARMAX is a multi-variable method and the form of VARMAX(p,q) is

$$y_{t}=c+\beta x_{t}+\sum_{i=1}^{p} \phi_{i}y_{t-i}+\sum_{j=1}^{q} \Theta_{j}\varepsilon_{t-j}+\varepsilon_{t},$$

where $c$ is a constant vector, $\phi_{j}$ is an autoregressive coefficients matrix, $x_{t}$ is exogenous predictor variables, $\beta$ is a regression coefficient matrix, $\Theta_{j}$ is a moving average matrix, and $\varepsilon_{t}$ is random Gaussian innovation.

SVR^18^: Support Vector Regression (SVR) uses SVM to fit curves and perform regression analysis. It is a multi-variable method and the function used to predict new values is

$$f\left( x \right)=\sum_{n=1}^{N} \left( \alpha_{n}-\alpha_{n}^{*} \right)G\left( x_{n},x \right)+b,$$

where $x_{n}$ is a multivariate set of N observations with observed values $x$ and $G\left( x_{n},x \right)=\exp\left( -\left\| x_{n}-x \right\|^{2} \right).$

SVE^19^: The classic single-variable embedding. The prediction is based on the trend of delay coordinates and only the time series of the target variable is used to make the predictions.

MVE^20^: The recently proposed multi-view embedding. It is a multi-variable method and the prediction is based on the trend of reconstructed attractors and all the variables are used to make the predictions.

RDE^21^: The recently proposed multi-variable model for one-step-ahead prediction of short-term high-dimensional time series. Given the time series $\mathbf{X}\left( t \right)=[x_{1}\left( t \right),x_{2}\left( t \right),\ldots,x_{n}(t)]$, denote $t_{i}=t_{i-1}+\tau$, and suppose $x_{k}$ is the variable to be predicted. RDE first randomly selects *s* tuples from$(1,2,\ldots,n)$, then for each tuple, a predictor $\psi$ is fitted. The final prediction made by RDE is based on these predictors.

LSTM^22^: A famous neural network which is widely used in the field of time series analysis. It is a multi-variable method.

Lasso^23^: Lasso procedure is used to estimate the parameters of AR(p) and make predictions. It is a single-variable method.

AdaLasso^23^: AdaLasso procedure is used to estimate the parameters of AR(p) and make predictions. It is a single-variable method.

Here, we should mention that, ALM can also be regarded as an extended version of RDE. While RDE is essentially a one-step-ahead prediction framework and trains different $\boldsymbol{\Psi}_{i}$s independently, ALM uses consistent-training to simultaneously train different $\boldsymbol{\Psi}_{i}$s, which enables ALM to capture the system’s dynamics more globally and consequently become an efficient multi-step-ahead prediction method. Moreover, compared with the Gaussian Process Regression used in the RDE method, ALM adopts Neural Networks, a more general framework of learning, to fit $\boldsymbol{\Psi}$, and the neural network framework is more capable of capturing the complex nonlinear relationship. Therefore, ALM outperforms RDE even with a small number of samples.

- 1. **Results on Each Datasets**

In our experiments, we split the target variables into several segments to predict the future values due to the time-variant properties of different system, and some sudden changes caused by external factors in systems will thus be included during training. Due to the different impacts of external factors on different datasets, the more external factors affect the dataset, the more segments we divide. For each dataset, we plot the predictions made by 12 comparison methods and illustrated the MAE, RMSE and Pearson Correlation Coefficient, Spearman Correlation Coefficient and Composite Indicator for each method in Figs. S4-S31. Note that the composite indicator is constructed based on all above criteria in order to achieve a comprehensive measurement of numerical values and trends. Specifically, we vote for each method based on four basic indicators, and the better the basic indicators, the higher the number of votes. The value of the composite indicator is the sum of votes of the four basic indicators. Therefore, a higher value of composite indicator demonstrates a better performance of a method. And Tables I-IX with Movie-Traffic & Movie-Satellite Images attached to the following link: <https://github.com/AnticipatedLearningMachine/Anticipated-Learning-Machine> .

- - 1. **Noise Free Coupled Lorentz System**

The MAE, RMSE and Pearson Coefficient Index for each method are shown in Table I. The prediction for each comparison method is plotted in Fig. S4, Fig. S5 and Fig. S6.

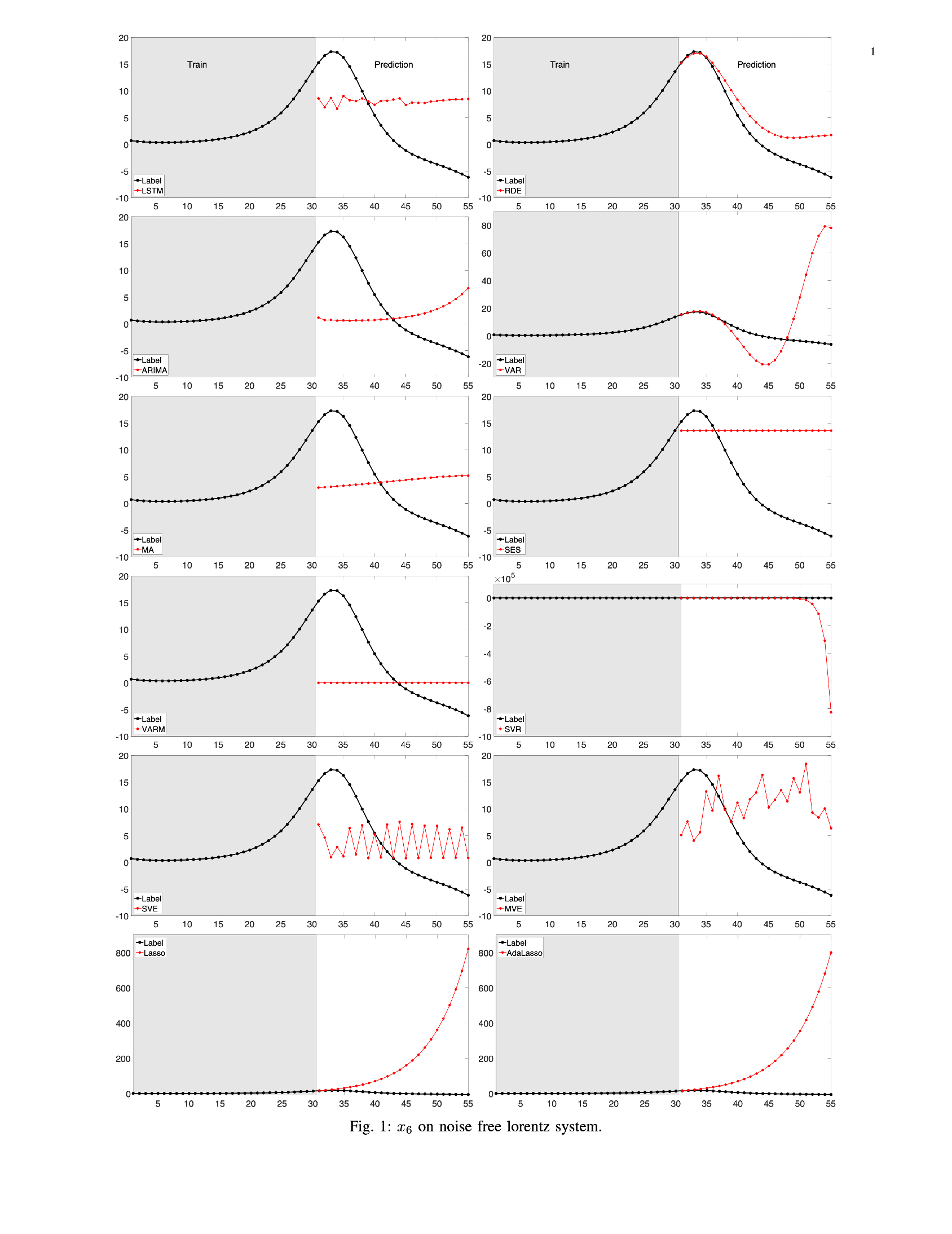


Fig. S4: The performance of all methods to predict $x_{6}$on the noise-free Lorentz system.


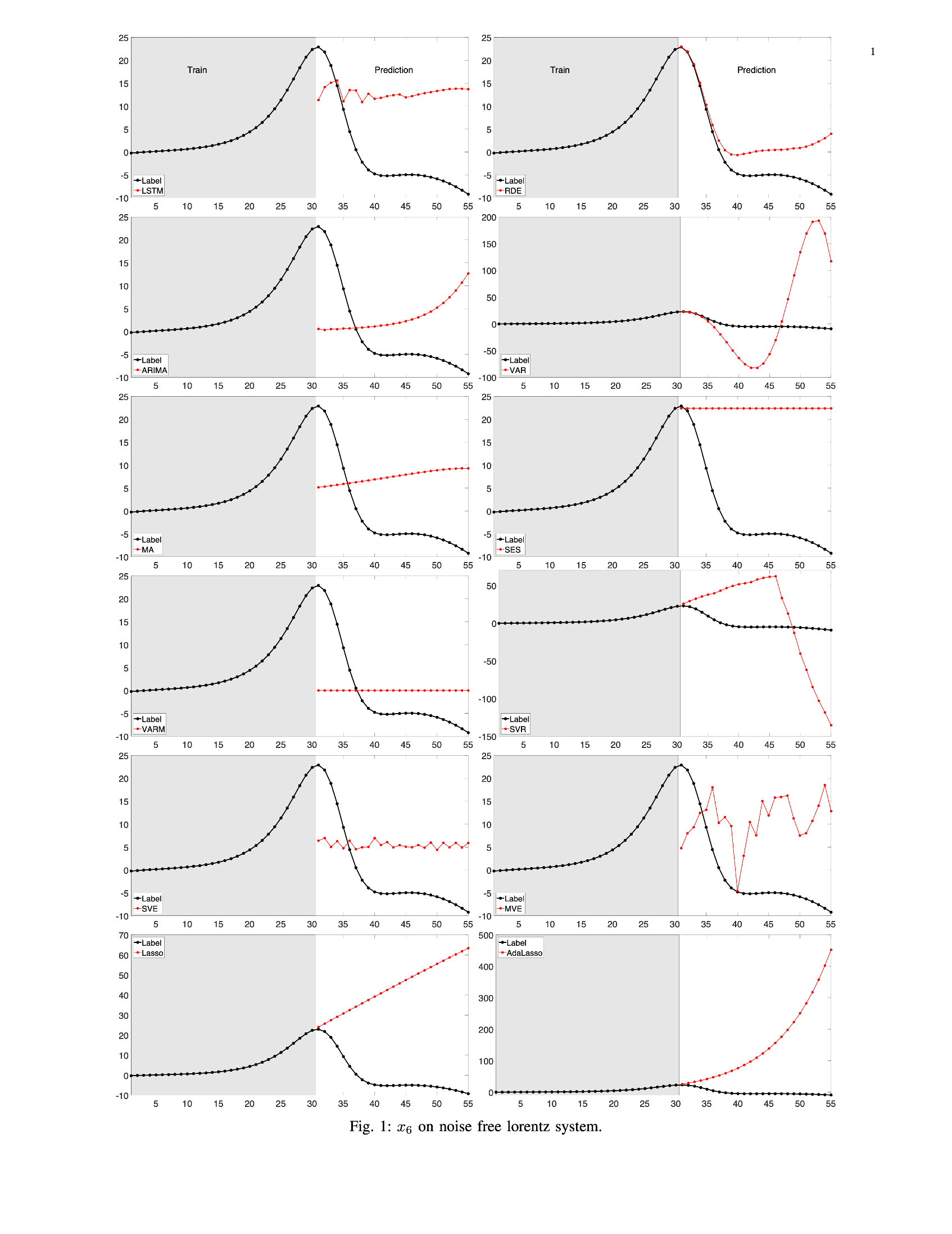


Fig. S5: The performance of all methods to predict $y_{6}$ on the noise-free Lorentz system.


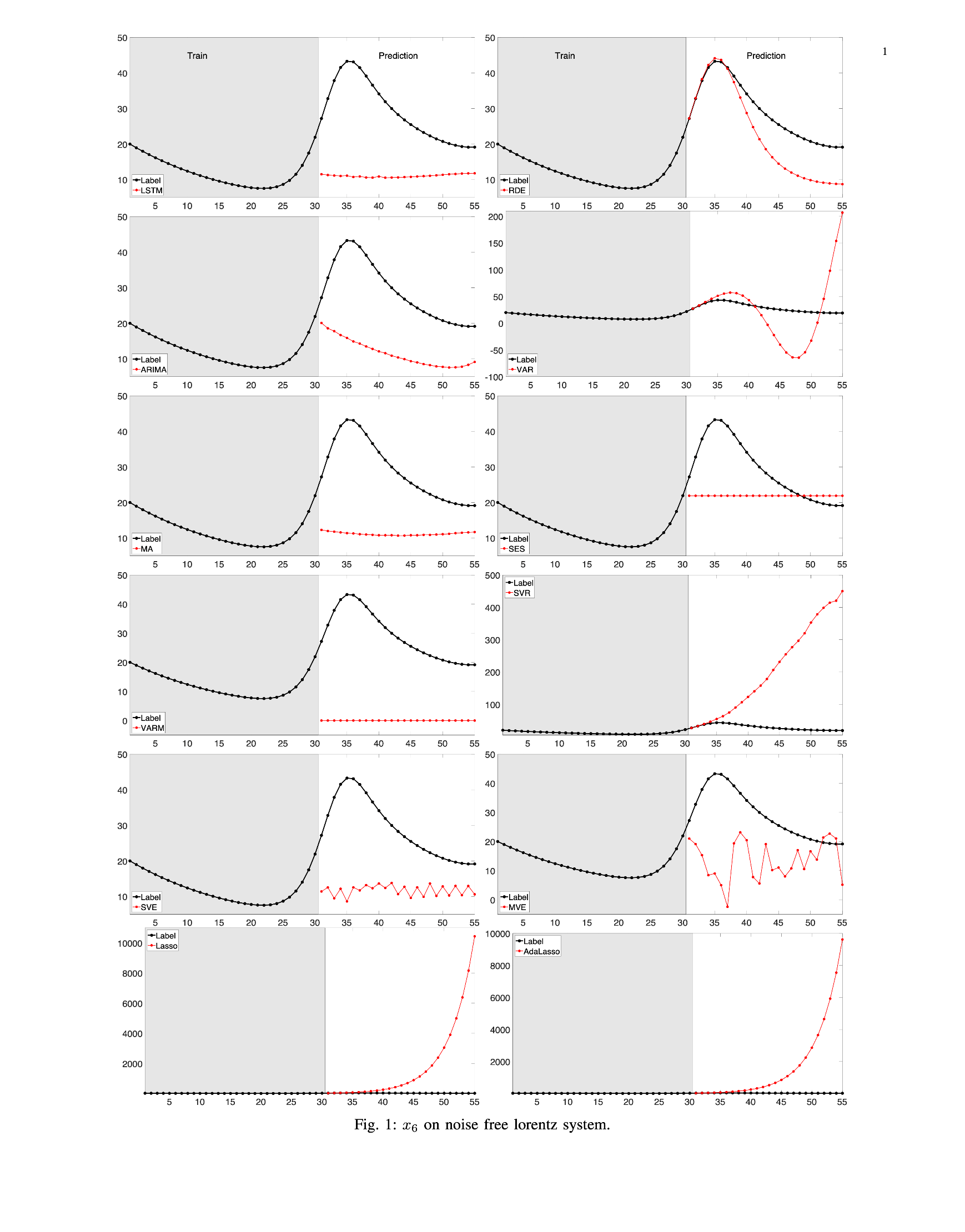


Fig. S6: The performance of all methods to predict $z_{6}$ on the noise-free Lorentz system.

- - 1. **Coupled Time-variant Lorentz System with Noise**

The MAE, RMSE and Pearson Coefficient Index for each method are shown in Table II. The prediction for each comparison method is plotted in Fig. S7, Fig. S8 and Fig. S9.


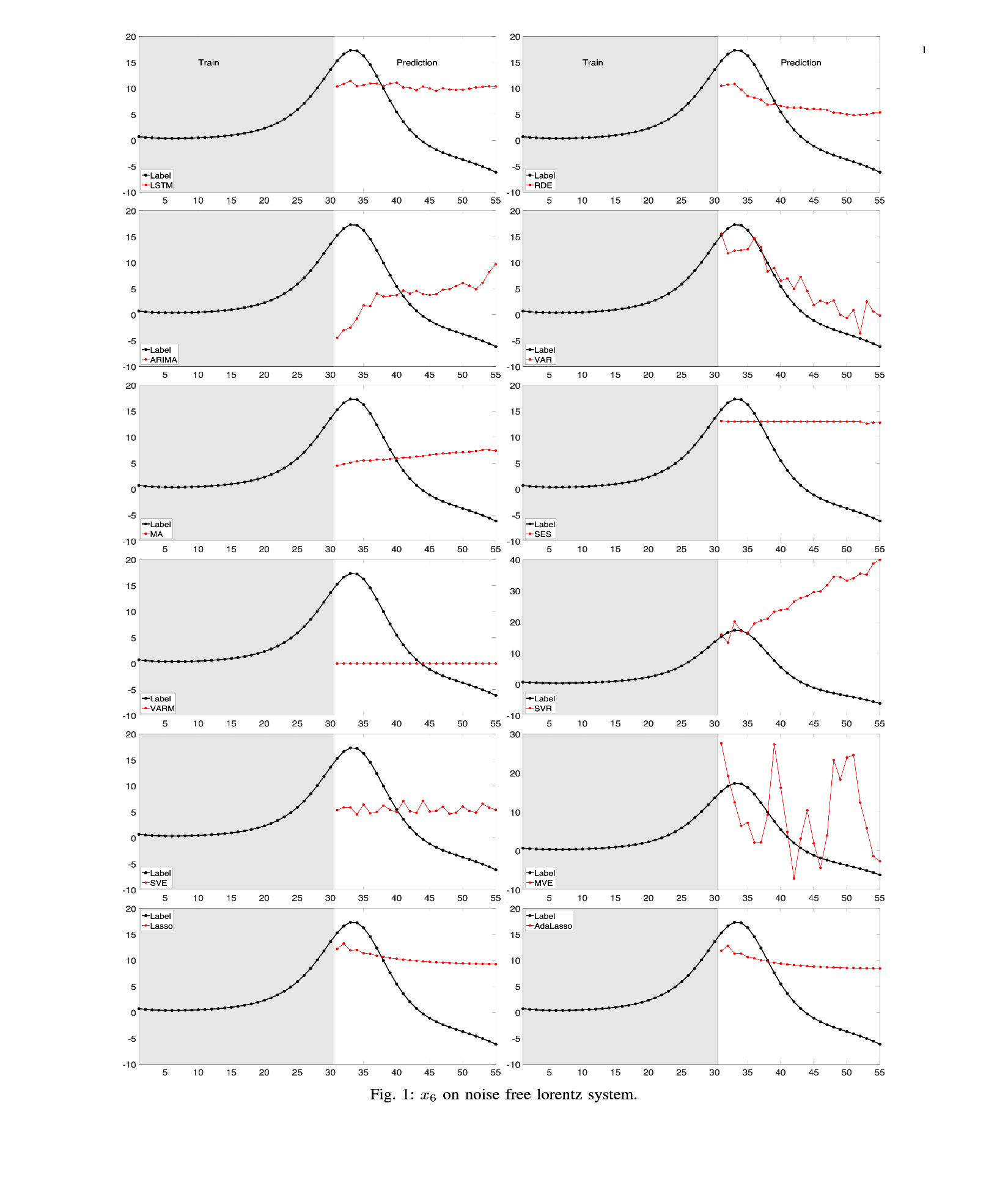


Fig. S7: The performance of all methods to predict $x_{6}$ on the Lorentz system with noise.


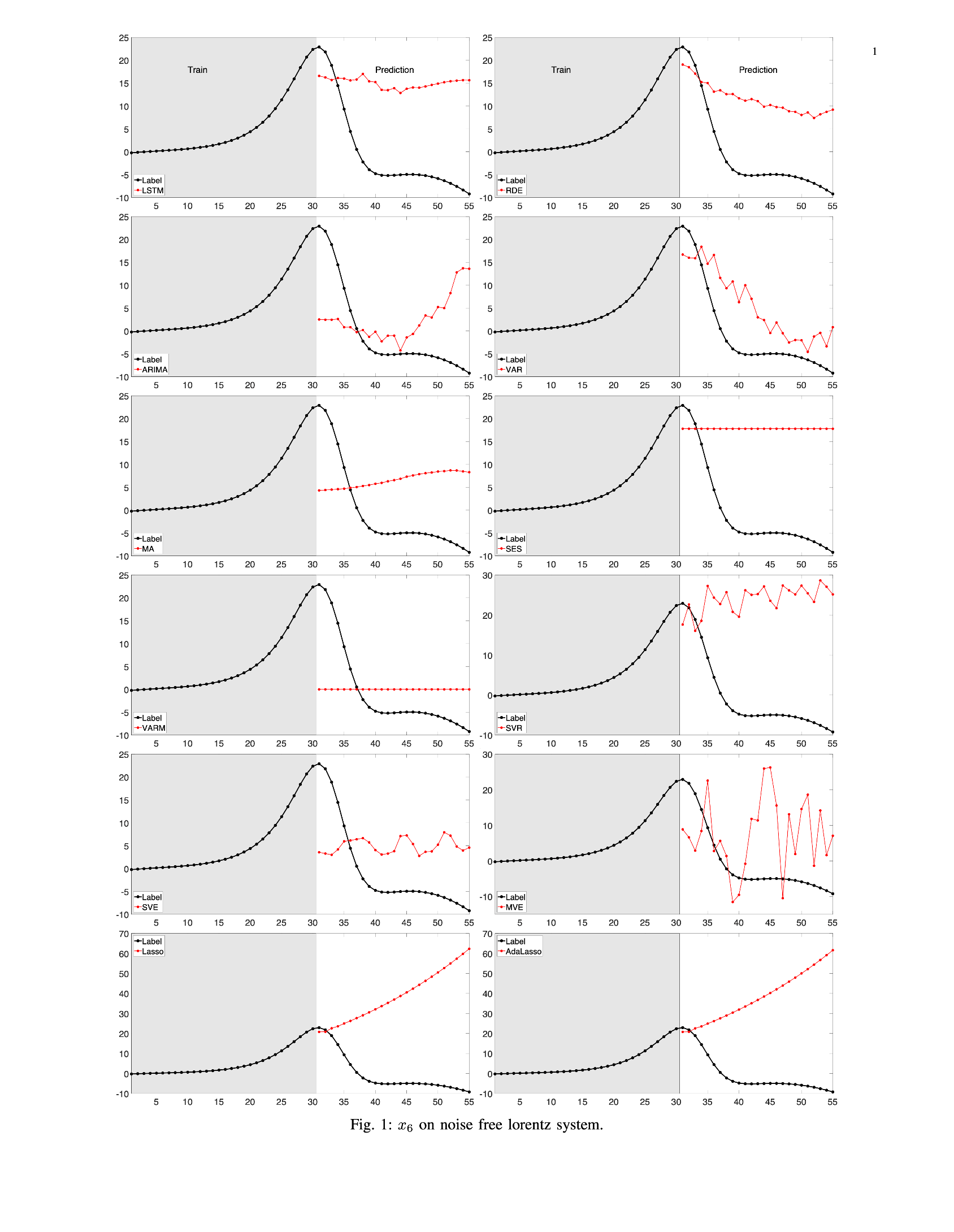


Fig. S8: The performance of all methods to predict $y_{6}$ on the Lorentz system with noise.


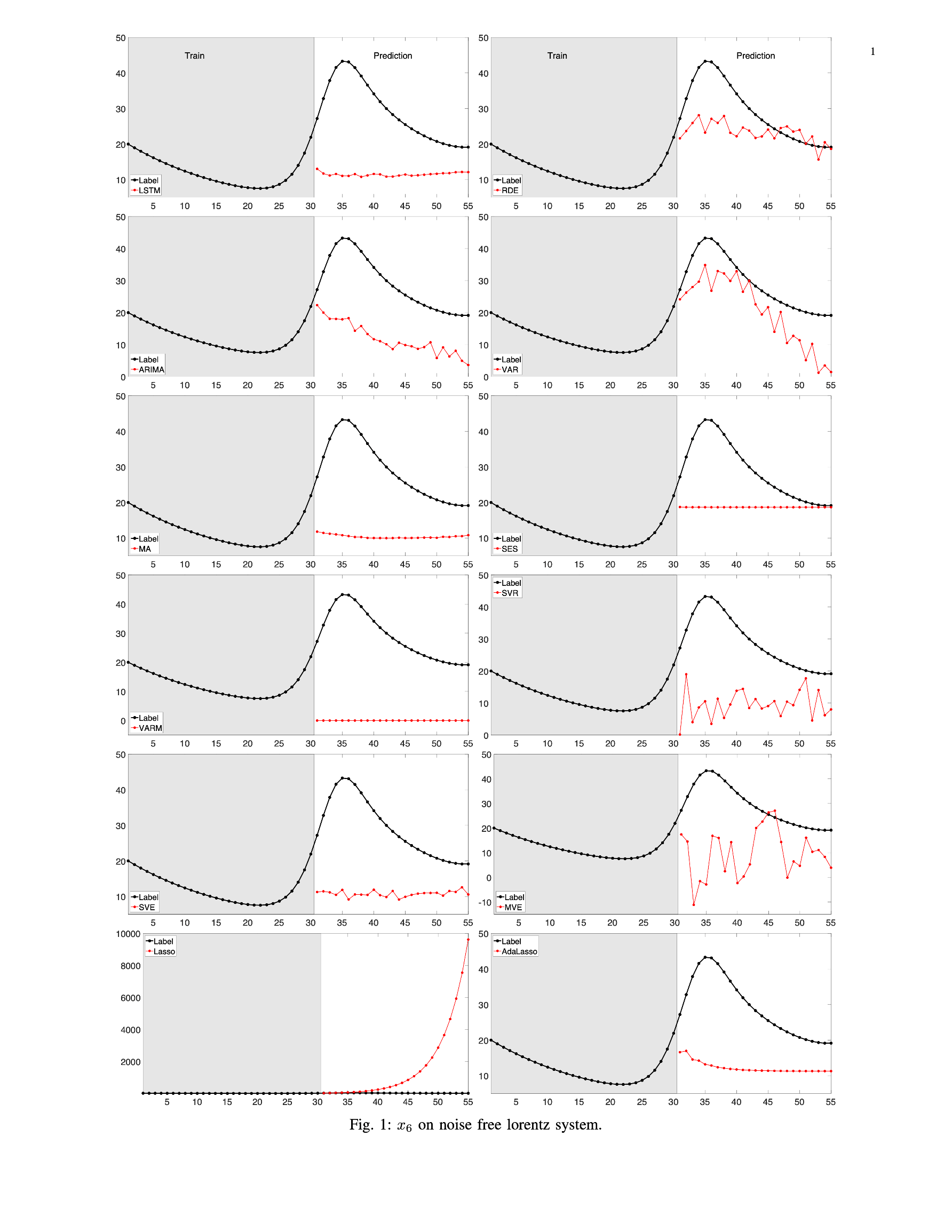


Fig. S9: The performance of all methods to predict $z_{6}$ on the Lorentz system with noise.

- - 1. **Gene Dataset of Rats**

The MAE, RMSE and Pearson Coefficient Index for each method are shown in Table III. The predictions for each comparison method are plotted in Figs. S10 -S17.


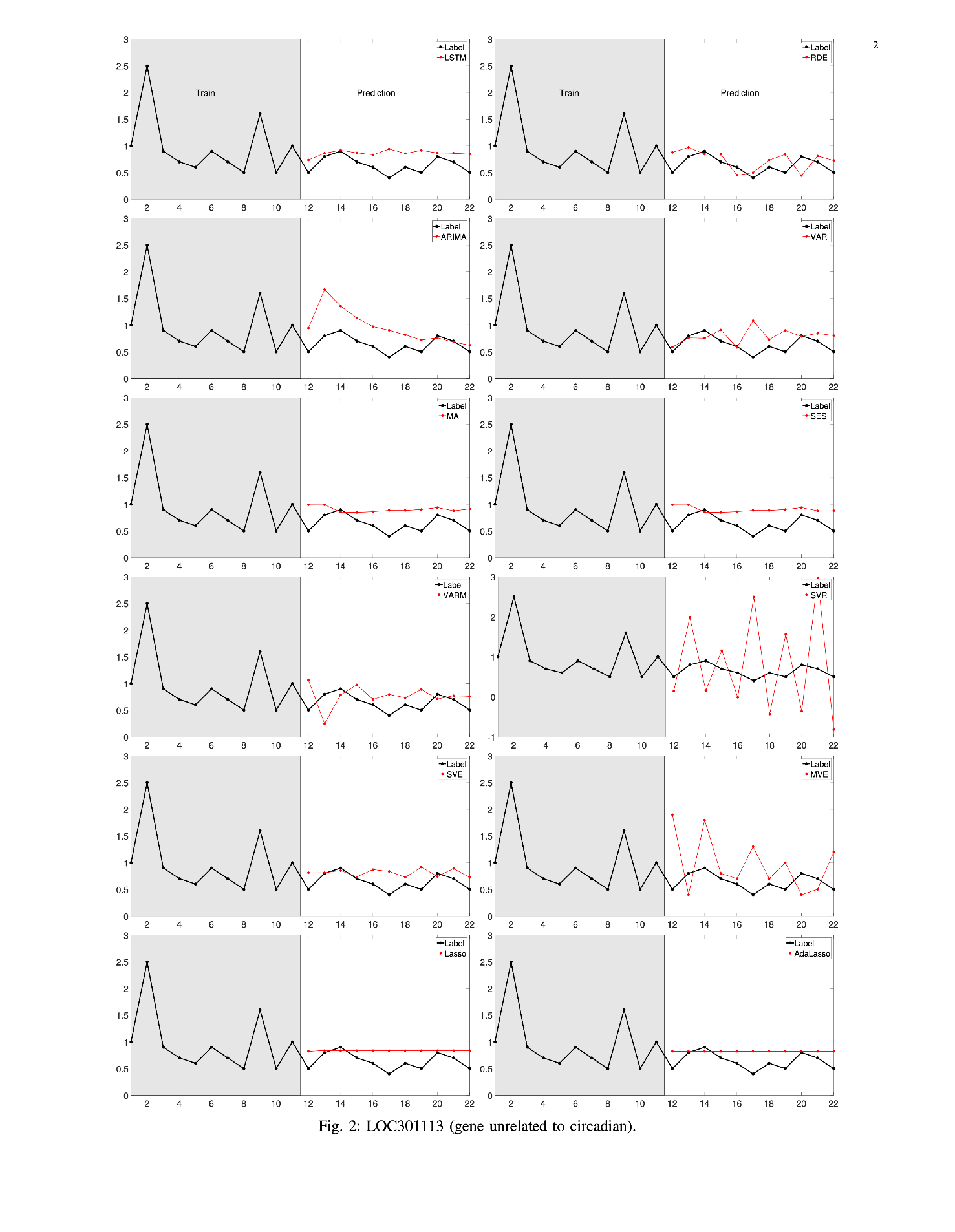


Fig. S10: The performance of all methods to predict LOC301113 (a gene unrelated to circadian rhythm).


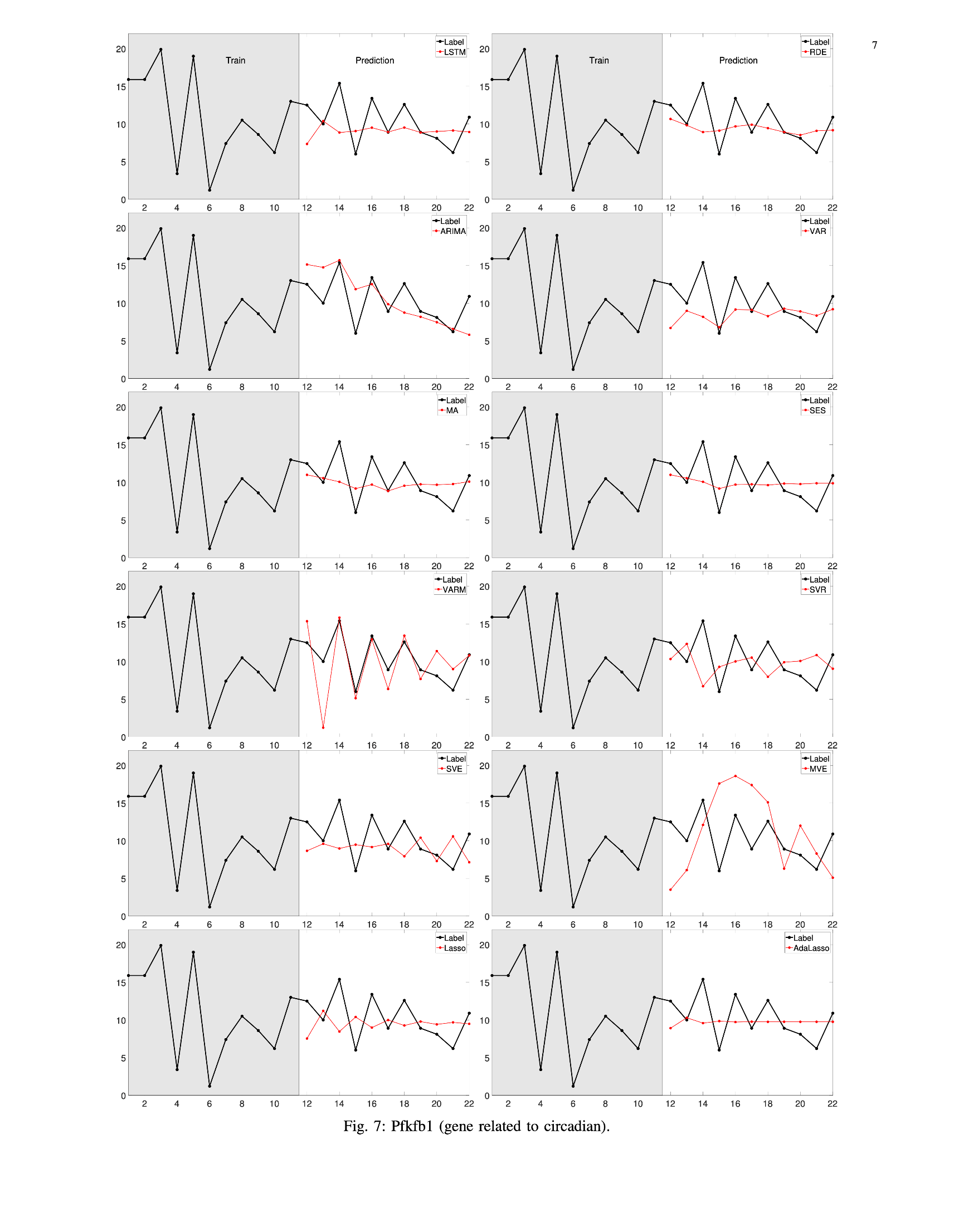


Fig. S11: The performance of all methods to predict Pfkfb1 (a gene related to circadian rhythm).


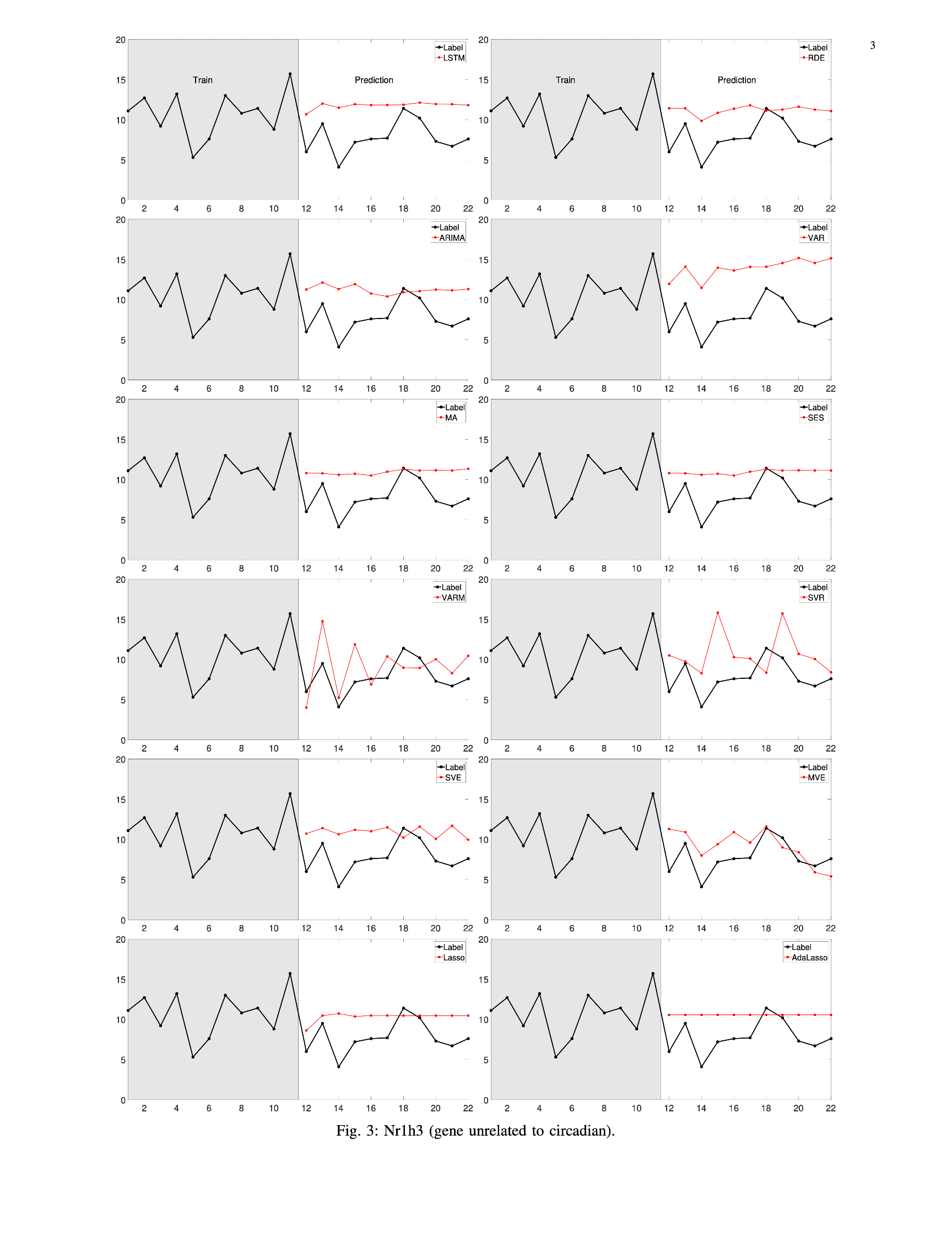


Fig. S12: The performance of all methods to predict Nr1h3 (a gene unrelated to circadian rhythm).


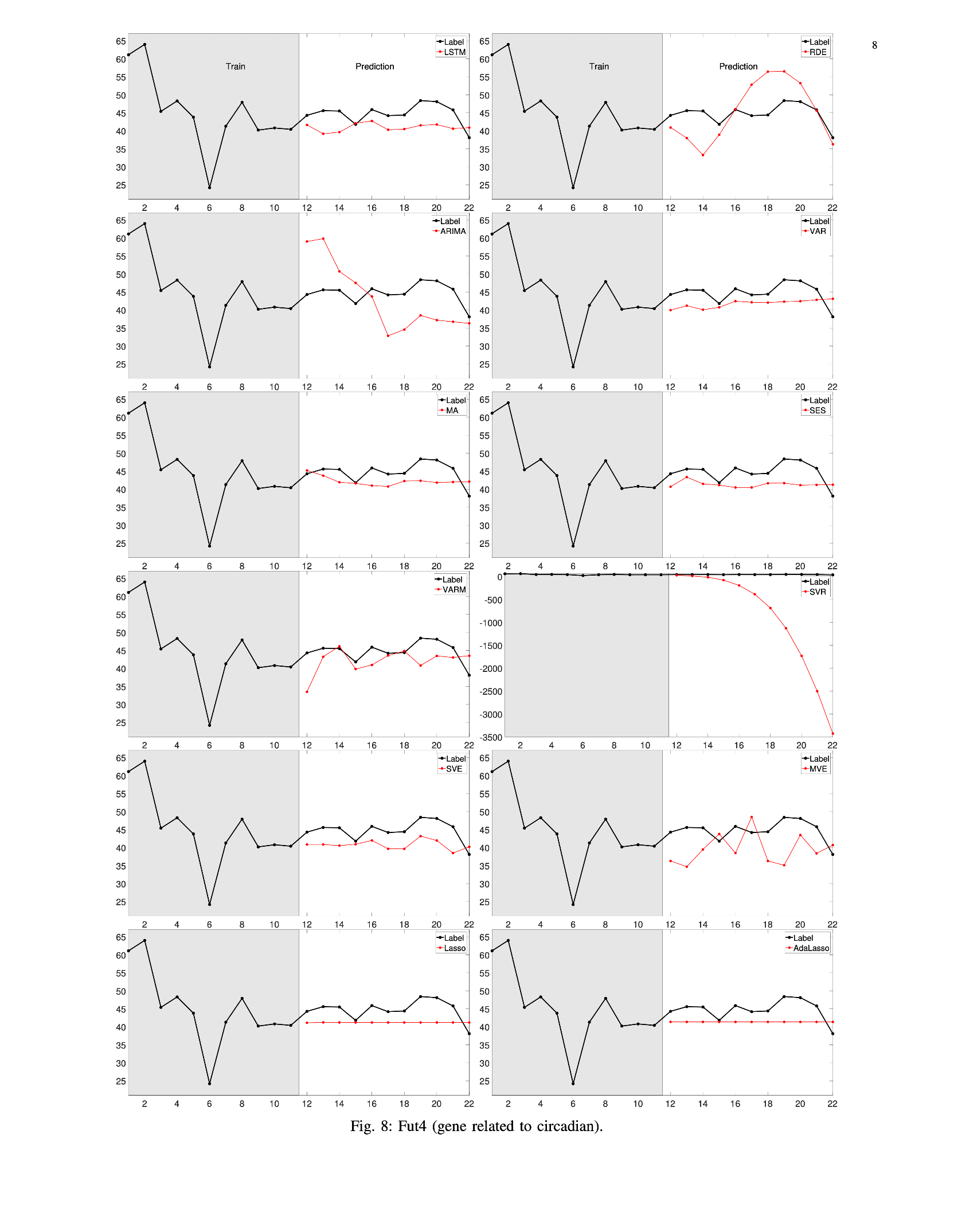


Fig. S13: The performance of all methods to predict Fut4 (a gene related to circadian rhythm).


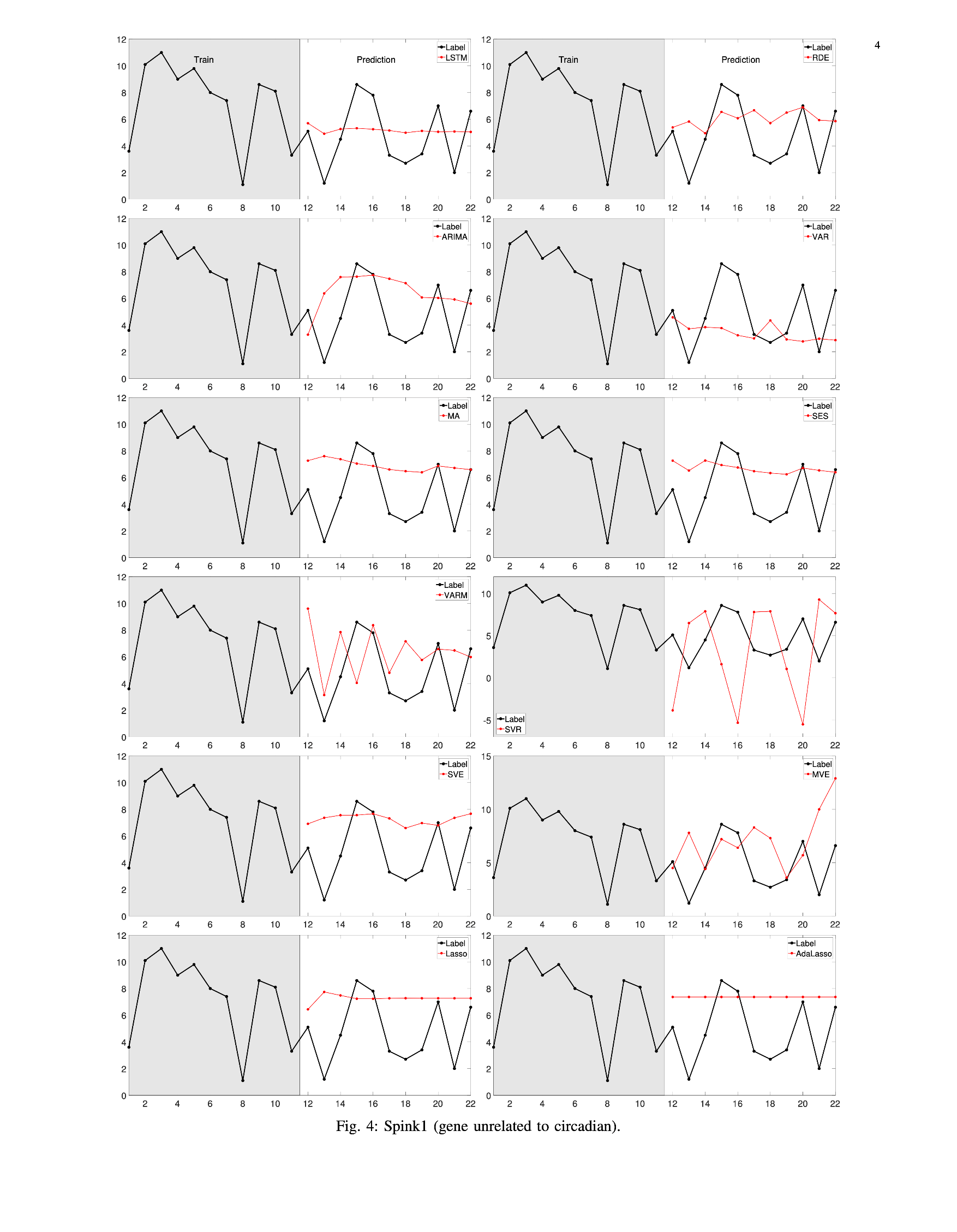


Fig. S14: The performance of all methods to predict Spink1 (a gene unrelated to circadian rhythm).


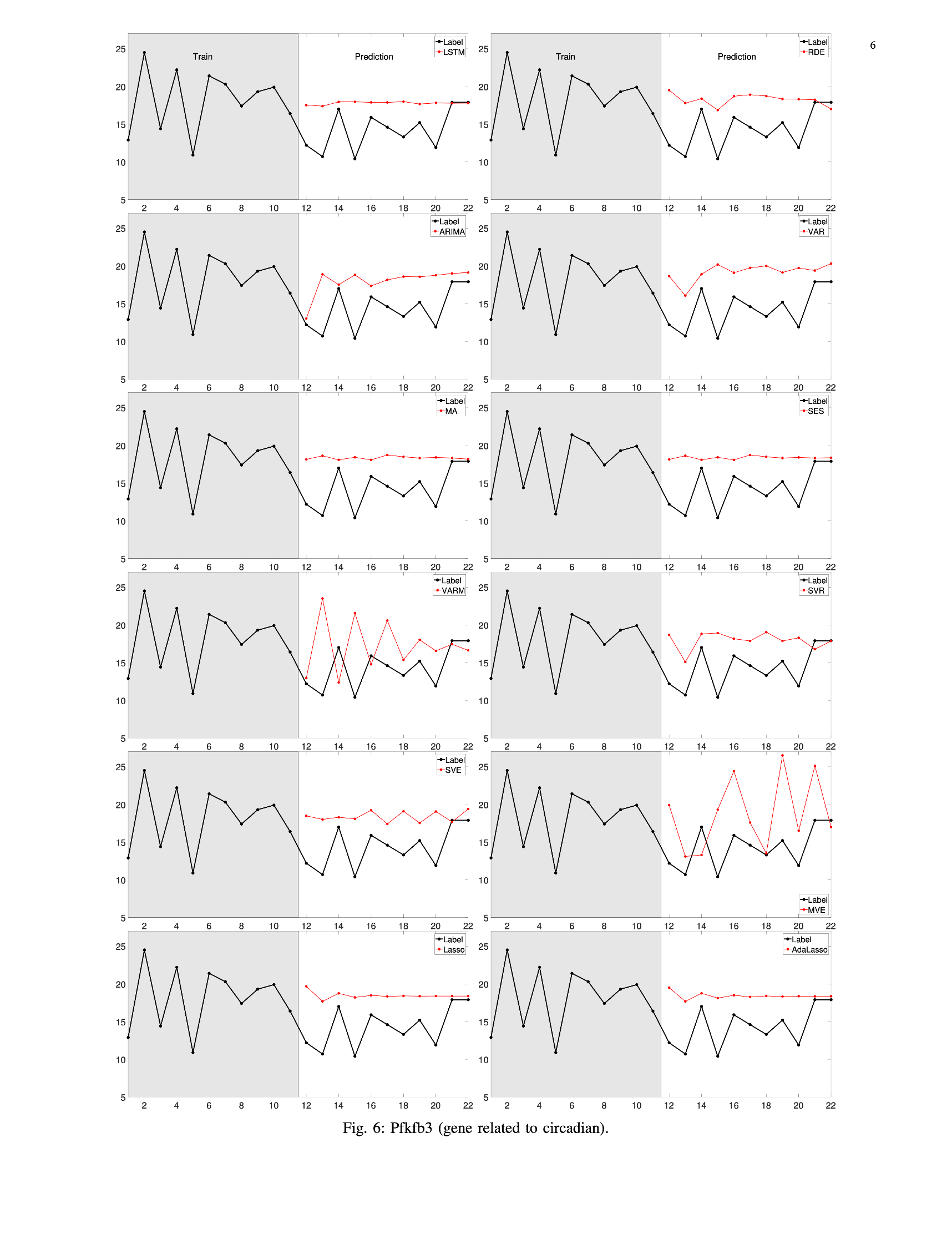


Fig. S15: The performance of all methods to predict Pfkfb3 (a gene related to circadian rhythm).


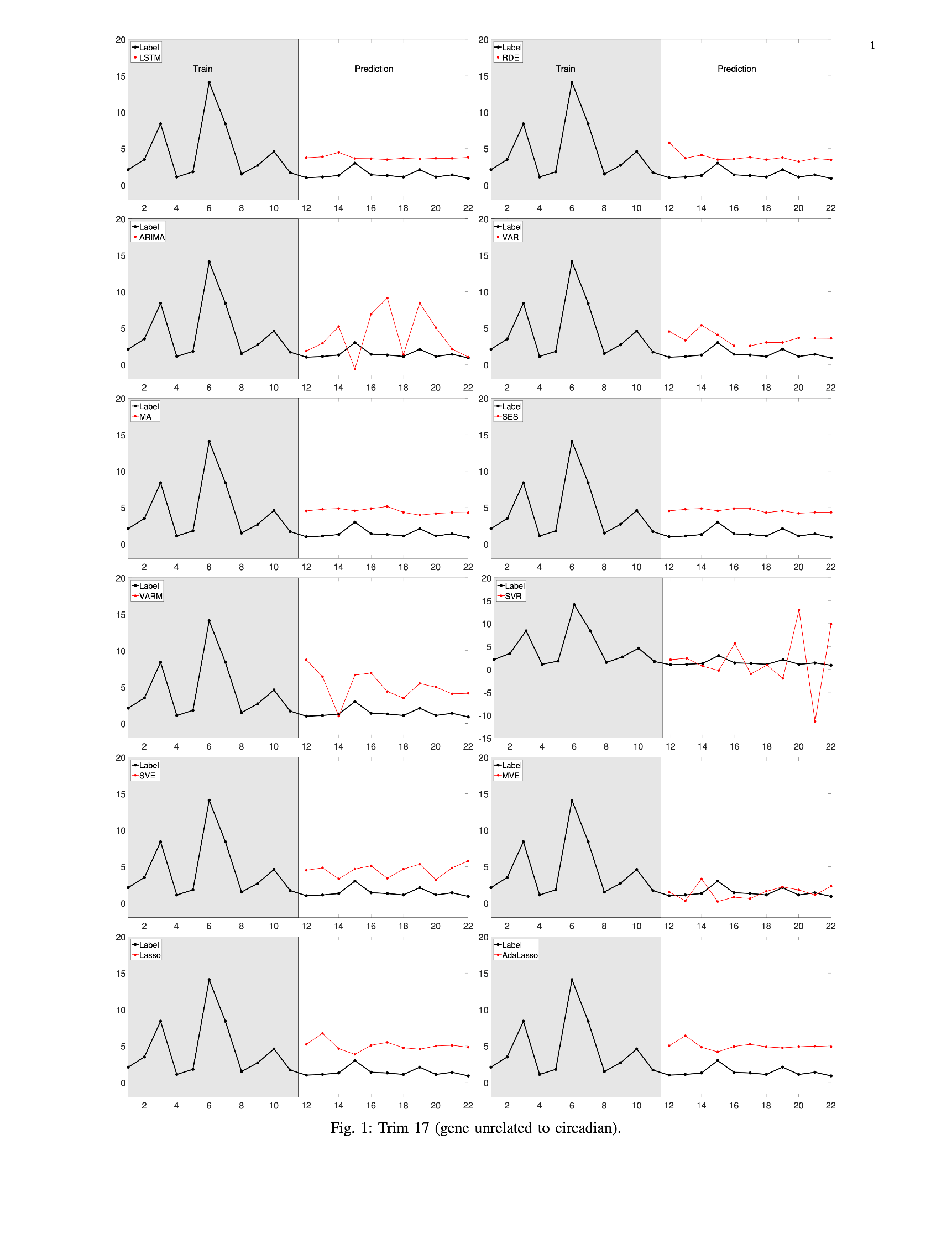


Fig. S16: The performance of all methods to predict Trim17 (a gene unrelated to circadian rhythm).


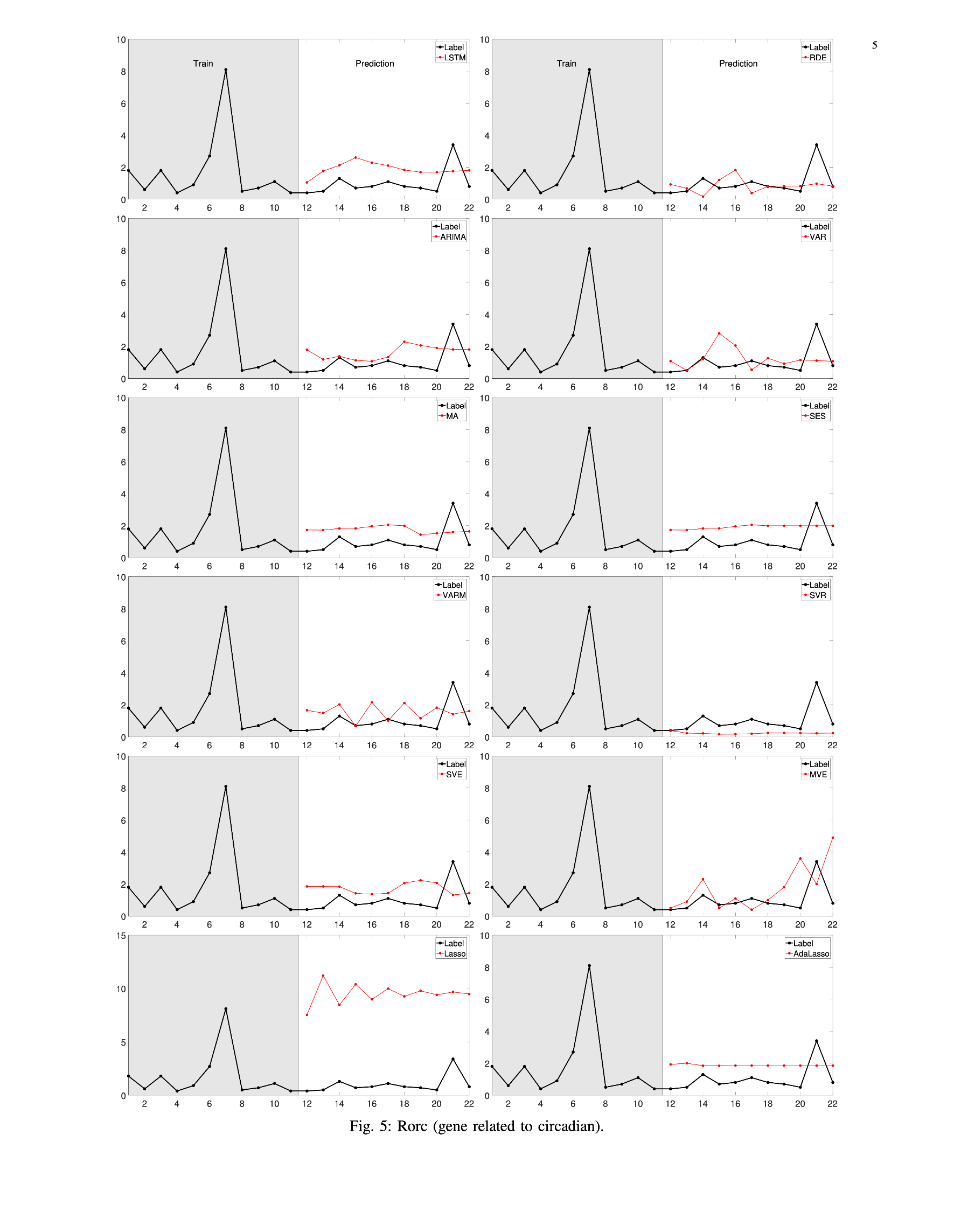


Fig. S17: The performance of all methods to predict Rorc (a gene related to circadian rhythm).

- - 1. **Plankton Dataset**

The MAE, RMSE and Pearson Coefficient Index for each method are shown in Table IV. The predictions for each comparison method are plotted in Fig. S18.


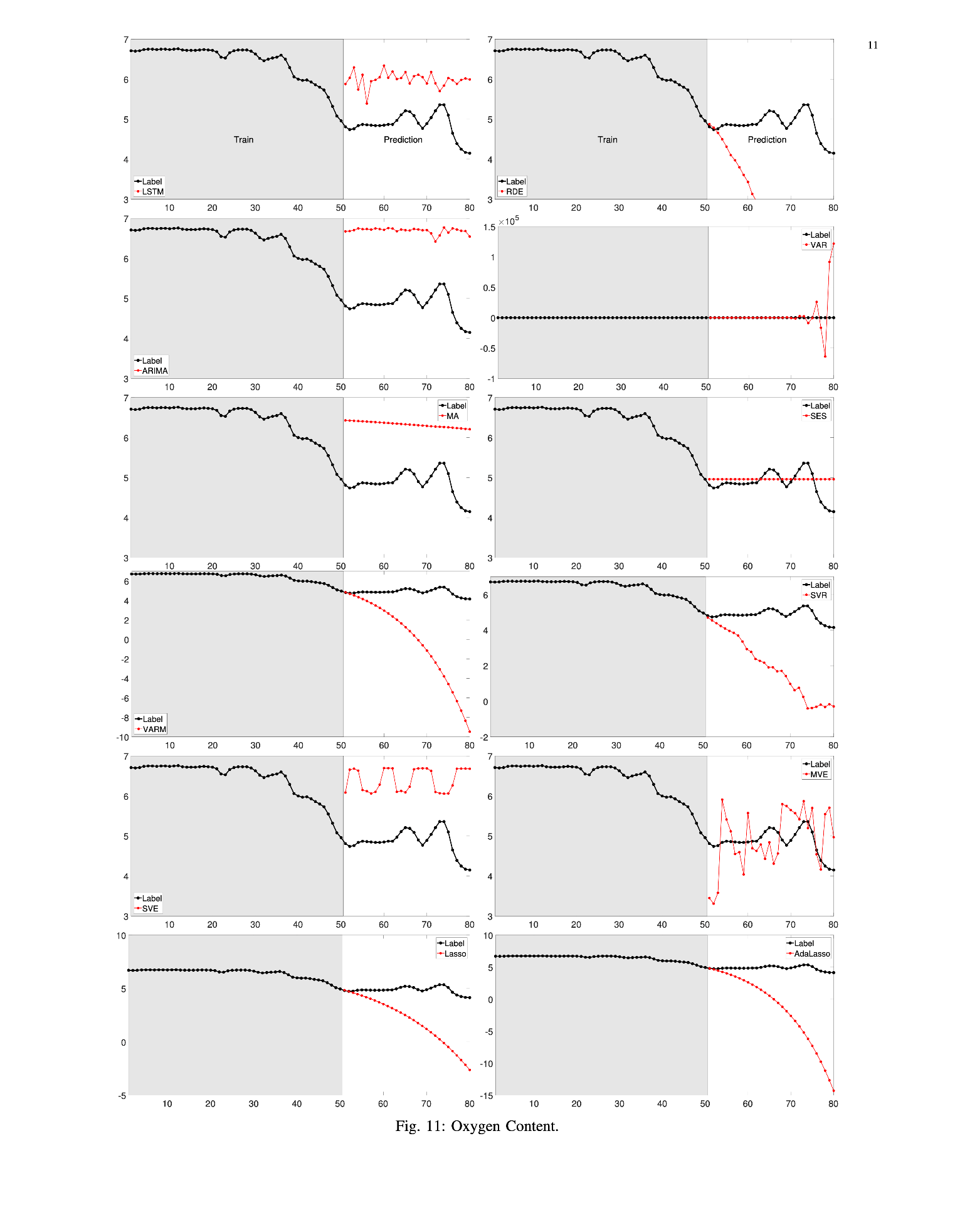


Fig. S18: The performance of all methods to predict the oxygen content.

- - 1. **Ground Ozone Level Dataset**

The MAE, RMSE and Pearson Coefficient Index for each method are shown in Table V. The predictions for each comparison method are plotted in Fig. S19 and Fig. S20.

**
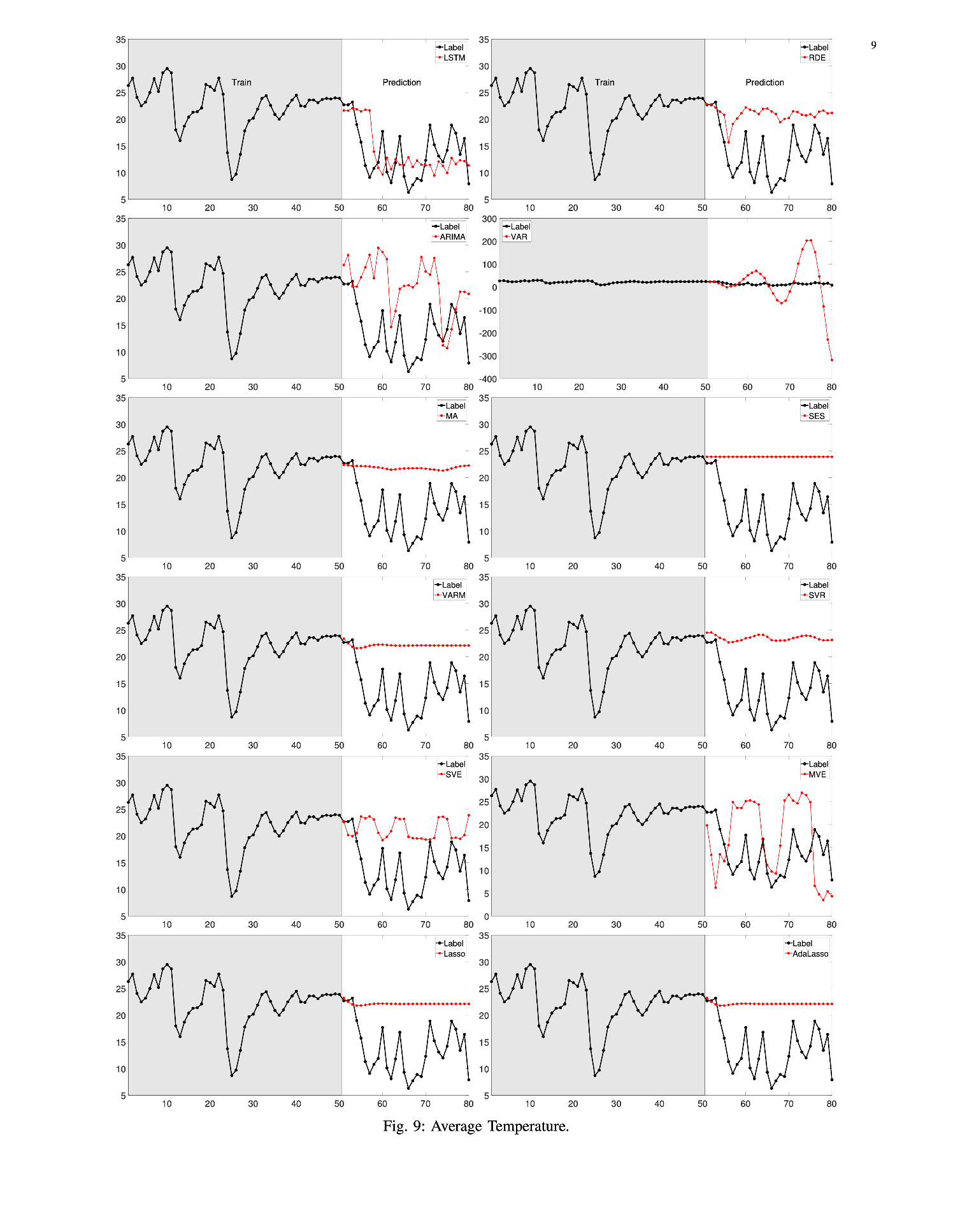
**

Fig. S19: The performance of all methods to predict the average temperature.


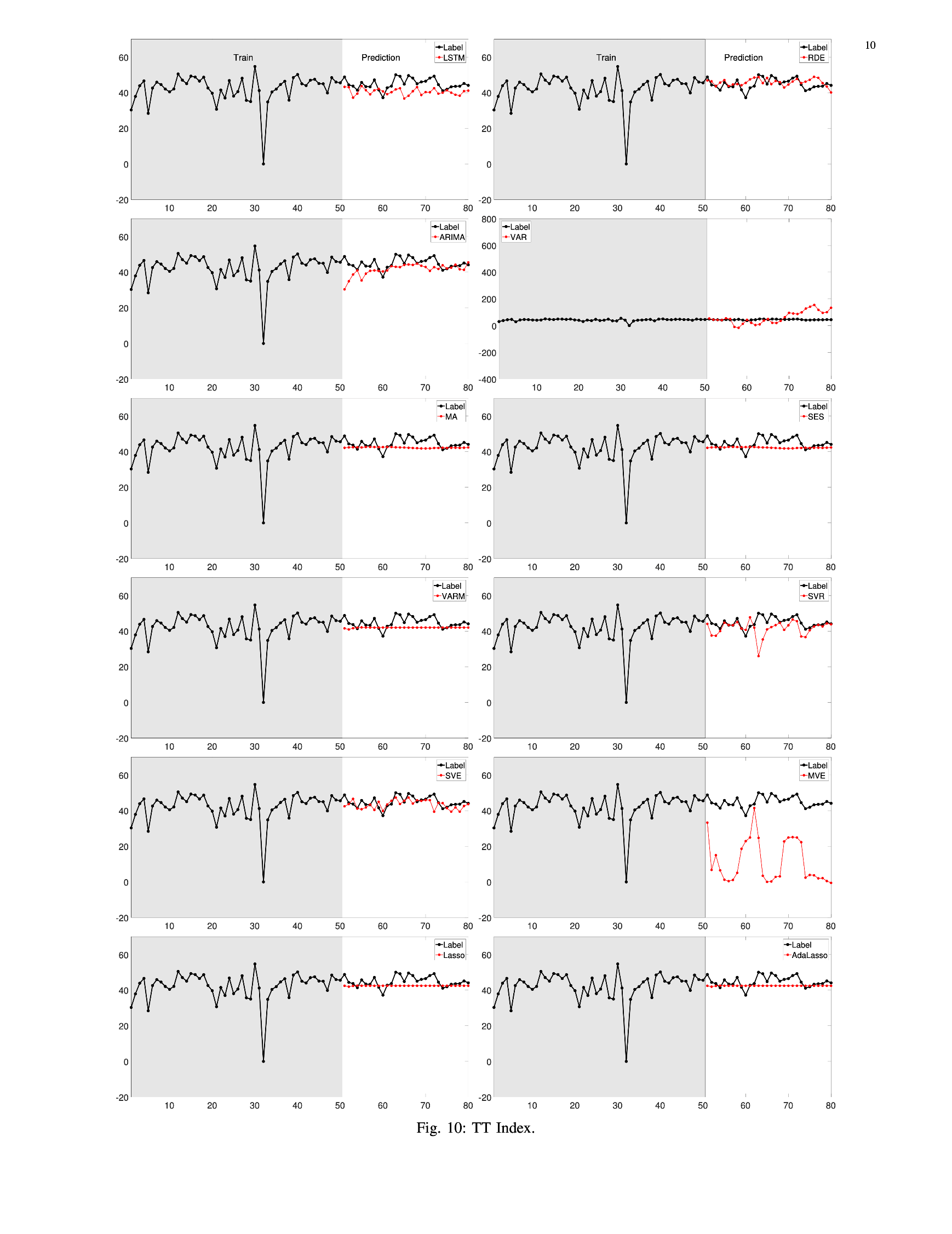


Fig. S20: The performance of all methods to predict the TT Index.

- - 1. **Wind Speed Dataset**

The MAE, RMSE and Pearson Coefficient Index for each method are shown in Table VI. The predictions for each comparison method are plotted in Fig. S21.


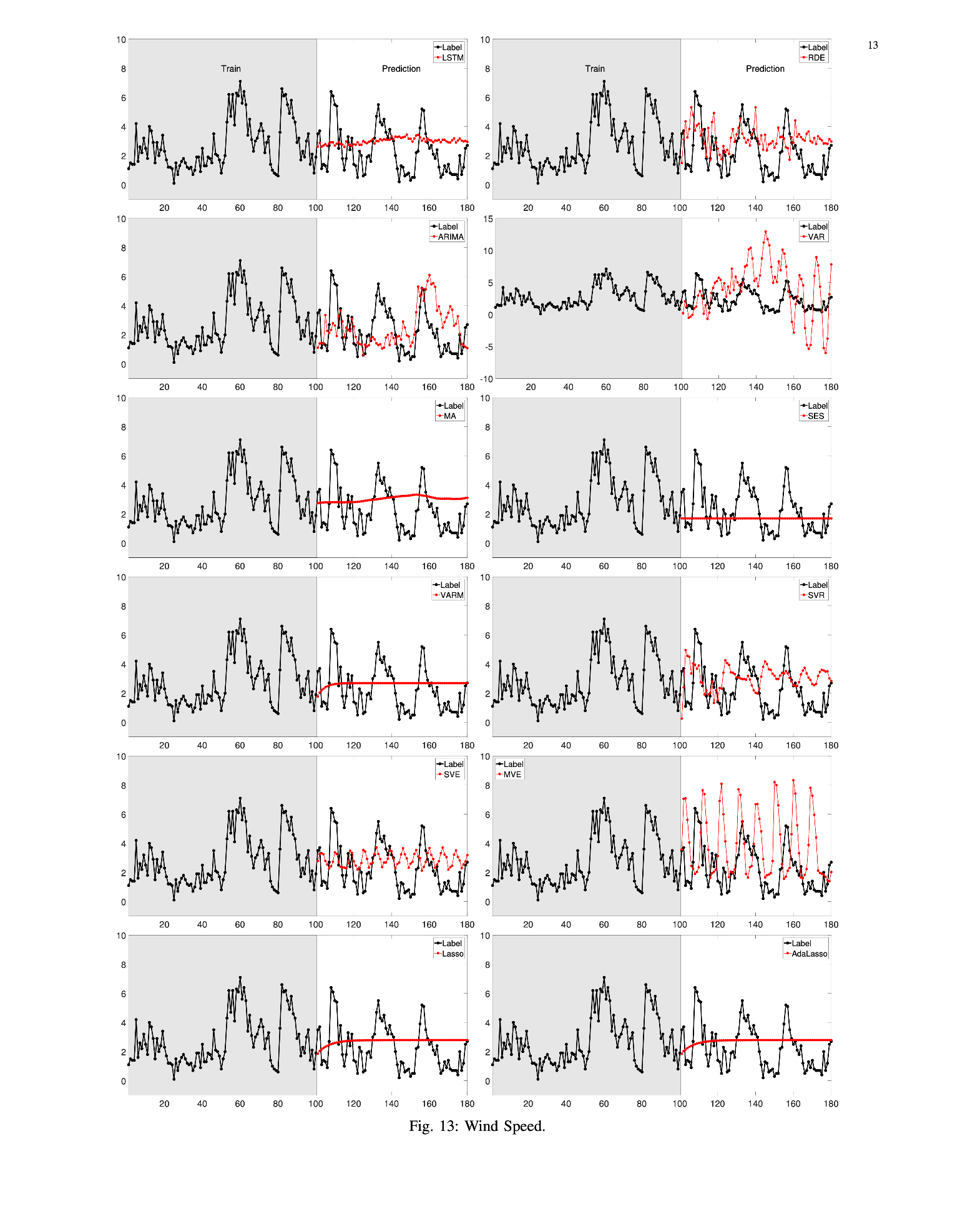


Fig. S21: The performance of all methods to predict the wind speed.

- - 1. **Stock Index Dataset**

The MAE, RMSE and Pearson Coefficient Index for each method are shown in Table VII. The predictions for each comparison method are plotted in Fig. S22.


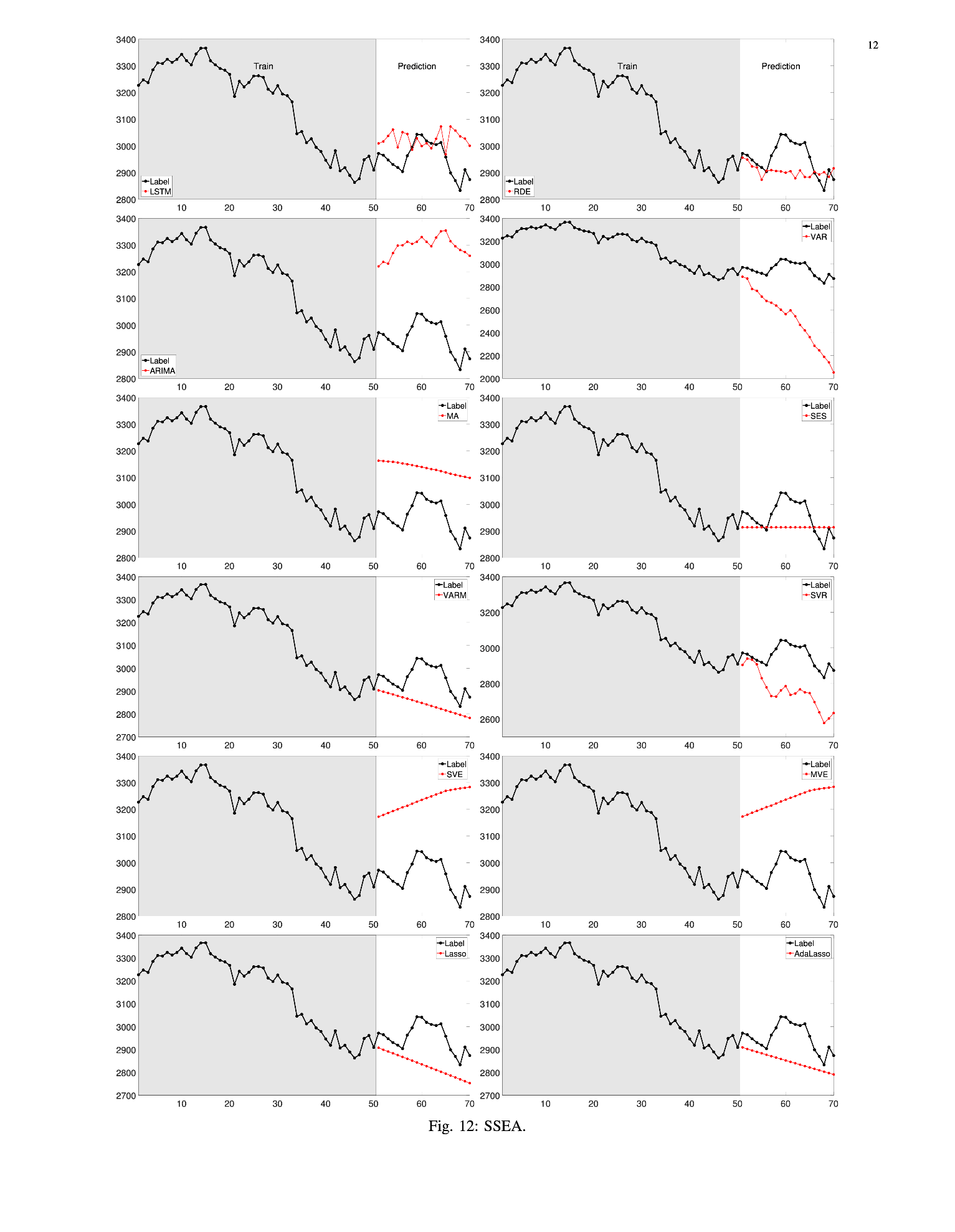


Fig. S22: The performance of all methods to predict the SSEA.

- - 1. **Traffic Dataset**

The MAE, RMSE and Pearson Coefficient Index for each method are shown in Table VIII and all the predictions made by AL are illustrated in Fig. S31. The predictions for each comparison method are plotted in Figs. S23 –S28. The corresponding movie for the traffic prediction is also uploaded to the website:

https://github.com/AnticipatedLearningMachine/Anticipated-Learning-Machine.


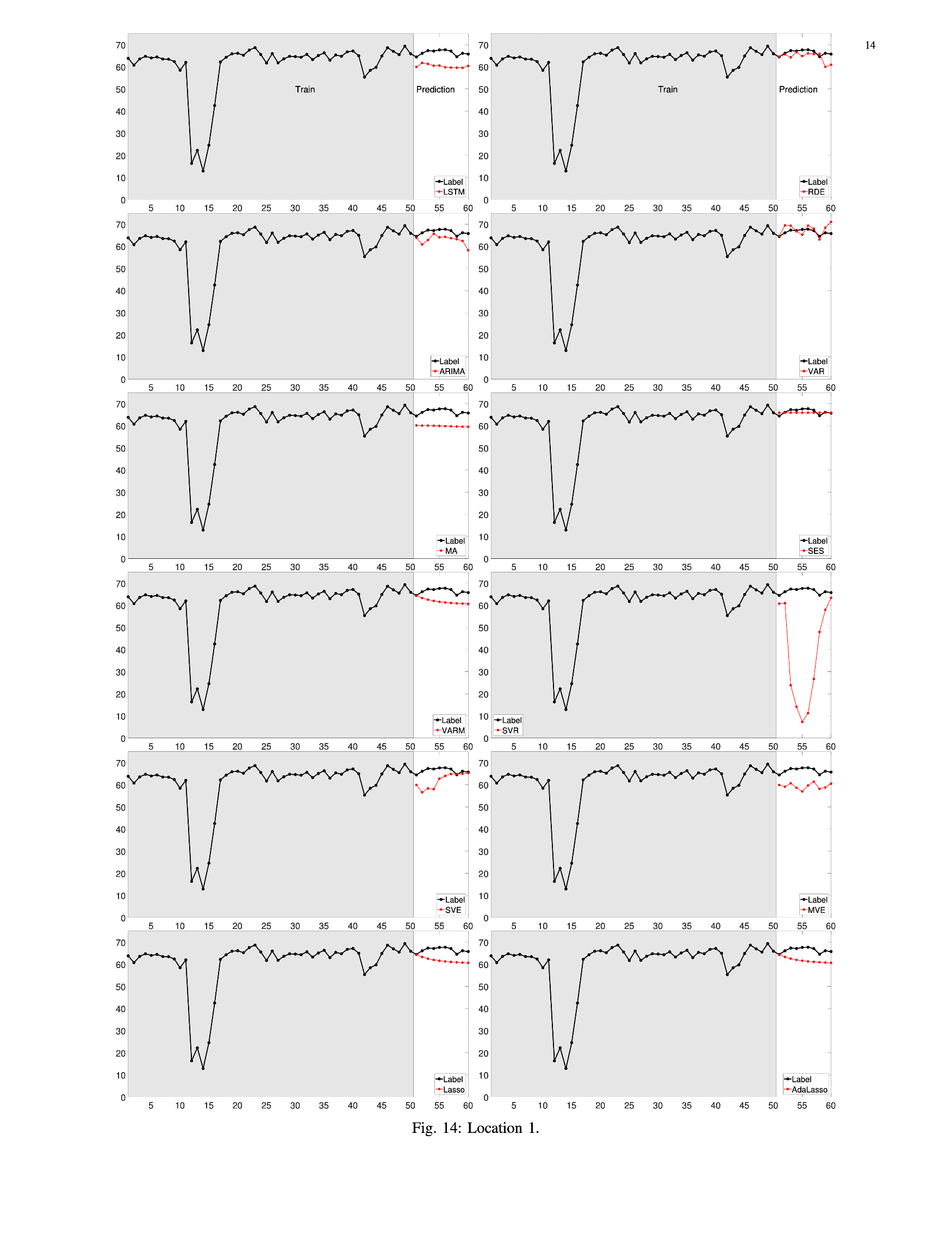


Fig. S23: The performance of all methods to predict Location 1.


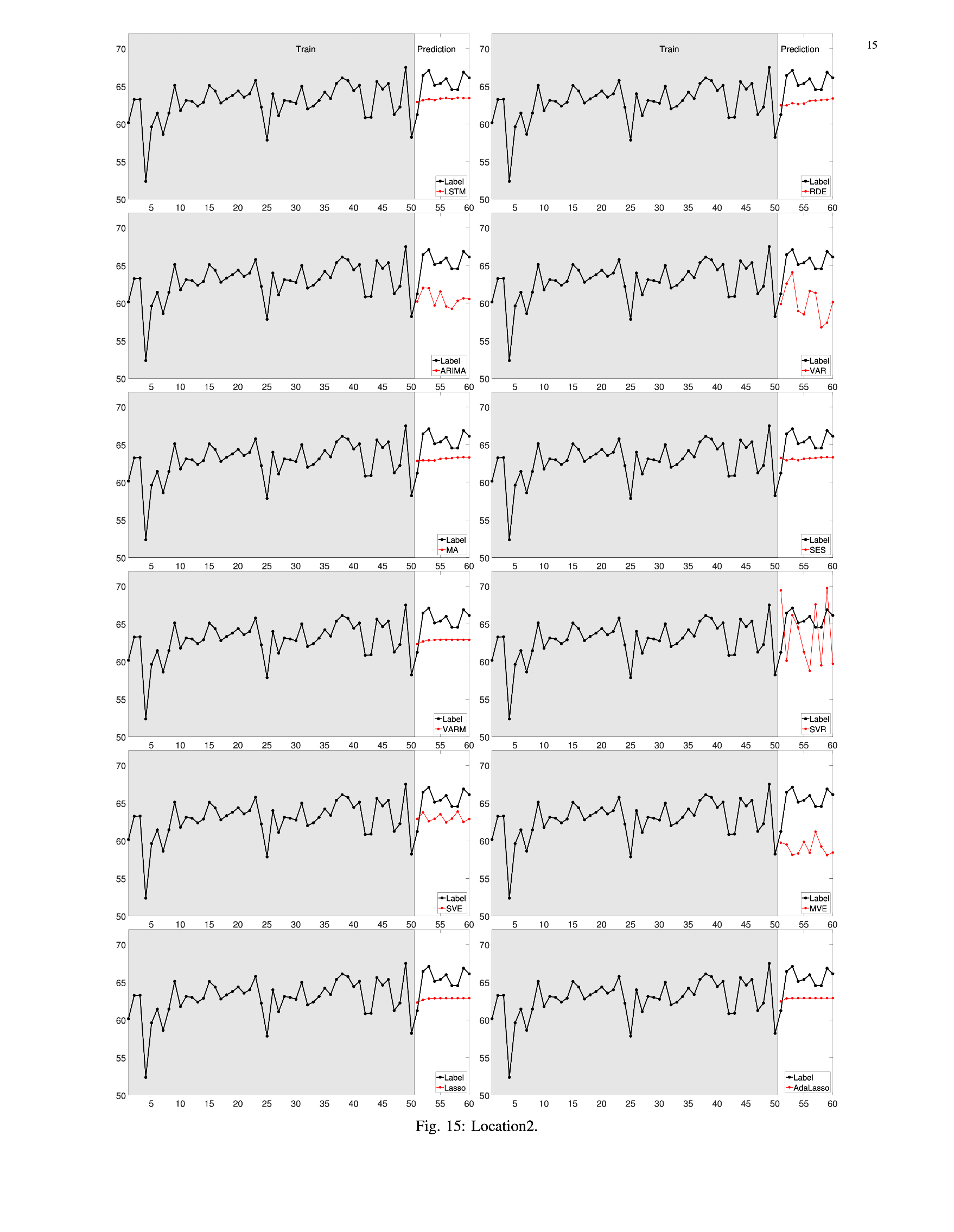


Fig. S24: The performance of all methods to predict Location 2.


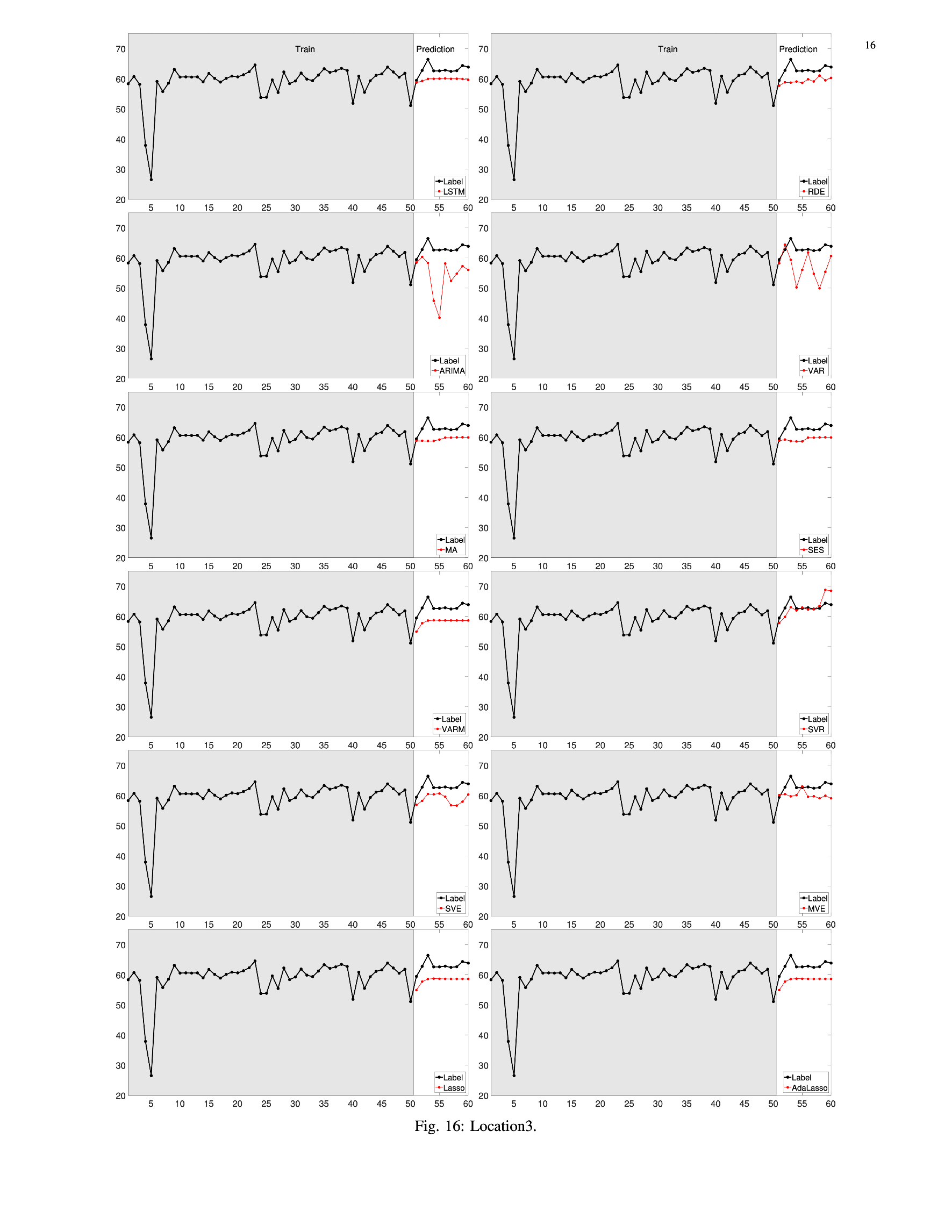


Fig. S25: The performance of all methods to predict Location 3.


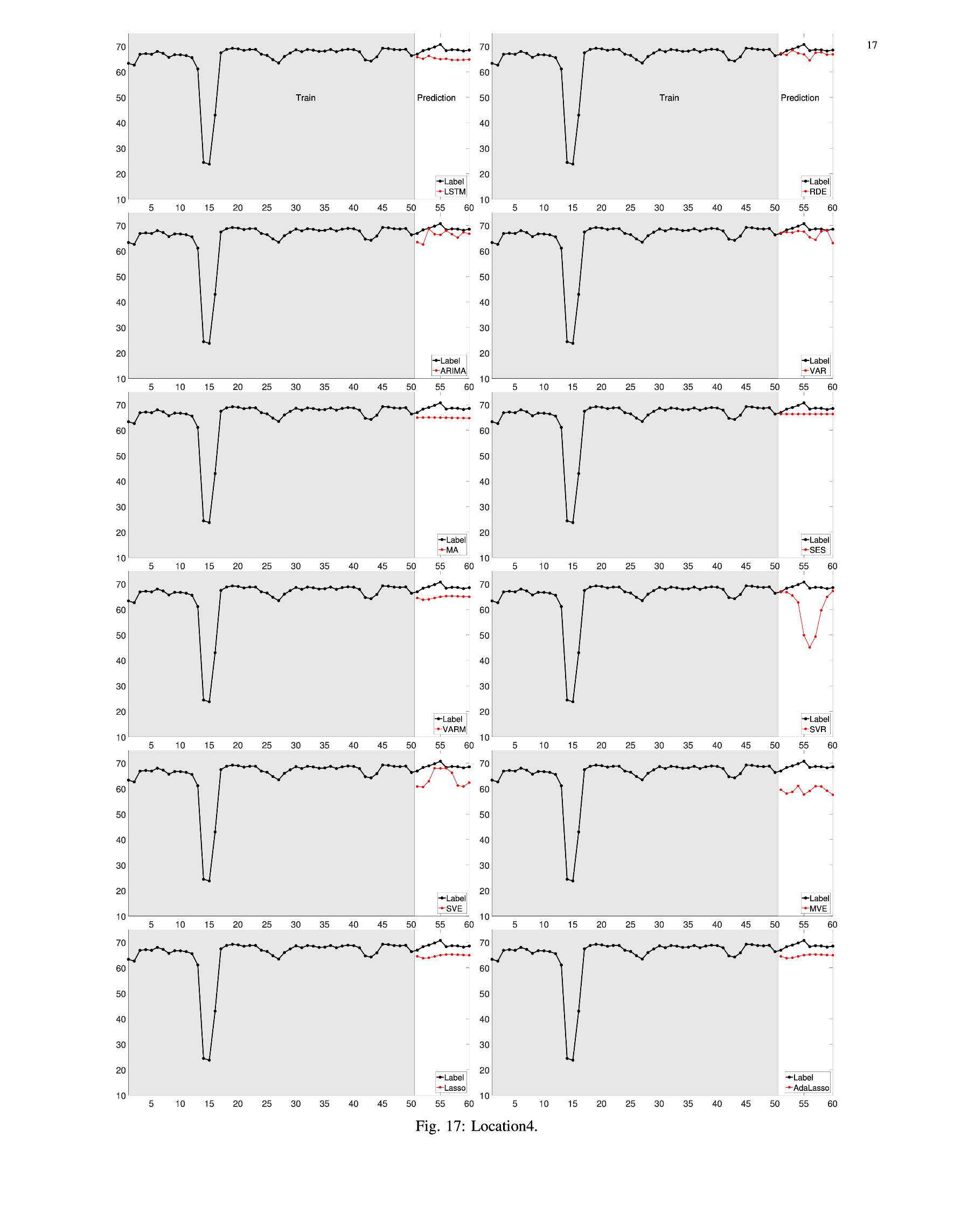


Fig. S26: The performance of all methods to predict Location 4.


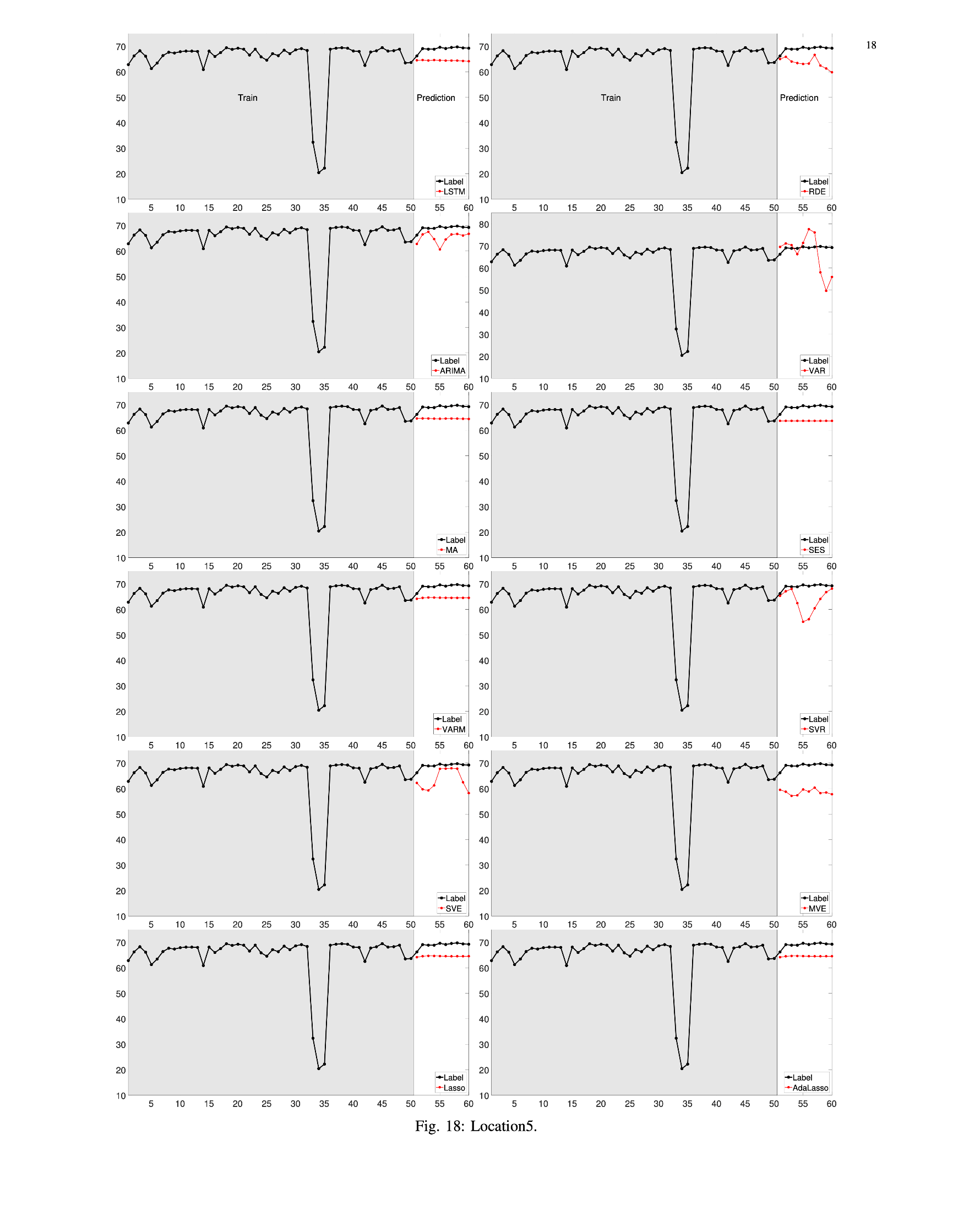


Fig. S27: The performance of all methods to predict Location 5.


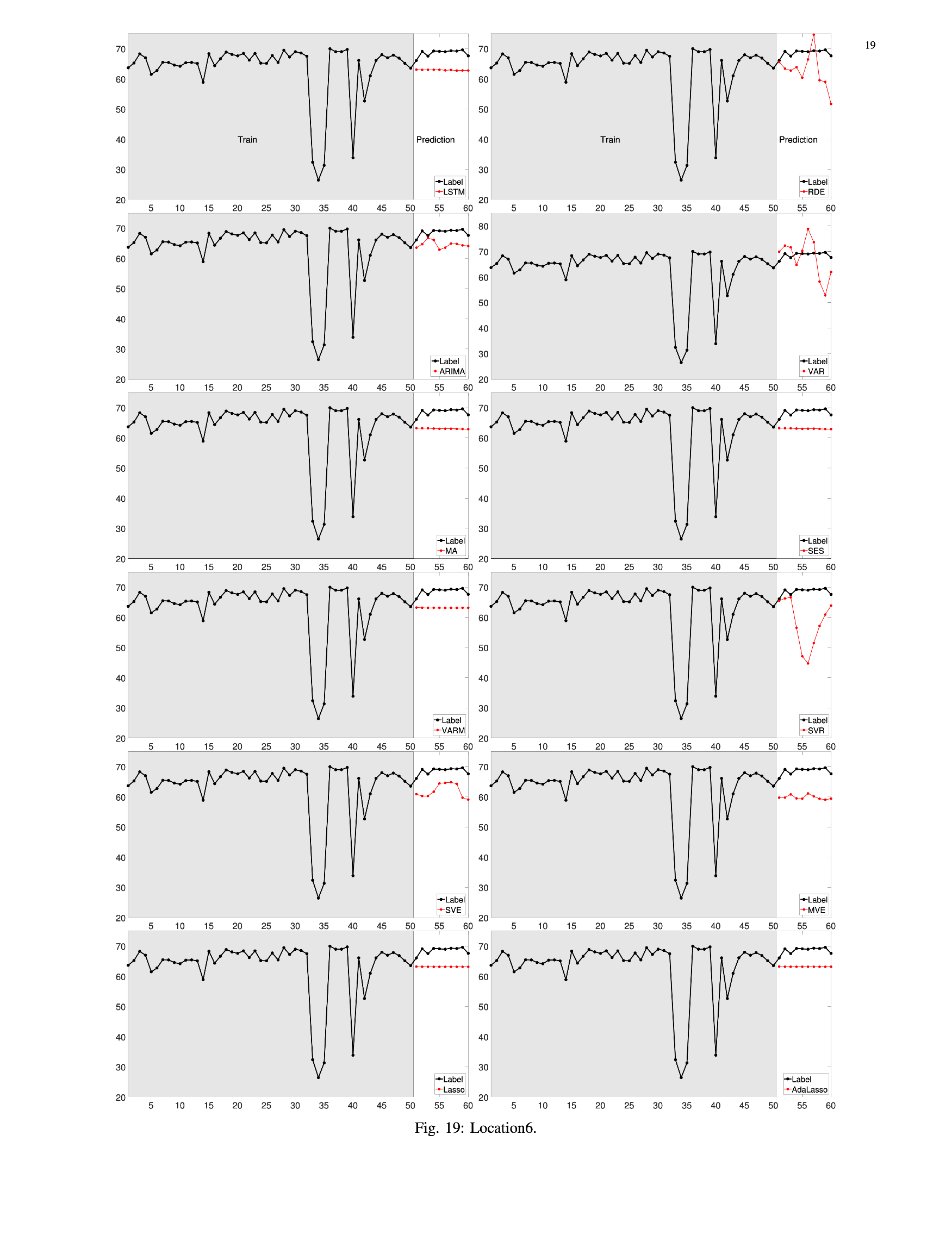


Fig. S28: The performance of all methods to predict Location 6.

- - 1. **Satellite cloud image Dataset**

The MAE, RMSE and Pearson Coefficient Index for each method are shown in Table IX. The predictions for each comparison method are plotted in Figs. S29 – S30. The corresponding movie for the typhoon center prediction is also uploaded to the website:

https://github.com/AnticipatedLearningMachine/Anticipated-Learning-Machine.


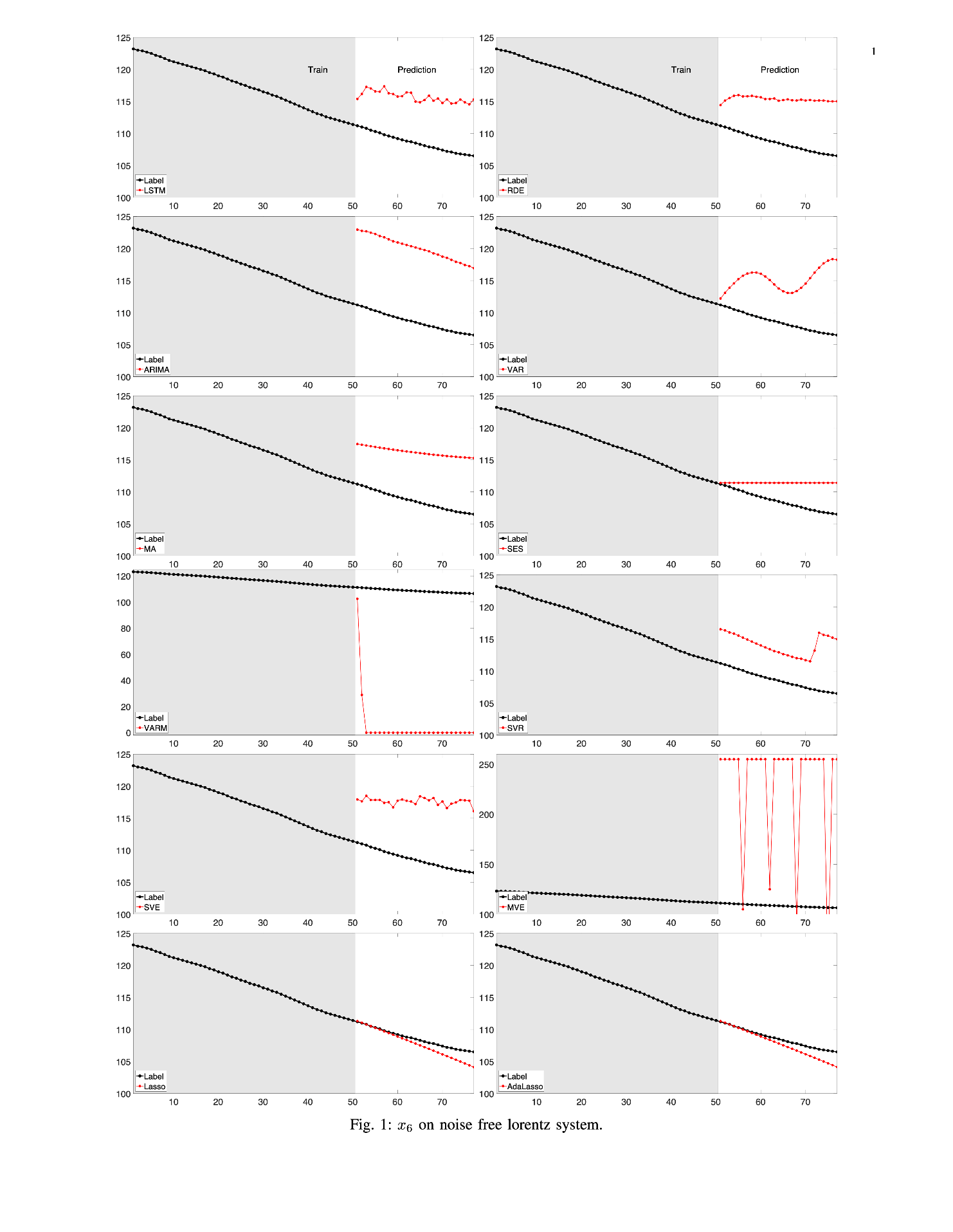


Fig. S29: The performance of all methods to predict the longitude of the typhoon eye.


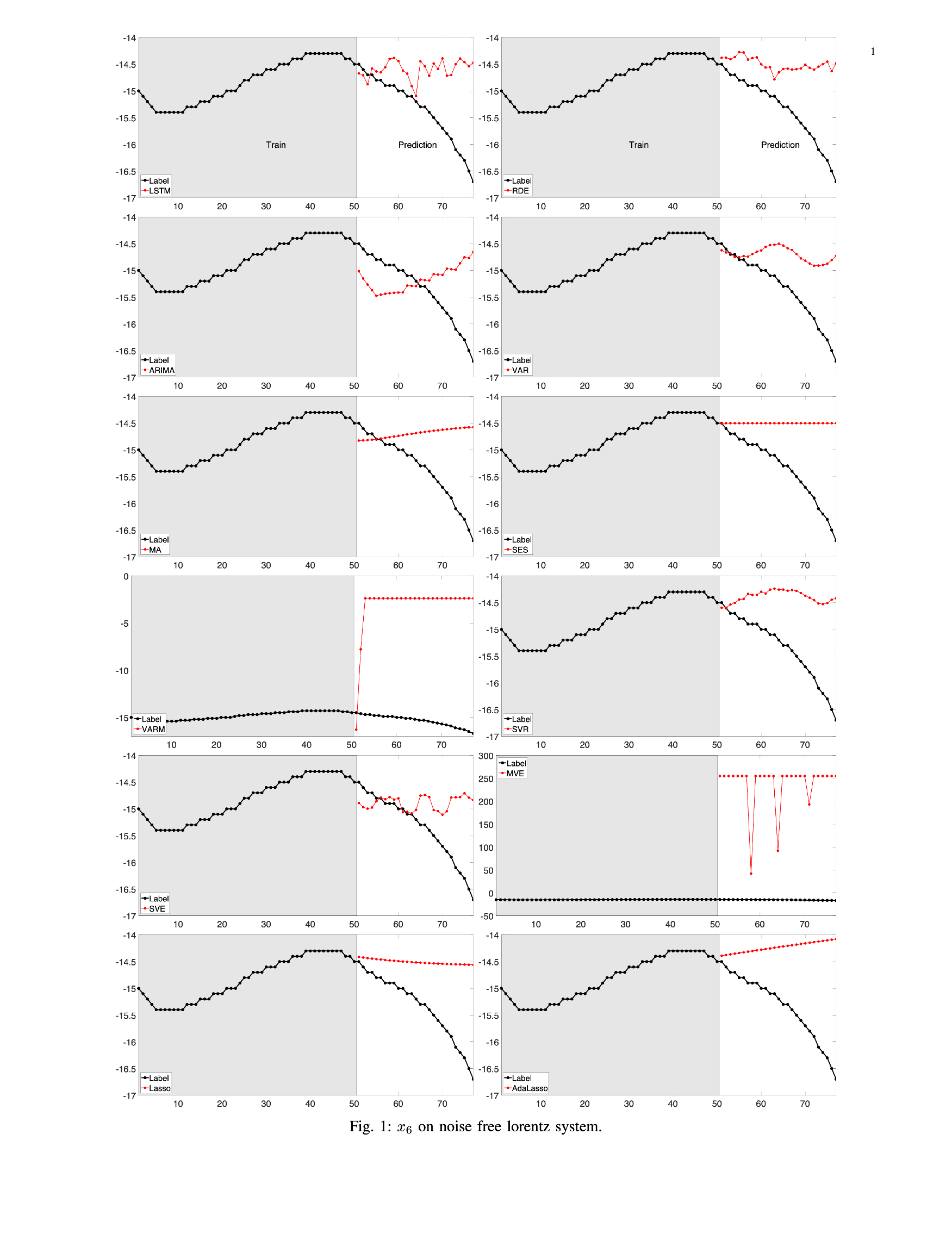


Fig. S30: The performance of all methods to predict the latitude of the typhoon Eye.


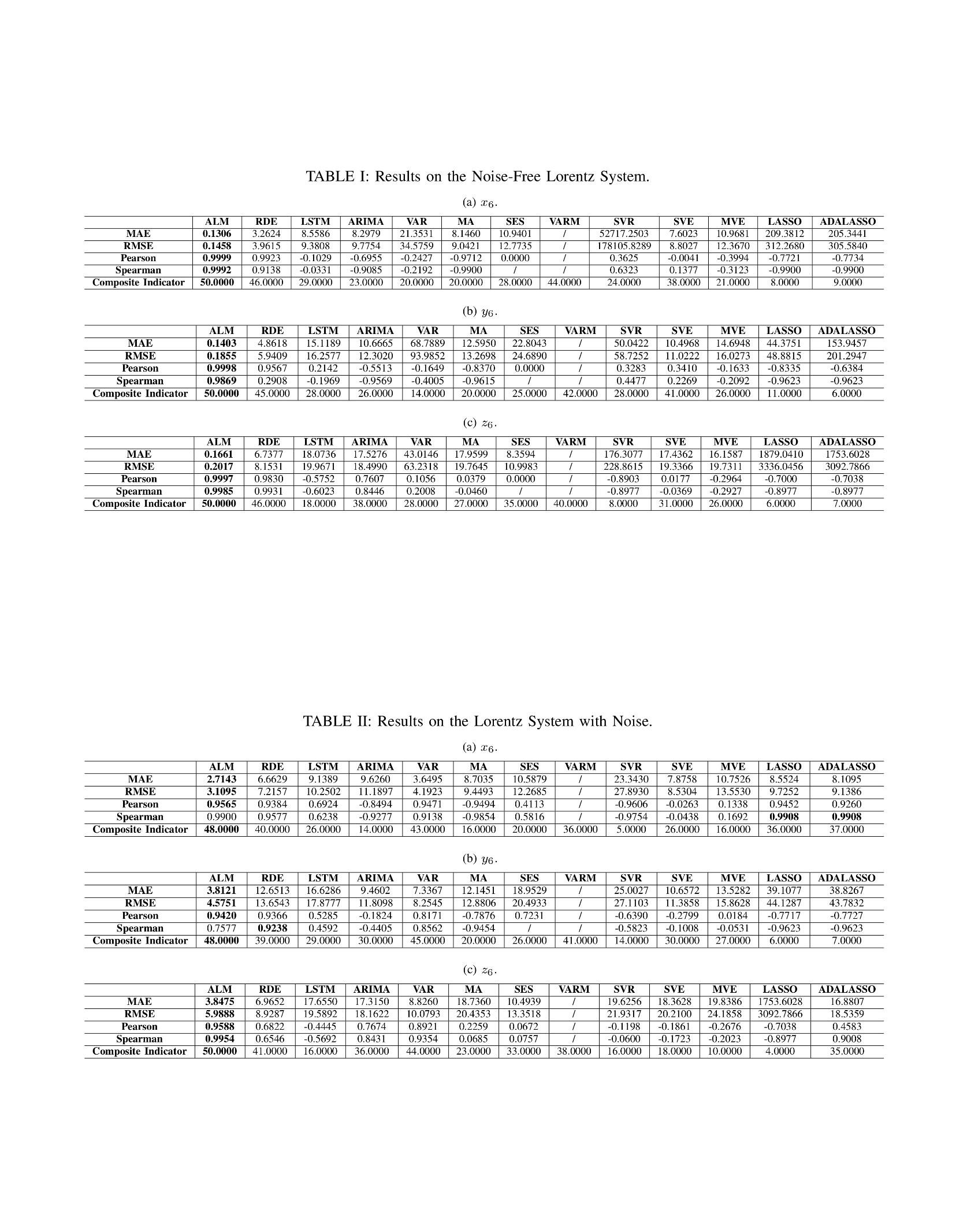


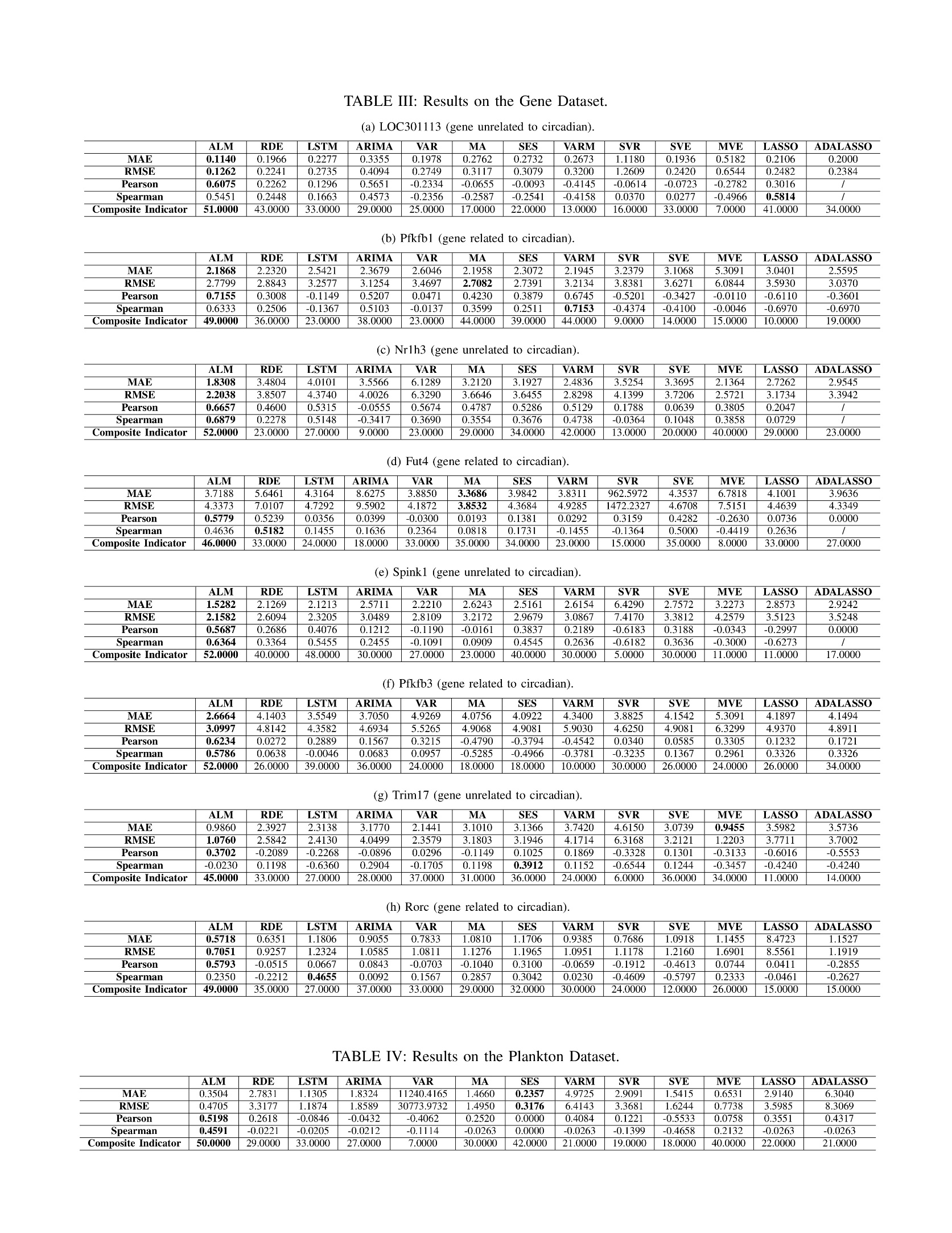


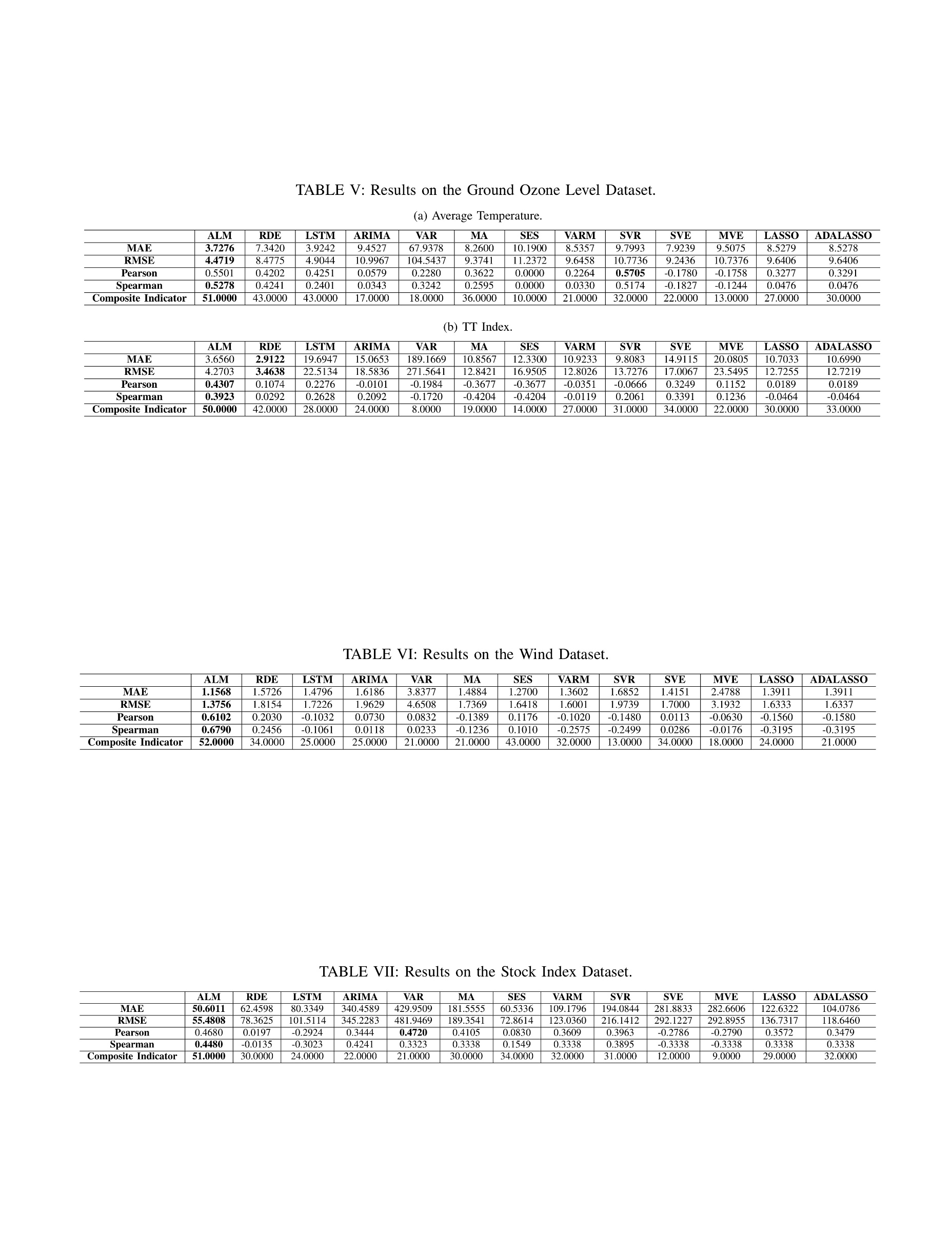


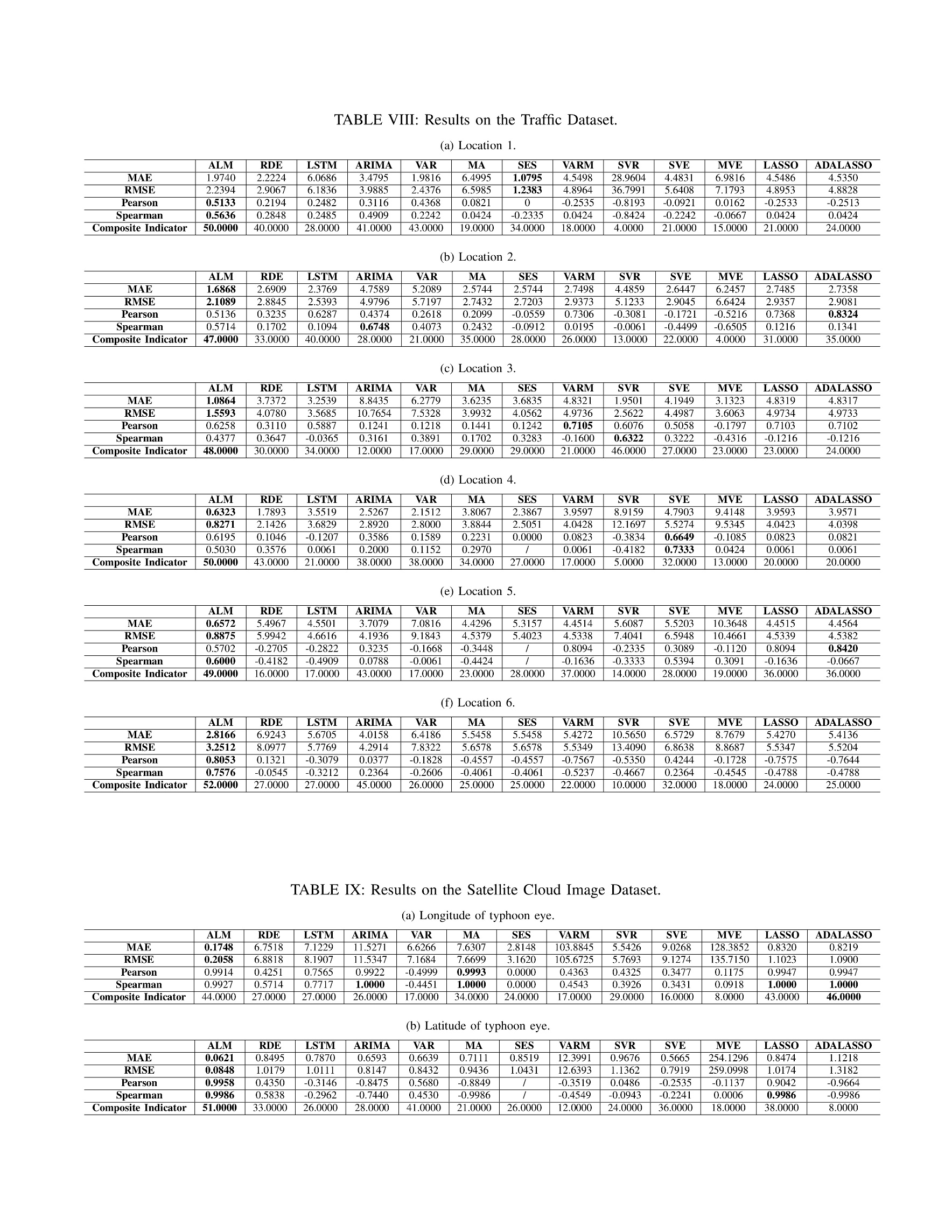


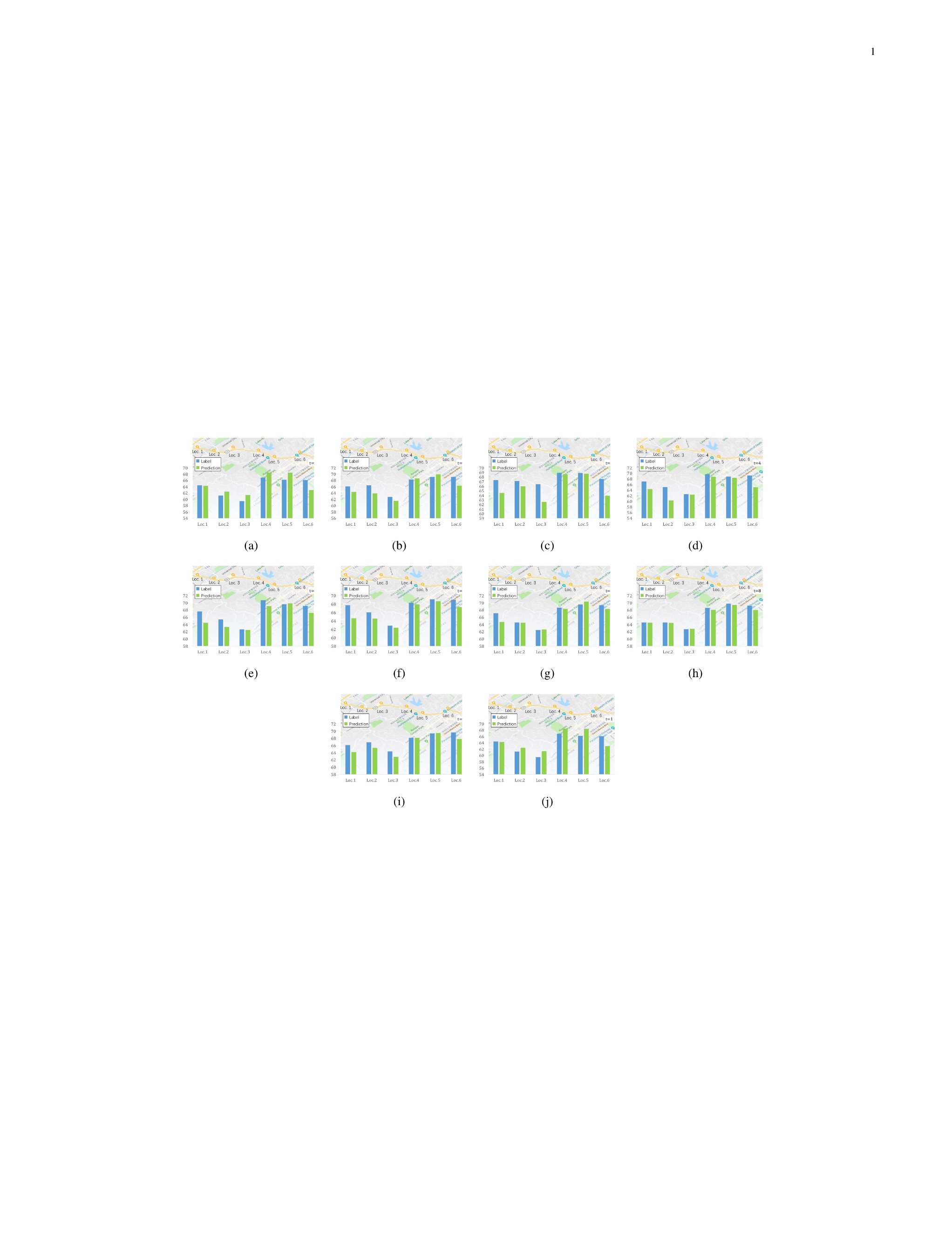


Fig. S31: Prediction of the traffic flow made by ALM.


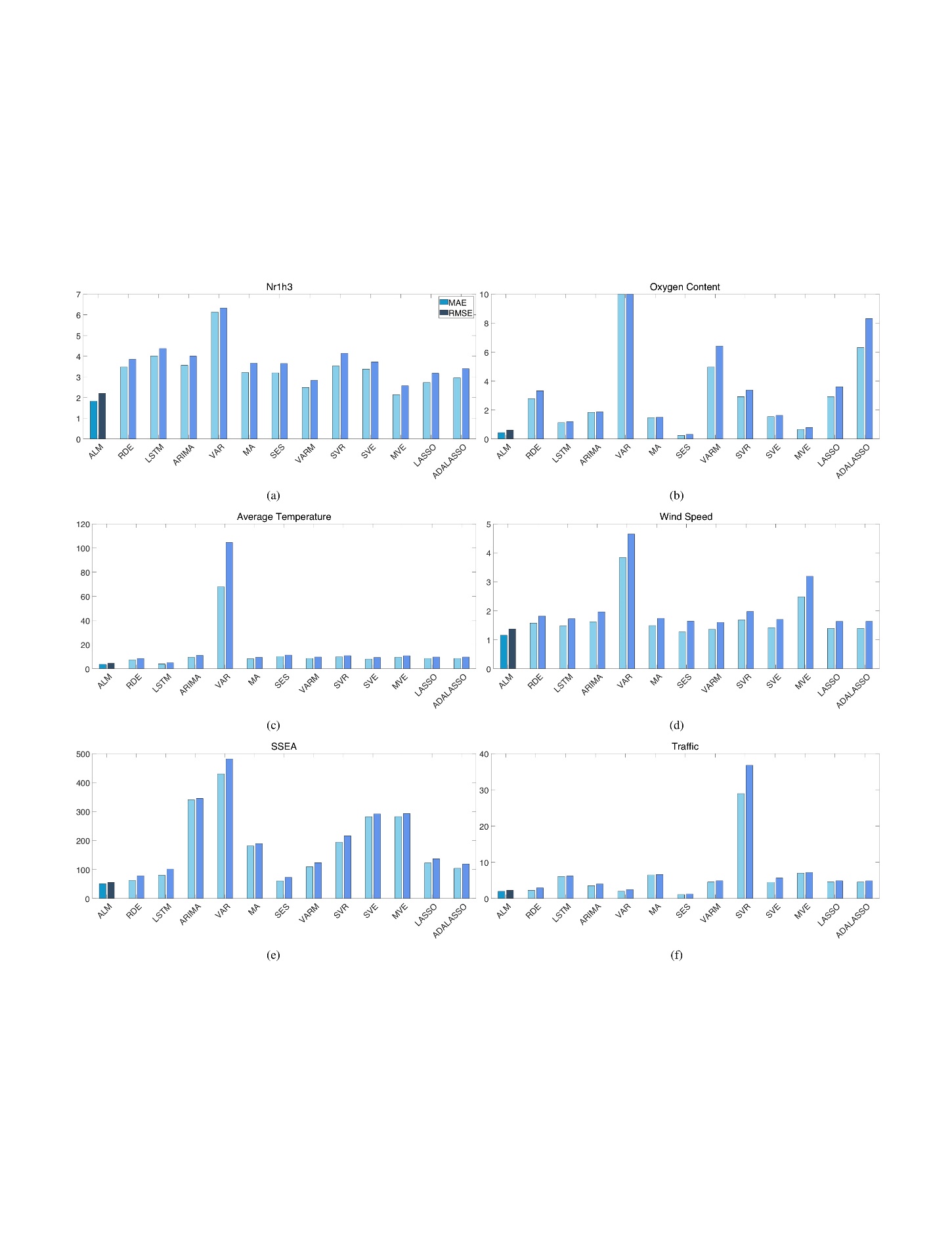


Fig. S32: (a) MAE and RMSE of different methods on the gene dataset. (b) MAE and RMSE of different methods on the plankton dataset. (c) MAE and RMSE of different methods on the ground ozone level dataset. (d) MAE and RMSE of different methods on the wind speed dataset. (e) MAE and RMSE of different methods on the stock index dataset. (f) MAE and RMSE of different methods on the traffic dataset.


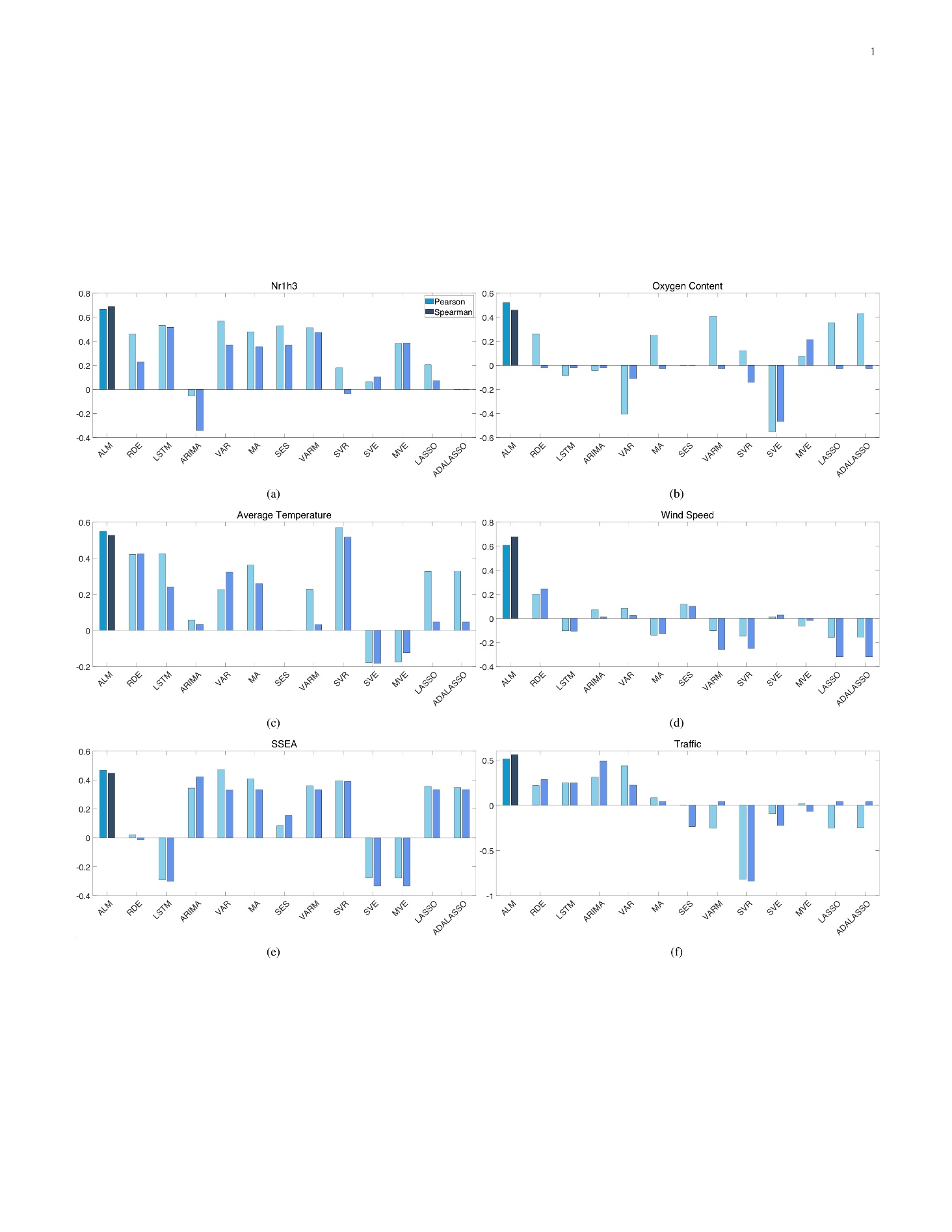


Fig. S33: (a) Pearson and Spearman correlation coefficients of different methods on the gene dataset. (b) Pearson and Spearman correlation coefficients of different methods on the plankton dataset. (c) Pearson and Spearman correlation coefficients of different methods on the ground ozone level dataset. (d) Pearson and Spearman correlation coefficients of different methods on the wind speed dataset. (e) Pearson and Spearman correlation coefficients of different methods on the stock index dataset. (f) Pearson and Spearman correlation coefficients of different methods on the traffic dataset.

**Movie-Traffic & Movie-Satellite Image are attached to the following link:**

https://github.com/AnticipatedLearningMachine/Anticipated-Learning-Machine

**Source code of ALM is attached to the following link:**

https://github.com/AnticipatedLearningMachine/Anticipated-Learning-Machine

**References and Notes:**

1 Takens, F. *Detecting strange attractors in turbulence*. (1981).

2 Deyle, E. R. & Sugihara, G. Generalized theorems for nonlinear state space reconstruction. *PLoS One* **6**, e18295 (2011).

3 Sauer, T., Yorke, J. A. & Casdagli, M. Embedology. *Journal of statistical Physics* **65**, 579-616 (1991).

4 Robinson & James, C. J. N. A topological delay embedding theorem for infinite-dimensional dynamical systems. **18**, 2135 (2005).

5 Ma, H., Zhou, T., Aihara, K., Chen, L. J. I. J. o. B. & Chaos. Predicting time series from short-term high-dimensional data. **24**, 1430033 (2014).

6 Hinton, G. E., Srivastava, N., Krizhevsky, A., Sutskever, I. & Salakhutdinov, R. R. Improving neural networks by preventing co-adaptation of feature detectors. *arXiv preprint arXiv:1207.0580* (2012).

7 Wang, Y., Zhang, X.-S. & Chen, L. A network biology study on circadian rhythm by integrating various omics data. *OMICS A Journal of Integrative Biology* **13**, 313-324 (2009).

8 Kimmel, D. G., Boicourt, W. C., Pierson, J. J., Roman, M. R. & Zhang, X. A comparison of the mesozooplankton response to hypoxia in Chesapeake Bay and the northern Gulf of Mexico using the biomass size spectrum. *Journal of Experimental Marine Biology and Ecology* **381**, S65-S73 (2009).

9 Zhang, K. & Fan, W. Forecasting skewed biased stochastic ozone days: analyses, solutions and beyond. *Knowledge and Information Systems* **14**, 299-326 (2008).

10 Hirata, Y. & Aihara, K. Predicting ramps by integrating different sorts of information. *The European Physical Journal Special Topics* **225**, 513-525 (2016).

11 Li, Y., Yu, R., Shahabi, C. & Liu, Y. Diffusion convolutional recurrent neural network: Data-driven traffic forecasting. *arXiv preprint arXiv:1707.01926* (2017).

12 Informatics, N. I. o. *Digital Typhoon*, <<http://agora.ex.nii.ac.jp/digital-typhoon/summary/wsp/s/201820.html.en>> (

13 Box, G. E. & Pierce, D. A. Distribution of residual autocorrelations in autoregressive-integrated moving average time series models. *Journal of the American statistical Association* **65**, 1509-1526 (1970).

14 Lütkepohl, H. Vector autoregressive and vector error correction models. *Applied time series econometrics* (2004).

15 Winters, P. R. Forecasting sales by exponentially weighted moving averages. *Management science* **6**, 324-342 (1960).

16 Hyndman, R. J. & Athanasopoulos, G. *Forecasting: principles and practice*. (OTexts, 2018).

17 Lütkepohl, H. *New introduction to multiple time series analysis*. (Springer Science & Business Media, 2005).

18 Vapnik, V. *The nature of statistical learning theory*. (Springer science & business media, 2013).

19 Farmer, J. D. & Sidorowich, J. J. Predicting chaotic time series. *Physical review letters* **59**, 845 (1987).

20 Ye, H. & Sugihara, G. Information leverage in interconnected ecosystems: Overcoming the curse of dimensionality. *Science* **353**, 922-925 (2016).

21 Ma, H., Leng, S., Aihara, K., Lin, W. & Chen, L. Randomly distributed embedding making short-term high-dimensional data predictable. *Proceedings of the National Academy of Sciences* **115**, E9994-E10002 (2018).

22 Hochreiter, S. & Schmidhuber, J. Long short-term memory. *Neural computation* **9**, 1735-1780 (1997).

23 Nardi, Y. & Rinaldo, A. Autoregressive process modeling via the lasso procedure. *Journal of Multivariate Analysis* **102**, 528-549 (2011).

24 Patel, Gaurav S. & Eng, B. Modeling nonlinear dynamics with extended kalman filter trained recurrent multilayer perceptrons. PhD thesis, McMaster University (2000).
